# Surveillance and Control Measures during Smallpox Outbreaks

**DOI:** 10.3201/eid1102.040609

**Published:** 2005-02

**Authors:** Emma Kerrod, Alasdair M. Geddes, Martyn Regan, Steve Leach

**Affiliations:** * Centre for Emergency Preparedness and Response, Health Protection Agency, Wiltshire, United Kingdom;; † University of Birmingham, Edgbaston, Birmingham, United Kingdom;; ‡ Health Protection Agency, Liverpool, United Kingdom

**Keywords:** smallpox, modeling, bioterrorism, surveillance, containment, vaccination, immunity, historical review

## Abstract

We reviewed historical data from 2 smallpox outbreaks in Liverpool and Edinburgh during the early and middle years of the 20th century to assess their contribution to developing modern strategies for response to a deliberate release of smallpox virus. Reports contemporaneous to these outbreaks provide detail on the effectiveness of public health interventions. In both outbreaks, extensive contact tracing, quarantine, and staged vaccination campaigns were initiated, and the outbreaks were controlled within 15 months and 3 months, respectively. In Edinburgh, the number of fatalities associated with vaccination exceeded the number of deaths from the disease. In Liverpool, ambulatory, vaccine-modified cases and misdiagnosis as chickenpox resulted in problems with outbreak control. The relatively slow spread of smallpox, as exemplified by the report from Liverpool, allowed for effective implementation of targeted intervention methods. Targeted surveillance and containment interventions have been successful in the past and should be explored as alternatives to mass vaccination.

## Abstract 2

Targeted surveillance and containment interventions have been successful for outbreak control and should be explored as alternatives to mass vaccination.

Heightened awareness of the potential threat of biologic terrorism has generated debate over the most appropriate modeling strategies to assist in planning public health interventions and the required relevant data and assumptions for model parameterization (*1*). A fundamental issue for modeling the potential impact of a deliberate release of smallpox virus is the dearth of recent data. For these reasons, the impact of a bioterrorist release upon a modern population and of the subsequent attempts to contain it are difficult to predict with precision. The dynamics of disease outbreaks in the 21st century, and the outcomes of control strategies used to contain them, have been predicted by using models parameterized with contemporary outbreak data (e.g., measles immunization campaigns). However, to obtain a better idea of how an eradicated disease, such as smallpox, might be controlled requires an analysis of historical outbreak data, much as has been done in a number of studies (*2**–**5*).

Inherent problems are associated with extrapolating past data to the modern day, such as possible differences in susceptibility to infection between modern and historical populations (e.g., immunity) and also potential differences in risk for disease transmission (e.g., changes in contact patterns) (*1*). Nonetheless, when these factors can be addressed properly, the advantages of using historical data as a foundation for modern assessments far outweigh the disadvantages. For smallpox particularly, epidemiologic and outbreak data from the past have been largely relied upon to provide insight into, and evaluation of, the efficacy and efficiency of different public health control strategies for a potential bioterrorist attack.

For example, the levels of protection afforded today by smallpox vaccinations carried out many years ago are difficult to calculate, since few relevant recent assessments exist. A recent study reported stable antiviral antibody and slowly declining antiviral T-cell responses to vaccinia virus in volunteers 1–75 years after vaccination (*6*). How these longer lasting responses correlate with protection from infection itself, from more serious disease, or from death, remains difficult to determine. Natural exposure to the organism is the only way to know whether this response correlates to full (i.e., no disease), or partial (i.e., fewer deaths) protection from smallpox. Since data on natural exposure to smallpox virus are not available for contemporary populations, analysis of historical data is likely to provide the most convincing evidence (*3*).

Historical data on this and other aspects of disease control were published in the early 1900s after a variola major virus outbreak in Liverpool (1902–1903) (*7*) and in the mid-1940s after an outbreak in Edinburgh in 1942 (*8*) (see Appendix). These reports form the basis of this article, which discusses the use of historical data in predictive assessments of disease events. The Liverpool smallpox outbreak data are included in a large section specifically on smallpox in the annual Health Department Report for the city written by the Medical Officer of Health in 1903, at a time when smallpox was still endemic in Liverpool. The report covers all aspects related to health, ranging from typhus and tuberculosis to rainfall, temperature, and demographic statistics. Supplementary information on this outbreak has also been taken from Appendix 10 of the Annual Report of the Medical Officer of The Local Government Board 1904-05, in Report on Smallpox and Smallpox Hospitals at Liverpool, 1902–03, which investigated specific aspects of the outbreak for the local government board (*9*). A further report has also been used, written in 1913 by the assistant medical officer of health for Liverpool; it reports in greater detail on the effects of the disease in relation to the impact of vaccination and includes a large series of cases from the 1902–1903 outbreak (*10*).

The report on the 1942 outbreak in Edinburgh also provides data on a range of important aspects of smallpox control, including adverse events to vaccination (*8*). This large report was written in 1944 by the medical officer of health and his colleagues at a time when smallpox was no longer endemic in the region. The stated purpose of the report was to provide information for medical staff in the event of future outbreaks. The information detailed, therefore, is more descriptive than that in the Liverpool publication but provides more data on the clinical and control aspects used. Again, supplementary articles have been consulted, primarily those concerning the contemporaneous outbreaks in Glasgow and Fife that led up to the Edinburgh outbreak. A close evaluation of the 2 outbreaks illustrates the value of using historical data when considering public health control and containment strategies for potential bioterrorist events.

## Outbreaks

**Figure 1:**
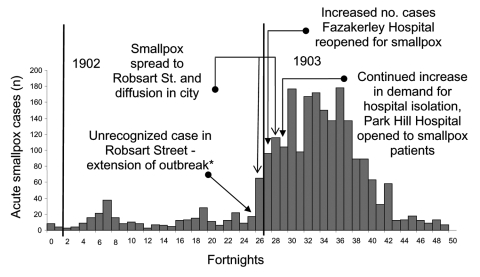
Timeline of Liverpool outbreak: key events and control interventions, using hospital admission data (December 6, 1901–November 27, 1903) (*9*). *House-to-house visitation of the district "forthwith commenced" (*7*). Over the next few days, 20 more cases were found and reported.

Since the 1860s, Liverpool had had cases of smallpox (*7*). According to the 1904–1905 report, seaports were prone to occurrences of smallpox, and therefore, Liverpool had "abundant opportunities of perfecting its administration in regard of this disease" (*9*). Although the annual number of cases had declined considerably in the 17 years or so before the outbreak began in 1902, a total of 23 cases were imported by sea and 16 were introduced by "vagrants." However, according to 1 researcher, an epidemic broke out toward the end of 1902 (*10*). The outbreak lasted from October 1902 to the end of December 1903 and resulted in 2,032 cases and 161 deaths (case-fatality rate = 8%). The first smallpox case occurred in 1901 and resulted directly from an imported case-patient, a merchant seaman. This importation brought the disease into Liverpool at the end of 1901, a year in which, until that time, practically no smallpox had been reported (*9*). The administrative actions of the Public Health Department of Liverpool checked the spread of smallpox until November 1902, when an unrecognized case-patient (*7*), an infant, was medically attended only when the child was dying of the disease. In addition, 6 infected household members were found, and subsequent house-to-house inquiries in the district discovered another 20 clinical case-patients during the next few days, most of whom were friends of the infected family (*7*). This number of cases is assumed to have resulted from chains of transmission beginning with the infant and spreading through the family and to wider contacts, rather than transmission from the child directly to 26 others. Despite attempts to prevent further spread, the number of cases in the locality reached 99 by the end of January 1903. The disease then continued to spread to the east and south of the city, with the monthly number of cases peaking at 356 in March 1903. The timeline of the outbreak in relation to a number of other key events and control measures is shown in Figure 1.

**Figure 2:**
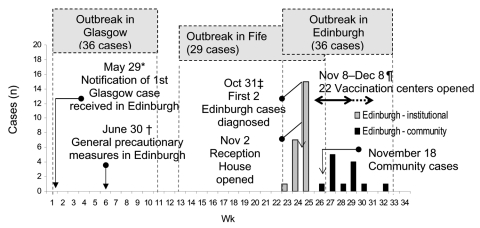
Timeline of Edinburgh outbreak. *May 29: Revaccination of all Edinburgh medical, nursing, domestic, artisan and ambulance staff. Edinburgh Smallpox Hospital reconditioned and isolation units set-up for observation cases (*8*). †June 30: great majority of other essential personnel vaccinated (*11*). Some public vaccination by private practitioners (≈4% [20,000]). ‡November 1: quarantine and daily surveillance of (present and past) patients and visitors to Royal Infirmary (*8*). ¶November 8–December 8: further vaccination centers opened after 3 more cases occurred. Sixty sessions held each day. One vaccination center reopened December 9–12 and December 21–24 to cope with a few isolated cases (*8**,**11*).

Similarly, until 1905, Edinburgh had also seldom been free from smallpox. Later, however, smallpox outbreaks became infrequent, with only 4 outbreak years from 1905 to 1920, and then none at all in the 20 years before 1942. The outbreak, therefore, was a relatively new experience for a large section of the population (*11*). This outbreak was relatively small and lasted 3 months (October 27–December 30, 1942), which resulted in 36 cases including 8 deaths (case-fatality rate = 22%). Smallpox had previously been imported into Scotland on May 29, 1942, by a ship arriving from Bombay into Scotland's other major city, Glasgow (resulting in 36 cases and 8 deaths) (*12*). In August, 3 weeks after the last case in Glasgow, an outbreak was reported in Fife (29 cases and 8 deaths). As the outbreak in Fife was being brought under control, the first case of smallpox appeared in Edinburgh Royal Infirmary. The disease then spread to the hospital's convalescent home and then into the general public. The means of the spread of disease from Glasgow to Fife and then to Edinburgh, and from hospital settings to the general public, was, however, never identified. Indeed, for 8 of 13 Edinburgh community cases, the source of infection was never discovered. The author of the outbreak report conjectured that subclinical infections, i.e., "mild attacks" or missed cases, might have been the reason for the lost epidemiologic links but adds that these theories were hard to reconcile with the facts (*8*). A timeline of the Edinburgh outbreak, highlighting the milestone events and control measures employed, is shown in Figure 2.

## Vaccination Status of Population

To appreciate the course of the outbreaks and the subsequent effects of the various control measures used, the vaccination history of the populations involved should be put into context. If we assumed that the level of infant vaccination in Liverpool was similar to that for England and Wales as a whole in 1902 and 1903 (≈75%) when solid immunity in the city might have ranged from 9% to 16% (solid immunity, as termed by Dixon [13], is either 5 or 10 years of total protection from attack). However, at that time, considerable support for the antivaccination cause resulted in an infant vaccination rate in the late 19th century that varied from 0% in some districts to nearly 100% in others (*13*), and as the background rates for Liverpool are not reported, being more specific about the levels of vaccination that existed is difficult.

Vaccination levels also varied from region to region in Scotland. The percentage of vaccinated infants in Scotland was normally ≈30.7% (*14*); however, the Registrar General for Scotland reported that 55% of infants were being vaccinated in 1941. Whether this report is for Scotland as a whole or for Edinburgh alone is not clear (*8*). Dixon's estimate of solid immunity for the whole of England and Wales in 1947, assuming 40% of infants were vaccinated, was 4%–7%. However, this percentage was increased by the vaccination of National Service entrants to ≈20%. For Scotland, with an infant vaccination rate of ≈30%, solid immunity would have been <20% (*13*). The vaccinial state of the public as a whole was reportedly low in the area around Fife (Methilhill), with only 20%–30% of the population having been previously vaccinated; but in towns nearer Edinburgh (e.g., Cowdenbeath) 40%–50% had been vaccinated (*14*).

## Public Health Response

**Figure 3:**
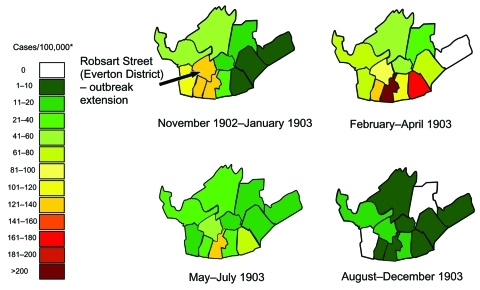
Spatial-temporal distribution of incidence of smallpox during outbreak, by district, Liverpool, 1902–1903 (*7*). *Incidence of smallpox per district (per 100,000) calculated as number of cases per district ÷ by district population x 100,000. New cases per district were counted from the locations given on the 4 maps in the original report for each of the periods above. District populations were tabulated separately (*7*).

In both Liverpool and Edinburgh, phased public health responses were implemented (Figures 1 and 2). In Liverpool, at the earliest phase of the outbreak, with the discovery of the first unreported case in Robsart Street (Figures 1 and 3), active case finding in the local area was instituted. One report states that, thereafter, usually within an hour of notification, patients were removed to hospital by ambulance, and the clothing, bedding, and dwellings were immediately disinfected (*9*). An inspector followed the ambulance and immediately made inquiries about possible sources of infection. Information about the state of vaccination of possible contacts was then sent to vaccination officers; additional medical staff members were employed at this time to assist with vaccination. These vaccination officers in Liverpool first recommended immediate vaccination or revaccination to all close contacts of case-patients, then to related workforces, schools, and the general public. Special arrangements were made for the prompt vaccination of all vagrants coming into the city, who were subsequently paid a small sum for consenting. Offers of vaccination and revaccination to contacts and people living close to persons with smallpox were reported in the Health Department Report to have been promptly made and almost universally accepted; these vaccinations were reported to have greatly limited the amount of smallpox in Liverpool. As the number of cases increased as the outbreak developed, hospital isolation accommodations were expanded by committing an increasing number of hospitals to the intake of smallpox patients (Figure 1).

In Edinburgh, after notification of the first Glasgow case on May 29 (Figure 2), the first campaign of vaccination and revaccination for essential personnel (e.g., medical staff, civil defense workers, and police), was agreed to on June 30 and promptly instituted on July 1, 4 months before the disease reached Edinburgh (*11*). Edinburgh had not had a smallpox case for 20 years, but at this time, the smallpox hospital was reopened, and satellite isolation units in the hospital grounds prepared to receive patients for observation. All contacts of Glasgow case-patients arriving in Edinburgh were, as was routine practice, examined and put under surveillance. The Public Health Department was responsible for the medical supervision of contacts, and medical officers of health were responsible for requesting precautionary behavior in the general public. The second vaccination campaign took place from November to December 1942, when the disease had taken hold in Edinburgh itself. At this time the contacts of patients were vaccinated, and vaccination was extended subsequently to the general public with the opening, on November 8 (Figure 2), of 22 vaccination centers throughout the city.

Despite the previous vaccination of infants and other target groups, levels of immunity contemporaneous with these 2 outbreaks were insufficient on their own to prevent expanding outbreaks. Nevertheless, the spread of infection over both space and time across Liverpool was characteristically slow, taking 3 months to significantly extend out of the district into which it was introduced, to more southeastern districts (Figures 1 and 3).

The first 2 cases in Edinburgh were diagnosed on October 31. On November 1, active case-finding was initiated with house-to-house searches, and a first aid post was opened subsequently, which provided 8,000 vaccinations to people in the area in which these patients lived. Family contacts of patients were sent for observation to a prepared reception house, which was opened on November 2, the day after the first 2 cases had been confirmed (Figure 2). Persons in the reception house were quarantined for 21 days, and all but 1 of their employers agreed to pay their wages during this time (*8*). The exception to this rule attended work during the day and returned to the reception house at night and was examined both upon leaving and on returning for signs of infection. The Royal Infirmary convalescent home also acted as an additional observation ward.

Contact tracing was an important part of the control methods instituted in both outbreaks. In Edinburgh, the press was used extensively as a means to trace contacts of case-patients and to persuade large numbers of persons to accept vaccination; the use of the press also allowed the authorities to reach possible contacts with a minimum of delay (*11*). In all, ≈1,700 contacts of the 36 cases were traced and observed for 18 to 21 days, which represents an average of ≈47 contacts per case. More than 900 persons were traced as contacts and revaccinated from 3 cases alone. Despite being infected, these ambulatory cases had used public transport or been in contact with large numbers of persons because of their occupation (*8*). The readiness of the public to cooperate with all the above recommended, routine precautions is noted in the Annual Report, 1942 (*11*). The press was also used in the Liverpool outbreak. Circulars that detailed the movements of case-patients who had used public transport and the location and availability of public vaccinators were widely distributed. Although the total number of traced contacts is unclear, we know that contacts were visited every day for 14 days after notification, and then every few days for a further 2 weeks. At the peak of the outbreak, when 356 cases existed, as many as 2,000 families were being visited daily, which represents an average of ≈6 families contacted per case. On the basis of an average household size for England, at that time 5, we have a rough estimate of 30 contacts traced and vaccinated per case.

In the Liverpool outbreak, the occurrence of a large number of vaccine-modified cases caused particular problems for those attempting to control the outbreak, especially with respect to late or incorrect diagnoses. According to Hanna (*10*), 72.7% of those vaccinated previously and 16.8% of unvaccinated cases were considered to be "modified discrete and discrete smallpox" (modified here meaning an accelerated clinical course compared with expected course of ordinary smallpox, usually with fewer lesions, not necessarily modified by vaccination) (*13*). Chickenpox was a notable misdiagnosis in some instances; 2.6% of chickenpox diagnoses were found subsequently to be smallpox (similarly, in the 1901–1902 London outbreak, the figure for the same misdiagnosis was 2.5%). To help overcome this problem, chickenpox was made a notifiable disease, provisionally in April, and permanently in August 1902. Reporting of smallpox itself, however, was not always straightforward in the Liverpool outbreak. According to 1 author (*9*), the diagnosis of smallpox was sometimes revoked upon admission to hospital, or vice versa, a nonsmallpox case-patient was often treated as having smallpox in the hospital. On at least 1 occasion, information on patients treated in the hospital did not reach the medical officer of health. In Edinburgh, the first 2 cases were misdiagnosed as chickenpox and meningococcal septicemia, respectively. Misdiagnosis as chickenpox is a concern that continues to exist today. In Glasgow, in 1942, severe vaccinial reactions, occurring at the end of the possible incubation period of smallpox, also complicated the problem of diagnosis for medical practitioners (*12*).

As with the 3 ambulatory case-patients in the Edinburgh outbreak discussed above, such patients were also a problematic source of infection in Liverpool (*7**,**10*). Some smallpox infections were reported to be so mild (usually vaccine-modified) that doctors were not consulted, and patients and their household contacts continued to visit public areas and shops. For example, 1 unreported smallpox case occurred in a person whose family continued to go to work and socialize, which gave rise to 29 other cases. Twenty prosecutions were instituted against members of this family during the outbreak. The extent to which ambulatory vaccine-modified cases might occur in any modern day U.K. outbreak is not known. However, the proportion of vaccine-modified cases overall would be much less than in Liverpool because of the length of time since the population was last vaccinated. This finding has been discussed in greater detail elsewhere (*2*).

**Figure 4:**
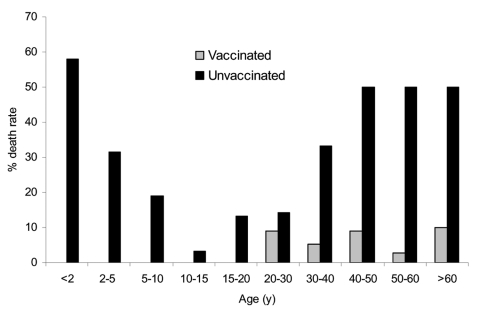
Percentage case-patient death rate by age in the vaccinated and unvaccinated, Liverpool outbreak, 1902–1903 (*10*).

Previous vaccination status also strongly influenced the relationship between age at time of attack and death (Figure 4). In a study that examined a series of 1,163 case-patients during the 10 years after the Liverpool outbreak (mostly from the epidemic period 1902–1903), 943 (81%) had been vaccinated in infancy, and 220 (18.9%) had not been vaccinated (*10*). Among those vaccinated in infancy, 28 (2.9%) deaths occurred from smallpox, whereas among the unvaccinated, 60 (27.2%) deaths occurred. The case mortality among the vaccinated rose steadily with age from the 20- to 30-year age group upwards to the >60-year group (no deaths occurred in those <20), but never exceeded 10%. However, among the unvaccinated, 58% of patients <2 years of age died, decreasing to 30.6% for those 2 to 5 years of age. The ratio was lower (3.2%) for those 10 to 15 years of age; the case-fatality rate rose (13%) for those 15 year of age, and it was 50% for those >40 years of age. The effect of vaccination on protection against death according to age has also been noted by others (*15**,**16*). The level of partial immunity to smallpox, i.e., protection from death as opposed to protection from infection, in a modern population may be higher than previously thought (*1*); spread of infection from ambulant patients with vaccine-modified cases may be an important and problematic means of transmission (*10**,**13**,**17*), as has been pointed out in more recent analyses (*2*). In the Edinburgh outbreak, 6 of the 8 deaths from smallpox occurred in adults >20 years of age who had been vaccinated in infancy.

No data concerning vaccine-related adverse events are available from the Liverpool outbreak, but we know that of the estimated 360,000 vaccinations (based on lymph issue) performed in Edinburgh and adjacent counties (≈77% of the local population), 10 vaccine-related deaths occurred; 8 of these were from encephalomyelitis. Compared to vaccination campaigns in England and Wales in 1951 to 1960 (*18*), the numbers of postvaccinial encephalomyelitis and generalized vaccinia were much higher (Table). Indeed, a similarly high incidence of postvaccinial encephalomyelitis was reported during the Fife outbreak (*14*). Approximately 78% of vaccinees in and around Edinburgh had had a previously successful vaccination; the remainder, ≈22%, had either a previously unsuccessful vaccination or no vaccination at all. In neither of the outbreaks was an intensified national vaccination campaign reported to have been initiated.

**Table:** Rates of adverse events to smallpox vaccination in Edinburgh, 1942*

Adverse event	1st vaccination campaign Jun – Jul†	2nd vaccination campaign Nov – Dec†
Nonspecific rashes	20	12.5
Auto-inoculation and generalized vaccinia	7.5	7.5
Postvaccinial encephalomyelitis	5.0	4.7

*Source: reference 8. †Per 100,000.

## Discussion

In both the Liverpool and Edinburgh outbreaks, phased public health responses were implemented, and the outbreaks were brought under control within 15 and 3 months, respectively. Because smallpox arrived first in Glasgow, Edinburgh health authorities had time to prepare and implement a 2-phased vaccination campaign along with active surveillance. For Liverpool, the report demonstrates clearly that the spread of infection across the city was slow, which suggests a relatively low transmission rate and a characteristically long generation time, allowing for targeted intervention methods to be effectively implemented. By comparing the incidence of cases in different districts across the panels shown in Figure 3, the outbreak appears to have taken 3 months (November 1902–January 1903) to spread into districts adjacent to the origin of the outbreak and then an additional 3 months (February–April 1903) to spread to more eastern and western districts. The slow spread of smallpox described here is not dissimilar to that described in studies in other countries, for example, Pakistan and Bangladesh in the 1960s (*17*). In Bangladesh, smallpox tended to be more rapidly transmitted within family units but spread more slowly between them (*19*).

Active surveillance, vaccination of contacts, and prompt hospital isolation of patients were important aspects of disease control measures in both outbreaks. Indeed, the success of the surveillance-containment strategy in Liverpool, the basis of which has been discussed more recently elsewhere (*20*), was particularly noted by the observers of the time (*7**,**10*). Unlike the situation in the United Kingdom today, both Liverpool and Edinburgh had designated smallpox hospitals, either already open or ready to reopen, at the time of these outbreaks. These dedicated facilities must have contributed to infection control efforts. However, control and containment procedures in the 2 cities were hampered in both outbreaks to some extent by reintroduction of the disease from other areas, by patients with ambulant cases of mild infection (probably vaccine-modified), and by missed cases.

These 2 case studies draw attention to issues of current concern, not only to the potential impact of vaccine-modified cases mentioned above, but also to adverse events to vaccination, both of which might have an impact in a modern-day outbreak. However, in contrast to these 2 outbreaks, the fact that routine smallpox vaccination ceased in the West during the 1970s brings complications of its own. Persons <30 years of age have never received the vaccine and are immunologically naïve. This 30-year time gap since vaccination also has implications for the immune status of previous vaccinees and the potential for adverse event and disease complications and indeed for the spread of disease among this population. If historical events are to be used as sources of evidence, and the data from them extrapolated to modern populations, they must be considered within the ethical and social context of today, by observing societal differences, expedited travel, waning immunity, and increased recognition of contraindications to vaccination. In particular, the number of people who are immunocompromised today continues to rise with the increase of HIV infection, chemotherapy, immunity disorders, and transplantations. So too has the number of people with atopic dermatitis; in the United Kingdom alone, 2.3% of the population is estimated to have this condition (*21*). However, cardiac adverse events to vaccination, such as myocarditis and pericarditis, were not reported in these 2 case studies, as has been seen in more recent vaccination efforts (*22*).

The studies also illustrate that the level of background solid immunity in these populations was low and could give rise to expanding outbreaks. The response to these outbreaks was not to implement a national vaccination campaign but rather a targeted approach, expanded when necessary. Although these data, based on the direct experience of infected populations, are not truly predictive for a modern smallpox outbreak (*1*), they are very instructive.

Analysis of the Edinburgh and Liverpool outbreaks suggests that outbreaks after deliberate release of smallpox virus may evolve over time. Therefore, sufficient opportunity exists for targeted enhanced surveillance measures to be put in place, for additional staff to be mobilized for an effective follow-up, and for a containment strategy to be implemented. The Liverpool outbreak took 15 months to control; the one in Edinburgh 3 months. This time difference probably reflects that reintroductions of smallpox occurred during the 1902–1903 outbreak because the disease was still endemic in the United Kingdom, poorer socioeconomic conditions existed in Liverpool at this time, and crowding was more prevalent, particularly in the dockland areas most heavily affected. By contrast in 1942, smallpox was no longer endemic in the United Kingdom, and socioeconomic conditions in Edinburgh were better. One might hope for at least as swift an end to a similarly sized modern-day outbreak as was seen in Edinburgh.

Modeling of data from other historical outbreaks of smallpox may help to further develop targeted surveillance and containment interventions for smallpox in the present era (*3**,**23*). Such interventions warrant further investigation because of clear, accumulating evidence of the substantial disease and death likely to accompany any mass population smallpox vaccination strategy.

## Appendix

This article was provided by The Archivist for the City of Edinburgh, Edinburgh, Scotland, United Kingdom.

Clark G, Seiter HE, Joe A, Gammie JL, Tait HP, Jack RP. The Edinburgh outbreak of smallpox, 1942. Authority of the Public Health Committee, Edinburgh, Scotland; 1944. Download PDF (3.72 MB, 92 pages).

## Supplementary Material

Technical Appendix The Edinburgh Outbreak of Small pox 1942.
