# Supplementary material for: Surveillance and Control Measures during Smallpox Outbreaks
Source: Emerg Infect Dis. 2005 Feb;11(2):291–7. doi: 10.3201/eid1102.040609 (PMC3320452; doi:10.3201/eid1102.040609)
Supplement: Technical Appendix — The Edinburgh Outbreak of Small pox 1942. [file 04-0609-app.pdf]

CITY OF EDINBURGH
PUBLIC HEALTH DEPARTMENT

THE

EDINBURGH OUTBREAK
OF
SMALLPOX
1942

Published by Authority of the Public Health Committee

CITY OF EDINBURGH

PUBLIC HEALTH DEPARTMENT

THE

EDINBURGH OUTBREAK

OF

SMALLPOX

1942

G. CLARK, M.B., F.R.C.P.(E.), D.P.H.

H. E. SEILER, M.D., D.P.H.

A. JOE, D.S.C., M.D., F.R.C.P.(E.), D.P.H., D.T.M. & H.

J. L. GAMMIE, M.B., D.P.H.

H. P. TAIT, M.D., F.R.C.P.(E.), D.P.H.

R. P. JACK, M.B., D.P.H.

Published by Authority of the Public Health Committee

INDEX OF CONTENTS

Page

Preface 3

Epidemiological Aspects :

History of the Outbreak 5
Discussion on Spread of Infection 11
Administrative Action 18

Clinical Account of Outbreak :

Age and Sex Distribution of the Cases 24
Initial Diagnosis 25
Clinical Types 26
Course, Convalescence, and Complications 28
State of Vaccination of the Patients 31
Diagnosis 33
Treatment 34

Vaccination and its Sequelae :

Technique of Vaccination 35
Analysis of Vaccination Figures 36
Vaccinial Reactions 39
Post-Vaccinal Encephalomyelitis; Incidence, analysis of cases, treatment, commentary 47

Disinfection of Property and Furnishings during Outbreak :

Disinfectant used 60
Procedure of Disinfection 61
Bacteriological Observations on Efficacy of Disinfection Measures 63

Bibliography 67

Appendices :

Appendix I. Table of Cases 70
Appendix II. Clinical Summary of Patients treated in City Hospital 72
Appendix III. Post-Vaccinal Encephalomyelitis—Case Histories and Pathological Data 83
Appendix IV. Map showing Location of primary City Cases in relation to the City Hospital . . . . . *Cover* p. iii

PREFACE

This Report is produced in its present form in the belief that it may be of some value to those who may be confronted with an outbreak of variola major.

When the outbreak occurred my staff was depleted by wartime demands, and few members had had any practical experience of dealing with the disease. Whatever credit there may be in the control of the outbreak, much of it belongs to the junior members who were facing a new experience.

As the Report shows, the occurrence of the disease was not unexpected, but the appearance of the first cases in a major voluntary hospital gave some weight to the hope that control would be easier than if they had occurred among the general population.

When the disease broke out in the Convalescent Home of the Royal Infirmary, it was felt that the mode of transference of the infection would be readily proved. By the time the first cases had occurred in the general population, the chapter of events was still unanswered, and, as the Report shows, no proof of the source of the infection was obtained in any of the group of eight primary city cases.

The Report is, in one respect, therefore, a record of disappointments. The means of introduction of the disease into Edinburgh, its spread from the Royal Infirmary to its Convalescent Home, and finally into the general population, were never proved. This, despite the fact that the most expert medical skill in the area centred round the primary institution.

The history of the outbreak, the administrative measures taken, clinical notes on the cases both of smallpox and of post-vaccinial conditions, and the method of disinfection carried out during the whole outbreak are given in some detail.

Since the various chapters of the Report do not lend themselves easily to summary form, it has been considered unwise to make any such attempt. There is one aspect of the outbreak and its consequences on which, however, I must comment.

This Report records ten deaths from vaccination and its consequences. These deaths occurred in well over 360,000 persons vaccinated in Edinburgh and adjacent counties during the Scottish outbreak. Eight of the deaths were attributable to encephalomyelitis following vaccination. The other two deaths occurred in children ; in one case the child, suffering from a skin complaint, was accidentally inoculated by a parent, while in the second case the child was vaccinated despite adverse medical advice.

On this matter, it must be emphasised that preventive action against the disease involving well over 360,000 persons resulted in the deaths of ten persons, whereas out of thirty-six cases of smallpox, eight died. Despite the risk attached to protective action against the disease, our knowledge of the high mortality from Asiatic smallpox in this country and the protection afforded by vaccination renders specific inoculation the sheet anchor in controlling the disease in the presence of an outbreak.

The administrator may find our experience of nursing cases of severe smallpox within the general fever hospital and close to a general hospital and housing scheme of some practical interest. The clinician will be more interested in

the description of the cases and of the somewhat high incidence of the hæmorrhagic type in our series, as well as in the rather limited experience of the use of sulphonamides in treatment.

The place of the hospital in the problem of aerial transmission of smallpox is discussed, although it was impossible to draw any definite conclusions.

It is a real privilege and pleasure to record my thanks to the members of the Public Health staff; to the staff of the City Hospital, many of whom sacrificed their freedom and leisure hours to nurse the cases ; to the doctors, nurses, and other personnel of the First Aid Posts, and to the City Architect and his staff, all of whom gave such loyal and cheerful support in a time of considerable stress.

My warmest thanks are also due to Colonel A. D. Stewart, Superintendent of the Royal Infirmary, whose energy and ripe experience were of the utmost value to me ; to the staff of that hospital and its convalescent home, especially to those connected with the affected wards, for their unstinted co-operation ; to Professor L. S. P. Davidson, Director of the Corporation's Medical Unit, Dr A. R. Gilchrist, an Honorary Physician to the Royal Infirmary, Professor Charles M'Neil and Dr L. H. F. Thatcher, Physicians to the Royal Hospital for Sick Children, Dr A. Murray Wood, Physician to the Children's Wing, Leith General Hospital, and Dr W. Blackwood, Neuropathologist to the Royal Infirmary, for so courteously granting me access to case records and pathological reports on those affected by the complications of vaccination ; and to Professor T. J. Mackie, Director of Bacteriological Services for the City, who supervised the bacteriological work carried out in connection with the work on disinfection.

I must also thank the various Medical Officers of Health in Scotland who furnished me with information, and in particular Dr A. D. Campbell, Medical Officer of Health for the County of East Lothian, and Dr John Riddell, Medical Officer of Health for the Counties of Midlothian, West Lothian, and Peebles, for their co-operation and willing assistance.

Finally, I should like to express my thanks to the members of the Public Health Committee for the freedom of action and support they gave me during a rather trying period, and for undertaking the publication of this Report.

W. G. CLARK, M.B., F.R.C.P.(E)., D.P.H.,
Medical Officer of Health.

Edinburgh, October 1944.

Edinburgh Outbreak of Smallpox 1942

EPIDEMIOLOGICAL ASPECTS

Up till the early years of the present century outbreaks of smallpox occurred in Edinburgh from time to time. In fact, until the year 1905 the City was seldom free from the disease, as will be seen from the following table (Table I.) which gives the incidence for the past fifty years. (Annual Reports of Medical Officer of Health, Edinburgh.)

TABLE I.

Year.	Total Cases.	Deaths.	Case Mortality.
1892	8	—	—
1893	51	1	1·9
1894	537	56	10·4
1895	109	16	14·6
1896	7	—	—
1900	5	—	—
1901	6	1	16·6
1902	7	—	—
1903	5	1	20·0
1904	168	15	8·9
1905	2	—	—
1908	20	—	—
1909	2	—	—
1920	9	—	—
1942	36	8	22·2

There was complete freedom from smallpox in Edinburgh from 1920 until 1942, when, during the months of October to December, a sharp outbreak took place affecting thirty-six persons.

History of the Outbreak.

General : Smallpox of a severe form was imported into Scotland on 29th May 1942, when a member of the crew of a ship arriving at Glasgow from the East was removed to hospital suffering from the disease (Macgregor and Peters, 1942). This first Glasgow case was the source of infection in ten persons, all of whom had been on board the ship, while subsequently, although no direct connection was ever clearly demonstrated, twenty-five members of the general population were affected. Some three weeks after the last Glasgow case had been isolated in hospital, smallpox broke out in a more or less circumscribed area in Fife County—twenty-nine persons developing the disease over a period of three months (Fyfe, 1943). As the Fife epidemic was drawing to a close the disease appeared in Edinburgh, at first in the Royal Infirmary, then in the Convalescent Home associated with that hospital, and later in the general community, a total of thirty-six persons being involved. The apparent

dates of the occurrence of the disease in each area are shown in the following statements :—

	Onset of Rash. First Case.	Onset of Rash. Last Case.	Admission to Hospital. First Case.	Admission to Hospital. Last Case.
Glasgow—				
(*a*) Ship infection .	24th May	13th June	29th May	13th June
(*b*) City infection . .	23rd June	31st July	26th June	31st July
Fife County	21st Aug.	13th Nov.	—	19th Nov.
Edinburgh—				
(*a*) Royal Infirmary infection . .	27th Oct.	12th Nov.	28th Oct.	12th Nov.
(*b*) Convalescent Home infection . .	4th Nov.	11th Nov.	5th Nov.	11th Nov.
(*c*) City infection .	18th Nov.	30th Dec.	18th Nov.	30th Dec.

Number of Cases according to Onset of Rash occurring each month in the different Areas during the year 1942

	May.	June.	July.	Aug.	Sept.	Oct.	Nov.	Dec.
Glasgow—								
(*a*) Ship infection .	1	10	—	—	—	—	—	—
(*b*) City infection .	—	17	8	—	—	—	—	—
Fife County . . .	—	—	—	1	2	24	2	—
Edinburgh—								
(*a*) Royal Infirmary infection .	—	—	—	—	—	2	7	—
(*b*) Convalescent Home infection . . .	—	—	—	—	—	—	14	—
(*c*) City infection .	—	—	—	—	—	—	7	6

It is impossible to regard this sequence of events except as a continuing infection, which, originating from the first Glasgow ship-borne case, was transferred from area to area by some means which careful investigation did not reveal. In fact, an outstanding feature of the Scottish smallpox outbreak of 1942 was the failure to find the connecting link between the occurrence of the disease in the various areas, and in the case of Glasgow and Edinburgh, between more or less closed communities and the general population.

Edinburgh Outbreak : A total of thirty-six persons were involved in the Edinburgh outbreak, of whom twenty-six were males and ten females. Relevant information regarding the patients is set out in Appendix I., and it will be observed that they fall naturally into two main groups, viz., (*a*) Institutional cases, *i.e.*, those infected in the Royal Infirmary of Edinburgh and its Convalescent Home, and (*b*) Cases in the general population.

Institutional Cases : The first case of smallpox was a man aged 46 years, who had been admitted to a surgical ward of the Royal Infirmary on 5th

October, for the treatment of a malignant condition of the face. He remained in this ward until 21st October, when he was transferred to the radium department for special treatment. He remained in this department until 26th October, when he returned to the original surgical ward. On 23rd October, eighteen days after admission, he developed a rise in temperature. He did not complain of headache, backache, or any other symptoms, and the view was taken that the rise in temperature was associated with his special surgical condition. The fever continued, however, and on 27th October, the day after his return from the radium department to the surgical ward, a generalised rash was noticed. He was transferred to the Edinburgh City Hospital as a case of chickenpox, where the diagnosis was tentatively confirmed as the distribution and general characteristics of the eruption conformed to chickenpox. At the same time, certain elements of the rash were not altogether typical of chickenpox, and as a precautionary measure the patient was placed under observation in accommodation earmarked for smallpox cases.

It should be mentioned that the patient had been successfully vaccinated in infancy and again during the Great War, 1914-18, and had good foveated scars on the right upper arm. Observation during the following few days showed that the disease was not developing in accordance with a typical attack of chickenpox, and on 1st November it was decided to treat the case as modified smallpox and take the necessary administrative action. Later in the same day on which this decision had been reached, it was learned that in the early hours of the same morning a boy had been admitted to the City Fever Hospital with a rather unusual rash. This boy, aged 13 years, had been sent to the hospital with a diagnosis of cerebro-spinal fever. On the afternoon of the day of his admission to the City Hospital, a maculo-papular eruption, irregular in distribution, was noticed on face, neck, and arms. Inquiry revealed that the boy had been from 15th to 29th October a patient in the same ward of the Infirmary as Case 1, and had sickened on the 29th October, the day of his discharge.

Neither of these cases when they first came to the notice of the Public Health Staff could, by itself, be diagnosed with any certainty as smallpox, but their association, along with the presence of smallpox in Scotland, reinforced the decision already taken in regard to the first case. A thorough investigation was conducted at the Royal Infirmary, but no evidence, conclusive or suggestive, as to the origin of infection of the two cases was forthcoming. A further point of interest was that both patients lived in the same street within a few yards of each other. A house-to-house visitation of that district was undertaken at once, but failed to reveal any clue to the source of infection. Nevertheless, in view of the apparent localisation of the two cases, and having in mind the probability of a missed case in the area, public vaccination in this district was started the following morning.

No further case of smallpox was reported until 5th November, when two men were discovered in an early stage of the disease in the large convalescent home of the Royal Infirmary, some three miles to the west of the parent institution.

On the evening of the same day, a Polish medical student, also in the early stages of the disease, was removed to hospital from a boarding house. No connection could be established between this student and the cases in the Infirmary or convalescent home. As he had been moving about in the City

during the prodromal period of the disease, it was decided to offer general vaccination throughout the City, and this was in full operation by the 8th November.

The two cases in the convalescent home had not been in the ward in the Infirmary from which the first two cases were removed, and the most careful scrutiny of movements between the Infirmary and the convalescent home failed to throw any light on the transfer of the infection.

The first of the patients in the convalescent home sickened on the 3rd November—the rash appearing on the 4th. He was admitted to the convalescent home on the 20th October from the parent hospital and, accepting the classical incubation period, it would appear that he received his infection shortly after admission to the home. The second case, whose rash appeared also on the 4th November, actually sickened that day, having been transferred to the convalescent home on the 23rd October, thus indicating the probability of having been infected in the parent institution. The Polish student was attending classes in the Infirmary and had been attending lectures in the group from which the second convalescent home case was transferred, although the student had never been in the ward in which the patient was accommodated. In fact he had come in contact with none of the patients in the Infirmary with the exception of some demonstrated in the Dermatology Department.

At this stage it became apparent that the infective agent was more widespread than had been hoped following the appearance of the first two cases. This was further emphasised by the discovery on the following day (6th November) of the disease in two other medical students (one a Pole) and another patient who had been discharged from the convalescent home nine days previously (28th October). On 5th November the convalescent home was placed in quarantine—all discharges and admissions of patients being discontinued. The Medical Officers of Health of the areas concerned were notified of all discharges from the convalescent home and from the affected wards of the Infirmary only, from the beginning of October.

On the 7th November four cases were removed from the convalescent home to the City Hospital, while other two patients who had been discharged from the convalescent home developed rashes—the first dying at home on the 8th November before he could be removed to hospital, while the second was removed to hospital on the 8th and died six days later (14th November). The last two cases were in East Lothian and Lanarkshire respectively.

The occurrence of these cases, all of whom developed a rash on the 7th November, indicated clearly a common source of infection in the convalescent home. Two of the patients had travelled in the same ambulance from the Infirmary to the home, and the whole method of transfer was again reconstructed and subjected to the closest scrutiny with completely negative results.

A further group of seven cases was transferred to the smallpox hospital within the next five days—two from the convalescent home, one from a nursing home, and the others from home addresses. The occurrence of this group pointed to a continuing infection in the convalescent home. Interspersed with these cases were other two—one the wife of Case 1, who had been kept under observation in the reception house, the other a patient in the ward in the Royal Infirmary originally infected, and whose illness could be associated definitely by contact with one of the original patients.

There were no further cases in the Royal Infirmary or the convalescent

home, and up till the 18th November no patient had been reported who had not been associated with one or other of these two institutions.

The total number of cases in this section of the outbreak was twenty-three, three of whom actually sickened in one ward of the Infirmary and were removed from there; one was the wife of the first case and she sickened when under observation in the reception house; one was a man who had been discharged from the Infirmary and sickened in a nursing home; while three were medical students attending classes in the Infirmary who were removed from their residences. Of the remaining fifteen, fourteen were infected while in the convalescent home; the locus of infection of the remaining case was doubtful, the classical period between exposure and rash suggesting that infection took place in the Infirmary.

City Cases:

So far, all the reported smallpox cases either had been patients in the Royal Infirmary or the convalescent home, or had been so closely associated with these institutions as to leave no reasonable doubt as to the place of infection. On 18th November, however, a young clerkess was found to be suffering from the disease. She had sickened on 16th November—the rash appearing two days later. Assuming a twelve-day incubation period, this girl had received her infection on 4th November. Despite the closest inquiry no relationship with the outbreak in either the Infirmary or its convalescent home could be discovered. This was the first case to occur in the general population, and subsequently twelve other cases contracted the disease. Among these thirteen cases, five were direct contacts with other city cases, the remaining eight being unassociated with each other or with the institutional outbreaks. The dates of sickening and of the appearance of the rash in these eight patients, with other relevant information, are given in Table II. (overleaf).

Table II. shows that seven of the patients sickened over a period of ten days, and that Cases 25, 26, 27, and 28 were affected about the same day, suggesting some common source of infection. Careful inquiry, however, failed to discover any connecting link between these cases. None of the patients had been associated with the affected institutions or with each other. There was no common link as regards work or home, and while, as the Table shows, three of the patients had visited places where numbers of people congregated, near the time of their infection, none of these places was common to all.

The last primary case to be discovered was a man, aged 55 years, who died on the third day of his illness from a fulminating attack of the disease. No case had occurred in the city for a period of thirteen days between the date of his sickening and the removal of the last primary city case to hospital. No definite source of infection was ascertained in his case, but he resided near a group of three cases—one primary and two secondary—and there is the possibility that he might have been infected from this source although contact was never proved.

Altogether five secondary infections, all in home contacts, occurred in connection with the city outbreak. Three of these cases sickened in and were removed from the reception house. The remaining two were the mother and father of Case 25—a woman of 24 years. This woman had been vaccinated

TABLE II.

No.	Patient	Sex	Age	Occupation	Date of Sickening	Date of Rash	Probable dates of infection*	Significant movements *circa* possible date of infection	Date of Vaccination	Type of Disease
24	A.F.	F.	17	Clerkess	16th Nov.	18th Nov.	4th Nov.	Picture House, 6th Nov.	14th Nov.	Modified discrete.
25	A.C.	F.	24	Machinist	19th Nov.	22nd Nov.	8th Nov.	Dance Hall 6th and 7th Nov.	10th Nov.	Modified sparse.
26	H.C.	M.	28	Telephone Engineer	19th Nov.	23rd Nov.	7–9th Nov.	Picture House 11th and 12th Nov.	15th Nov.	Modified discrete.
27	E.S.	F.	18	Bakery Worker	20th Nov.	23rd Nov.	8–9th Nov.	Dance Hall, 7th Nov. Cinema, 8th and 9th Nov.	14th Nov.	Modified discrete.
28	C.H.	F.	45	Clerkess	20th Nov.	23rd Nov.	8–9th Nov.	—	—	Modified discrete.
29	W.G.	M.	35	Chemical Process Worker	22nd Nov.	26th Nov.	11–12th Nov.	Cinema, 10th Nov.	17th Nov.	Modified discrete.
30	J.S.	F.	58	House-keeper	26th Nov.	29th Nov.	14–15th Nov.	Public Library, 14th Nov.	—	Hæmorrhagic.
35	M.B.	M.	55	Carter	13th Dec.	15th Dec.	1st Dec.	Cinema, 30th Nov.	—	Hæmorrhagic.

* Calculated 12 days from sickening date and 14 days from rash.

successfully on 10th November. Some twelve days later, spots were noticed on the face. These were considered by her doctor to be due to vaccinia. The girl, however, was kept off work and for the most part remained in the house until the 7th December, when the nature of her illness was discovered by the recognition of hæmorrhagic smallpox in her mother.

Discussion on Spread of Infection :

Table III. (overleaf) sets out the approximate dates of infection and the appearance of the rash in those patients in whom no definite source of infection could be traced. Cases of secondary infection have been omitted.

The three main periods of infection of the patients are :—(*a*) that in the Royal Infirmary from 13th to 29th October, (*b*) the convalescent home outbreak from the 21st to 28th October, and (*c*) the city infections extending from 4th November to 1st December.

Most of the patients fall definitely into one or other of these groups, but there is difficulty in allocating four of the institutional cases (Cases 5, 7, 10, and 23) as shown below :—

	Royal Infirmary.			Convalescent Home.				Probable date of infection from date of	
		Date of		Date of		Date of			
Case.	Ward.	Admisn.	Disch.	Admisn.	Disch.	Sickng.	Rash.	Sickng.	Rash.
No. 5	12	9/x	20/x	20/x	5/xi	3/xi	4/xi	22/x	21/x
No. 7	7	19/x	23/x	23/x	5/xi	4/xi	5/xi	23/x	22/x
No. 10	13	18/x	23/x	23/x	7/xi	4/xi	7/xi	23/x	23/x
No. 23	7	27/x	4/xi	16/x	27/x	10/xi	12/xi	29/x	29/x

If the period of fourteen days from the appearance of the rash is used in calculating the probable time of infection, Cases 5 and 10 were infected in the convalescent home the day after transfer from the Royal Infirmary ; Case 7 was infected in the Royal Infirmary the day before transfer to the convalescent home ; Case 23 was infected in the Royal Infirmary two days after being transferred back to that hospital from the convalescent home. On the other hand, if it is calculated that infection took place twelve days from the date of sickening, the position of Case 23 is unaltered, but Case 5 was infected in the convalescent home two days after admission, while Cases 7 and 10 were infected on the day of transfer from the one institution to the other. It is recognised that the incubation period may vary from the classical period of twelve days. The question of allocation is of importance, because if all four cases be regarded as Royal Infirmary infections, then three wards in that institution were involved in the outbreak, while if they were convalescent home infections only one ward in the Infirmary was affected.

It is, of course, impossible to answer this question with any degree of certainty, but it is considered from the available evidence that it is unlikely that Cases 5 and 10 were infected in the Royal Infirmary. The dates of sickening and appearance of the rash in Case 5 were more in keeping with a convalescent home infection. Case 10 also is more likely a convalescent home infection when viewed in relationship with other patients who were without

TABLE III.

Showing probable dates of infection and date of appearance of the rash in the cases in which no definite source of infection could be discovered.

	October.												
	13	17	20	21	22	23	24	25	26	27	28	29	31
Institutional Cases :—													
(*a*) Probable date of Infection.													
R.I.E.—													
Ward 13	1	1	–	–	–	–	–	–	–	–	–	–	–
Medical Students	–	–	2	–	1	–	–	–	–	–	–	–	–
Ward 7	–	–	–	–	1	–	–	–	–	–	–	1	–
Convalescent Home	–	–	–	1	–	1	7	1	1	2	1	–	–
(*b*) Date of Rash.													
R.I.E.—													
Ward 13	–	–	–	–	–	–	–	–	–	1	–	–	1
Medical Students	–	–	–	–	–	–	–	–	–	–	–	–	–
Ward 7	–	–	–	–	–	–	–	–	–	–	–	–	–
Convalescent Home	–	–	–	–	–	–	–	–	–	–	–	–	–
City Cases :—													
(*a*) Probable date of Infection	–	–	–	–	–	–	–	–	–	–	–	–	–
(*b*) Date of Rash	–	–	–	–	–	–	–	–	–	–	–	–	–

	November.																December.	
	3	4	5	6	7	8	9	10	11	12	15	18	22	23	26	29	1	15
Institutional Cases :—																		
(*a*) Probable date of Infection.																		
R.I.E.—																		
Ward 13	–	–	–	–	–	–	–	–	–	–	–	–	–	–	–	–	–	–
Medical Students	–	–	–	–	–	–	–	–	–	–	–	–	–	–	–	–	–	–
Ward 7	–	–	–	–	–	–	–	–	–	–	–	–	–	–	–	–	–	–
Convalescent Home	–	–	–	–	–	–	–	–	–	–	–	–	–	–	–	–	–	–
(*b*) Date of Rash.																		
R.I.E.—																		
Ward 13	–	–	–	–	–	–	–	–	–	–	–	–	–	–	–	–	–	–
Medical Students	2	–	1	–	–	–	–	–	–	–	–	–	–	–	–	–	–	–
Ward 7	–	–	1	–	–	–	–	–	–	1	–	–	–	–	–	–	–	–
Convalescent Home	–	1	–	1	7	1	1	2	1	–	–	–	–	–	–	–	–	–
City Cases :—																		
(*a*) Probable date of Infection	–	1	–	–	–	1	3	–	–	1	1	–	–	–	–	–	1	–
(*b*) Date of Rash	–	–	–	–	–	–	–	–	–	–	–	1	1	3	1	1	–	1

question infected in that institution. The facts according to this line of reasoning may be stated as follows :—

There were seven patients, including Case 10, in whom the rash appeared on the same day. The particulars of these cases are :—

	Convalescent Home. Date of		Date of		Minimum incubation period if Royal Infirmary infection.
Case.	Admisn.	Disch.	Sickng.	Rash.	
9	9/x	7/xi	4/xi	7/xi	26 days
10	23/x	7/xi	4/xi	7/xi	12 ,,
11	14/x	29/x	4/xi	7/xi	21 ,,
12	16/x	7/xi	5/xi	7/xi	20 ,,
13	20/x	7/xi	5/xi	7/xi	16 ,,
14	13/x	4/xi	5/xi	7/xi	23 ,,
15	2/x	31/x	5/xi	7/xi	34 ,,

Even allowing for considerable latitude in the incubation period, most of these cases must have received their infection in the convalescent home, and it is fair to assume that all, including Case 10, fall into this category.

While from the foregoing discussion Cases 5 and 10 may be regarded as convalescent home cases, the question of the proper allocation of Cases 7 and 23 is more difficult. Case 7 was transferred from the Royal Infirmary on the same day (23rd October) as Case 10, and may have received his infection from the same undiscovered source. On the other hand, he was a patient in the same ward (ward 7) in the Royal Infirmary as Case 23 who, unless the incubation period was delayed for fourteen days or longer, was infected in the Royal Infirmary. A further point is that the particular ward (ward 7) from which Cases 10 and 23 came was associated with the two Polish medical students (Cases 3 and 6) who, while they did not actually see cases in the ward, attended clinical lectures in the ward sideroom. On the other hand, the third medical student (Case 4), whose sickening date was intermediate between the two Polish students, had no association with ward 7.

Having regard to all the circumstances, therefore, it has been decided to place Cases 5 and 10 in the convalescent home group and to consider Cases 7 and 23 Royal Infirmary infections, although it is recognised that they could be allocated otherwise.

Royal Infirmary Infection : Nine cases, eight males and one female, are regarded as having been infected in the Royal Infirmary. Of these, five were patients, three were medical students, and one was the wife of the first case. The dates of sickening of the first two patients and of the three medical students are spread over a period of ten days and are suggestive of a continuing infection in the institution, as it is unlikely that any of these cases were infected from one another. Of the other four Royal Infirmary cases, two (Cases 21 and 22) in all probability received their infection from known cases in the ward first affected, while the remaining two (Cases 7 and 23) may have been infected from the same source.

It may be said at once that the inquiries made failed to ascertain how the infection was introduced into the Royal Infirmary and how it spread in the institution. All the patients in the institution, and recent ex-patients in the affected wards, were carefully examined and their records scrutinised. The medical, nursing, technical, domestic, clerical, and artisan staffs, and all students attending the hospital, were included in this comprehensive examination. An attempt was made by press notice, wireless broadcasts, and inquiry

from the patients to trace and examine visitors who might have introduced the infection, and also those most likely to have been exposed to infection. There was, of course, no means of checking visitors accurately and all may not have come forward, but the response of relatives and friends was regarded as very satisfactory. The possibility of the transference of the infection within the institution by instruments, equipment, fomites, etc., was carefully investigated but proved negative. No clue was obtained by minute inquiry into the movements and activities of the three infected medical students whose contact with the hospital was comparatively slight. Two attended a few lectures in a side room in one of the affected wards, but in no instance did they come into contact with any of the patients in that ward. The third student attended classes in the Dermatology Department, and this fact led to careful re-assessment by the dermatologists of their cases during the previous few weeks, but without demonstrating any missed case.

The history of one patient who died in Ward 13 of the Royal Infirmary where the first two cases of smallpox occurred, was considered in some detail.

Case History: A patient, male, aged 21 years, was admitted to Ward 13 of the Infirmary on 8th October (*i.e.*, fifteen days before the first case of smallpox sickened), and he died the following day.

Previous History: This was difficult to obtain because the patient was extremely ill, but apparently he had had gastric trouble for the previous thirteen weeks and for the last five weeks prior to admission he had been forced to stay off work on account of severe headaches. On 5th October he had pain in both kidney areas with vomiting and oliguria. On admission, the patient was extremely ill, very distressed, and somewhat confused. He complained of pain in the back, and the abdomen was very rigid. During the time between his admission and death, he coughed up some blood. The temperature varied between 98° and 100° F. The following day he died, following a profuse rectal hæmorrhage.

A blood examination carried out on his admission revealed a leucocytosis, the differential count showing a preponderance of lymphocytes.

Autopsy: This showed petechial hæmorrhages and ecchymoses in most of the thoracic and abdominal viscera, and also in the spinal cord where they appeared to be subdural in position. The only obvious enlargement of the lymphatic glands was in the posterior triangle of the neck on the left side. Spleen was enlarged, firm in consistence, appeared slightly thickened, and the cut surface was pink in colour and jelly-like in consistence.

Histological Examination: (*a*) Lymph gland (removed from hilum of lung)—"Germinal centres were prominent and are packed with mononuclear cells of apparently primitive type." (*b*) Spleen—"Malpighian corpuscles are distinct and are packed with cells with markedly hyperchromatic nuclei, splenic pulp is hæmorrhagic." (*c*) Liver—"Shows an even distribution of leukæmic cells between portal tracts and the liver lobules. The organ shows cloudy swelling and fatty

degeneration." (*d*) Intestinal mucosa—" Infiltrated with leukæmic cells." (*e*) Heart and Kidney—" Both showed degenerative changes with irregular groups of primitive mononuclear cells." (*f*) Brain—" Many primitive white cells are seen in the dilated cerebral capillaries, but none are seen to be lying free of the cerebral substance." (*g*) Spinal Cord—" Small amount of free blood was present in the anterior median fissure and hæmorrhage was also present in the dura. The cord, otherwise, showed nothing abnormal."

The cause of death was certified as " Acute Lymphatic Leukæmia." In the light of subsequent events, however, the facts of this case were submitted to the medical authorities of the Infirmary, but after careful consideration of the case, they expressed themselves as satisfied with the original diagnosis of acute lymphatic leukæmia.

Convalescent Home Infection : Fourteen patients, all males, are included in this portion of the outbreak—the probable period of infection extending from 21st to 28th October—dates of extreme importance as the infective period in the convalescent home corresponds very closely with the time that Case 1, the first smallpox patient, remained in the Royal Infirmary. This patient (Case 1) sickened on 23rd October, but was not removed to the isolation hospital until the 28th October. This fact cannot be dissociated from the convalescent home outbreak, but its precise relationship is a matter of conjecture. Many of the patients in the convalescent home who sickened with smallpox had been inmates of the home for periods sufficient to exclude any other locus of infection. At the same time, it is true that a more or less constant transference of cases to and from the convalescent home and the Royal Infirmary took place, and it is quite possible that infection was conveyed in this way. Careful examination and surveillance of all patients in the home and of all who had been discharged within the previous month proved negative, and although the method of transference between the two institutions was carefully reconstructed, no clue as to the infective source was obtained.

Another point of epidemiological interest is that the convalescent home is divided into three wards : two for males and one for females, the latter being on a different floor. Many of the patients in these three wards were up and about and walked in the grounds of the institution. Thirteen of the patients came from one male ward, the fourteenth case being a young boy from the other male ward who spent most of the day in the ward where all the other cases were infected. This boy sickened five days after his discharge home. While the three wards had each their own nursing staff, the nurses mixed freely in the institution apart from their ward duties. Thus localisation in one ward of a heavy infection cannot be explained. Five of the thirteen cases sickened in the ward and were therefore potential sources of infection despite the fact that they were removed to the smallpox hospital forthwith.

City Infection : There were thirteen cases in this group, five of them definitely associated with previous known cases. The source of infection in the remaining eight was never discovered. Seven of these cases were probably infected during a period extending from 4th to 15th November, while in the eighth the probable date of infection was the 1st December. This latter case may have contracted the disease from another patient who lived in the same

locality, but no evidence of contact was ever obtained. Four of these primary cases were probably infected on the 8th–9th November, suggesting a common source, but the closest investigation failed to reveal any common factor. None were known to any other. Their habits were different ; their homes and social strata varied, while in two their movements at this time were very restricted as is shown in the following account.

Case 26 had arrived in Edinburgh on holiday on 7th November, twelve days before sickening with smallpox. He had remained indoors at home from the date of arrival until 9th November. This is the period when, according to usual calculations, he received his infection, and in his particular case the history of onset of the disease and the occurrence of the rash were quite definite. The only movements of interest while in Edinburgh were visits to cinemas nine and seven days before sickening, shopping in the centre of the City eight days before sickening, and attendance at a First Aid Post for vaccination five days before sickening.

The movements of Case 28 were even more restricted. The patient, a woman aged 45 years, had been off work through illness for seventeen days prior to developing smallpox. During that time, she had had very little communication with the outside world. She lived alone, and, apart from short walks into the neighbouring countryside, had remained for the most part indoors. She attended no places of public entertainment, and on one occasion only, some three or four weeks before sickening, visited the centre of the city. In fact, it would appear that the only method whereby she could have obtained her infection was during shopping activities, and in her case this was undertaken to a very limited extent, most articles, such as food, being sent in by retailers.

It will be observed from Table III. that the apparently primary city cases were infected during the time when the eruption of the disease was appearing in the convalescent home patients. This suggests a connection between the two groups. In spite of the closest inquiry, all the city patients denied any association, even remote, with either the Royal Infirmary or its convalescent home, and, as has been said above, no missed case from either of these institutions was discovered.

The smallpox outbreak in Edinburgh from the epidemiological standpoint was unsatisfactory in that the spread of the disease from institution to institution, and from institution to general population, was never explained. This, however, is in keeping with the general history of the outbreak in Scotland. Thus, the method of spread of the disease from ship to general population in Glasgow, from Glasgow to Fife, and from Fife to Edinburgh, was never demonstrated. The most obvious explanation of this series of events is to postulate the occurrence of "missed" cases of the disease or to place on infected fomites the responsibility for the spread. In so far as Edinburgh cases are concerned, it is difficult to reconcile the former theory with the actual facts. The disease at the outset was confined to a more or less closed institutional community. All known possible contacts were carefully examined and observed and it can be said with some assurance that no case of the usual modified type of the disease escaped notice.

Do mild attacks of the disease without the appearance of a rash occur, and if so, are they infectious? Again, it may be asked if the spread of smallpox can be attributable to "carriers." The "carrier" method of spread of infectious disease has been established for many of the bacteria-caused diseases,

and there is evidence that in some virus diseases, *e.g.*, poliomyelitis (Top and Vaughan, 1941 ; Piszczek *et al*, 1941) and influenza (Pettit *et al*, 1936 ; Francis *et al*, 1937), a similar "carrier" state may occur. Carriers would explain the passage of smallpox from the Royal Infirmary to its convalescent home, but it is difficult to reconcile this theory with the occurrence of the disease in the general population. It would be necessary to postulate a high carrier rate in the general population to account for the scattered distribution of the city cases. Infected fomites might explain the passage of the disease from institution to institution, although this obvious possibility was carefully investigated with negative results. It would not explain the occurrence of the disease in the general population. As has been mentioned previously, none of the eight "primary" cases in this group had any association whatever with the Royal Infirmary, the convalescent home, or with each other. Their activities and interests differed widely, and in spite of careful inquiry no common factor was ever discovered.

The question of aerial convection of infection in relation to the smallpox hospital remains to be considered. The smallpox hospital is situated to the south-west of the city and the prevailing wind is south-west. At no time was there any aggregation of patients in the infective stage in either the Royal Infirmary or the convalescent home. The first case of smallpox was admitted to the City Hospital on the 28th October and the last case was dismissed on the 27th March—the largest number of cases in the hospital at one time being 22 patients on the 26th November. The geographical position of the City Fever Hospital, and the location to the hospital of the eight primary cases among the general population, is shown on the map in the Appendix, while the following table gives the probable dates of infection and other relevant information regarding these cases.

Case No.	Date of Rash	Probable date of Infection	Localisation of Home from City Hospital		Number of Cases of Smallpox in City Hospital on probable Date of Infection of Cases
			Direction	Distance	
24	18/xi	4/xi	N.	1½ miles	2 (no case crusting)
25	22/xi	8/xi	N.E.	3½ ,,	12 (1 case crusting)
26	23/xi	9/xi	N.E.	2¾ ,,	12 ,, ,,
27	23/xi	9/xi	W.N.W.	1¾ ,,	12 ,, ,,
28	23/xi	9/xi	N.E.	¾ mile	12 ,, ,,
29	26/xi	12/xi	N.E.	4¾ miles	20 (1 crusting)
30	29/xi	15/xi	N.E.	1 mile	20 (5 crusting)
35	15/xii	1/xii	N.E.	3½ miles	22 (all crusting)

Seven of the patients resided to the north or north-east of the hospital at distances from just under one mile to approximately four and three-quarter miles—the remaining patient living approximately one and three-quarter miles to the west-north-west of the hospital. It may be explained that the activities

of the patients so far as could be ascertained did not bring them into any closer proximity to the hospital.

Information kindly provided by the Royal Observatory, Edinburgh, showed that during the period of prevalence of smallpox in Edinburgh, that is to say from November to January inclusive, the prevailing wind was from the south-west. In actual figures out of 184 observations the wind was from south to west in 112 instances; from south-south-east to east in 33; from north to west-north-west in 18; while in the remaining 21 observations there was no wind. During the first three weeks of November, the period during which seven of the city cases were infected, the wind was predominantly south-west in direction, veering on one occasion only beyond the west.

It should be noted that at the time when the first city case was infected there were no patients in the City Hospital in the crusting stage of smallpox. At the time when the next five city cases were infected there was only one patient in the crusting stage. When the seventh case was infected there were five patients in the crusting stage in the hospital, while when the last case received his infection all the cases in the smallpox hospital were in the crusting stage.

It should be mentioned that a large general hospital lies within a quarter of a mile to the north-east of the City Hospital and a housing scheme of approximately 500 houses is situated directly to the east, all the houses lying within one and a half to three-quarters of a mile. No cases of smallpox occurred either in this scheme or in the general hospital.

No definite conclusions can be drawn from the limited experience of this outbreak, but 33 cases of smallpox were treated during a period of three months in close proximity to large groups of people living within three-quarters of a mile and in the direction of the prevailing wind and no case of smallpox developed amongst them. A further point of importance which cannot be separated from this question is the fact, as is shown later (page 23), that smallpox patients were nursed in close proximity to other infectious diseases cases. The great majority of these were protected by vaccination and revaccination, but a few cases, because of the nature of their illness, remained unprotected in this way for varying periods and yet no infection took place.

This epidemic of smallpox in its early stages appeared to contain all the elements which would lead to a conclusive finding as to its introduction and spread, but, as it progressed, the problems became more difficult to elucidate, and, finally, no satisfactory explanation could be given as to the source of infection in any of the primary cases.

It should be emphasised that the occurrence of smallpox in institutions such as the Royal Infirmary and the convalescent home, with a medical and nursing staff experienced and highly trained in clinical observation, and the whole-hearted co-operation of the administrative staff of these institutions with the Public Health officials, ensured a higher degree of supervision of the inmates and staff than is possible in the circumstances usually surrounding epidemics of the disease.

Administrative Action :

The administrative action taken in Edinburgh during the smallpox outbreak in Scotland was in conformity in all essential respects with well recognised means of combating the infection, but for completeness may be considered under three headings, viz., (*a*) measures taken during the Glasgow

outbreak of June-July 1942; (*b*) measures taken during the outbreak in Edinburgh, and (*c*) isolation hospital measures.

(*a*) **During Glasgow Outbreak:** Certain general precautionary measures were taken in Edinburgh during the outbreak of smallpox in Glasgow. Attention was particularly directed to the protection of essential personnel, and vaccination and re-vaccination facilities were extended to members of all municipal departments, including hospital staffs, Civil Defence workers, and Police. The great majority of persons in these services readily availed themselves of the opportunity. Supplies of vaccine lymph were issued to general practitioners who wished to protect their patients, while members of the general public who desired were vaccinated at the Public Health Department, or at their place of work at the request of their employers. In all, some 20,000 vaccinations were carried out during this period.

According to routine practice, all known or suspected contacts of Glasgow cases arriving in or passing through Edinburgh were examined and kept under daily surveillance until the expiry of their quarantine periods.

(*b*) **During Edinburgh Outbreak:**

(1) *Institutional Infection:* It has already been pointed out that the first two cases of smallpox in Edinburgh occurred in the same surgical ward of the Royal Infirmary and that they resided in the same street of the city, a few doors from one another. Keeping in mind a possible common source of infection having been introduced into the hospital from the neighbourhood, a house-to-house visitation of this street was carried out on the evening of 1st November, the day on which Case 1 was finally diagnosed as smallpox. This home investigation produced no evidence of any local source of infection.

While this inquiry was being carried out, investigations were being conducted in the Royal Infirmary. There, each honorary surgeon is in charge of two wards—one for males and one for females—both wards being served by the same theatre staff, nursing and domestic staffs, etc. All male and female patients, together with the medical, nursing, and domestic staffs, and all students of both wards of the unit wherein the first case occurred were subjected to a careful examination and offered vaccination. Clinical findings were again negative. Both wards were placed in strict quarantine and daily surveillance of patients, staff, and students carried out. Patients recently discharged from the affected wards were followed up. All consented to vaccination or revaccination and were kept under observation for eighteen days. Similarly all known visitors to the two wards during the three weeks prior to the occurrence of the first two cases of smallpox were examined in their homes and offered vaccination.

The occurrence of smallpox in the three medical students necessitated the extension of the investigation to every person in the Royal Infirmary. The help and co-operation of the University and Surgeons' Hall authorities allowed the examination of all medical and dental students to be rapidly overtaken, but again with negative results. To exclude the possibility of the spread of infection by fomites, all details of the hospital administrative service were minutely scrutinised. Such possibilities as emerged from this investigation were carefully followed up without affording any solution.

The Dermatology Department was an obvious place to which to direct the closest attention—quite apart from the fact that one of the students was

attending classes there—and the dermatologists co-operated whole-heartedly in a reconsideration of the cases attending during the previous few weeks. No missed cases came to light as a result of this review. A similar procedure was adopted in the case of the convalescent home with, in addition, detailed reconstruction of investigations into the whole method and procedure of transfer and transport of patients from the Royal Infirmary to the convalescent home and *vice versa*.

The severity of the infection among the patients, both in the hospital and the convalescent-home, and the widespread movements of the medical students in the hospital, necessitated a consultation with the Honorary Medical Committee to determine what steps should be taken regarding the continued activities of the hospital. It was decided at this meeting, with subsequent approval by the Board of Managers, to admit to the hospital only those cases who showed evidence of recent successful vaccination, or, in the case of the acutely ill, to admit with a view to vaccination as soon as the staff deemed it advisable. All visitors to the hospital were prohibited except under special permission. Notices to these effects were displayed prominently in the hospital precincts and appeared in the public press.

(2) *Infection among the General Population:* There were thirteen cases of smallpox in the general population—the first case being diagnosed on 18th November and the last on 30th December 1942. Of these, one patient sickened two days after proceeding from Edinburgh on holiday, while three were removed from the reception house where they had been under close observation. The occurrence of the remaining ten cases, however, necessitated careful and detailed reconstruction of their movements from the time of sickening until removal to the isolation hospital, and entailed in a number of instances the tracing and supervision of a large number of contacts.

Three cases, Nos. 29, 32, and 35 in particular, had in the course of their employment been associated with large numbers of people and they together necessitated the examination, supervision, vaccination, or revaccination of more than 900 persons. Case 35 was employed as a carter and had visited in the course of his duties, and after the rash had appeared, many important firms in the city, in addition to mixing freely with his fellow-workers. There were 367 known contacts associated with his illness. Case 24, a young clerkess, had visited on the first day of sickening a bank and several Government Departments in addition to travelling extensively by tramcar. There were 118 known contacts in her case, all of whom were kept under observation for eighteen days.

At least four of the patients for varying periods after sickening had used public conveyances, such as tramcars, buses, and trains, and had attended places of public entertainment. In an endeavour to trace contacts notices were inserted in the public press giving a detailed account of the movements of these patients and urging all who might have been in contact to report at the Public Health Chambers. As a result of these notices, many contacts were examined and kept under supervision.

Some 1700 members of the public, in addition to staffs and students associated with the Royal Infirmary, were dealt with as contacts, and were visited and kept under close observation for eighteen to twenty-one days. Most of them readily agreed to vaccination or revaccination,

(3) *Reception House:* In view of previous experience of smallpox outbreaks, it was decided to keep the family contacts of cases under the closest supervision, and for this purpose a children's home was rapidly prepared for use as a reception house and was opened on the day following confirmation of the diagnosis of the first two cases. It was staffed by specially experienced personnel. In all 27 persons were admitted to the reception house for observation during the course of the outbreak. The number dealt with was small, but, in fact, the Royal Infirmary convalescent home, where a large number of cases occurred, acted as an observation ward, and relieved the pressure on the reception house. The inmates of the reception house were kept in quarantine for twenty-one days—temperatures being taken night and morning, and clinical examination carried out at the same time. No restrictions were made regarding work, but in practice, employers, in all instances except one, were prepared to pay wages during the quarantine period rather than cause anxiety to fellow-workers. The exception attended her work during the day and returned to the reception house at night. She was carefully examined before leaving and on returning, and at no time showed evidence of infection.

Three cases of smallpox—the wives of two patients and the ten-months' old daughter of another—occurred among the twenty-seven persons kept under observation in the reception house. In each instance, the disease was of a modified variety. In addition, two other contacts who developed temperatures while in the reception house were transferred immediately to the infectious diseases hospital for observation, but in neither case did a rash develop.

The reception house was also used to house four patients after their recovery from smallpox to permit of further disinfection of their houses. Owing to the fact that these patients had been the only occupants, the houses had been closed since the initial disinfection following their removal to hospital and it was necessary to find some accommodation for them while the houses were being prepared for re-occupation.

(4) *General Vaccination:* It has been previously pointed out that the first two cases of smallpox in the Royal Infirmary resided in close proximity to each other. The First Aid Post in the neighbourhood was immediately opened as a vaccination centre with sessions held thrice daily. Some 8000 persons took advantage of the facilities and were vaccinated or revaccinated.

The occurrence of smallpox in the first two medical students raised at once the possibility of the exposure of a large number of citizens to the disease and pointed to the necessity for offering protection to the general population. Twenty-two vaccination centres were opened throughout the city on 8th November, and they continued to function until 8th December. With the subsequent occurrence of three cases in one family in a central district of the city, the First Aid Post serving that area was reopened from 9th December to 12th December and again from 21st December to 24th December, in view of the late appearance of Case 35 from the same district.

(*c*) **Isolation Hospital Measures :** We had information of the discovery of the first Glasgow case on 29th May and steps were taken the same evening to commence vaccination of all members of the medical, nursing, domestic, artisan, and ambulance staffs. Vaccination or revaccination is a condition of service for all City Hospital nurses, but no risks were taken and all members

of the staff were revaccinated, no person being accepted as immune unless the operation failed on three separate occasions. Unhampered at this time by the official pronouncements which were made later, vaccination consisted of four single linear insertions, 1¼ inches apart. When the diagnosis of the first two Edinburgh cases was confirmed on 1st November, vaccination with the specially recommended one linear insertion of all patients in the hospital was commenced.

It was ascertained that over one hundred unvaccinated children under five years were patients and the limited supply of lymph available was reserved for them and the ward contacts. Parental consent was obtained by giving full lists of addresses to the City Police, who promptly visited the addresses, with the result that within two hours relatives began to arrive at the hospital to sign the consent forms. We cannot be too grateful to the Police Authority for this extraordinary prompt and effective assistance. Approximately eighty children were vaccinated the same evening, as well as a considerable number of patients who were of the age of consent. In the course of a few days all patients were vaccinated, and as long as there were smallpox patients in the hospital, vaccination of all admissions, or as soon after as circumstances permitted, was routine, the procedure being repeated twice if necessary. When carrying out removals ambulance nurses took consent forms with them, and practically invariably they were signed. Over the whole period, refusals amounted to less than one-half per cent., and no patient complained of more than temporary discomfort.

Accommodation : At the commencement of the Glasgow outbreak, steps were taken to recondition the fabric of the smallpox hospital, a temporary building containing twenty-four beds with quarters for eight nurses. In addition, small isolation units or " cottages " in the isolation hospital grounds proper, each containing two three-bedded wards, were put in readiness to receive observation cases. Eight of these were constructed in the original plan of the hospital as isolation accommodation, but they have been little used as they are uneconomic from the standpoint of staffing. In this smallpox outbreak they were to prove invaluable. Temporary buildings were rapidly erected at the smallpox hospital by the City Architect's staff, one consisting of two bedrooms and bathroom for male staff, and another comprising a discharge unit, with mortuary and post-mortem room. Food was conveyed from the hospital main kitchen and emptied into receptacles placed on a table a short distance from the smallpox wards. Laundry was washed out in the smallpox hospital wash-house, then placed in a bin containing carbolic solution for twelve hours. From this bin it was then conveyed to the main hospital laundry.

Soon after the epidemic commenced it became clear that patients would be scattered over the South-eastern Counties of Scotland where facilities for the reception of smallpox patients were scanty or non-existent. It was decided, therefore, to afford all facilities available in the City Hospital to such patients to the limit of our accommodation. At one stage in the outbreak, when there were nineteen patients in the smallpox hospital, arrangements were made with Midlothian County for the use of one of their institutions for the reception of cases, but fortunately this did not require to be utilised.

In the early stages of the outbreak, the patients were all males and no problem arose as to the provision of separate accommodation for the sexes.

By the time females began to be admitted the number of male cases was such that it was not possible to secure reasonable separation of the sexes in the smallpox hospital. It was therefore decided to set apart one of the " cottages " in the Fever Hospital for female patients, although this was situated at a distance of no more than 85 feet from a fully occupied two-storey diphtheria pavilion. Staff accommodation for these cases was provided in a neighbouring cottage, and food supply and laundry procedures were as for the smallpox hospital.

Of recent years a certain amount of discussion has centred on whether the provision of separate smallpox hospitals are really necessary, and this limited experiment would indicate what can be achieved within the boundary of a fever hospital. It should be realised, however, that the number of smallpox patients accommodated at any one time was small, the highest number being six, the specific immunity against smallpox was kept at a very high level in the patients and staff of the City Hospital, while the staff selected for smallpox nursing was of the highest quality and by the time this experiment was started they had acquired a thorough experience of the routine. Whether the experiment would have proved satisfactory with much larger numbers of patients or a less reliable staff may be questioned, but no secondary case did occur in the fever hospital.

The experiment does show, however, that the isolation of a few cases of smallpox can be carried out in a general fever hospital and the opening of the special smallpox hospital with all its attendant expense need not necessarily be resorted to. Our female patients certainly derived great advantages from being housed in the " cottage." These are modern buildings of first-class construction with every facility in respect of lighting, heating, hot-water supply, and ventilation. Smallpox is a serious disease and for its proper treatment requires every facility that modern hospital construction can provide. This has not always been realised in the past. Smallpox being infrequent in this country, smallpox hospitals are often temporary structures and sometimes placed in isolated positions and patients have suffered unnecessarily.

Observation accommodation for suspected cases was much in demand during the outbreak. Usually it was possible to determine the nature of the case in the course of two to four days, but during this time the patient was a potential case of smallpox and all precautions as for a case of that disease required to be taken. Here, again, the " cottages " proved their worth, the main difficulty experienced at times being to provide adequate staff, each observation case often requiring two nurses, who themselves had to be in strict isolation.

Staffing has already been referred to in the foregoing remarks. On the occurrence of the Glasgow cases, volunteers were asked for and the whole nursing staff of the hospital came forward. Two senior ward sisters were selected along with the most reliable of the senior probationers, and when the smallpox hospital and cottage were opened, these took up duty. They remained continuously on smallpox duty during the whole of the outbreak, and did not leave the hospital or cottage grounds at any time. At the end of the outbreak, they received four weeks' special leave and a fifty per cent. bonus on their salaries for the time spent on smallpox duty. The sickness rate was low and only two nurses had to be relieved of duty on account of septic fingers. All domestic duties were undertaken by the nursing staff, no suitable female

domestic staff being available. One of the former members of the male porter staff came out of retirement to act as gatekeeper, porter, and general handyman, and proved a most useful member of the staff.

During the outbreak visitors to the City Hospital proper were limited to those visiting seriously ill patients and those coming to receive patients on discharge. This regulation was apparently irksome to certain of the tuberculosis patients who attempted occasionally to contrive their own visiting arrangements at remote parts of the hospital perimeter.

Disposal of the Dead : Cremation is undoubtedly the ideal method for cases dying from smallpox and it is gratifying to record that this method was selected by the relatives of all cases dying in the Edinburgh area.

CLINICAL ACCOUNT OF THE OUTBREAK

A male patient, aged 46 years, notified as observation chickenpox, was admitted to the City Hospital on 28th October 1942. After examination, certain features suggested the possibility of smallpox and within a couple of hours he was transferred to the isolation accommodation set apart for that disease by previous arrangement, and although the patient was still regarded as under observation, full precautions were taken as if, in fact, the diagnosis of smallpox had been confirmed. The final diagnosis was not made until 1st November, on which day the diagnosis of smallpox in a second patient, a male, aged 12 years, was arrived at, and accordingly the smallpox hospital was opened. On 5th November, three more cases of smallpox were admitted, and from that date onwards further cases came in until 30th December, when the last patient was brought in. In all, thirty-three cases of smallpox were admitted to the City Hospital, and during the relevant period twenty-three patients were also sent in as observation smallpox but were eventually otherwise classified. In addition, there were three patients who were infected in Edinburgh, two of whom were admitted to other hospitals, while the third died before admission.

Age and Sex Distribution of the Cases :

The following table (Table IV.) shows the sex and age distribution of the thirty-six cases :—

TABLE IV.

Age Groups	0–1	2–4	5–9	10–14	15–19	20–24	25–29	30–34	35–39	40–44	45–49	50–54	55–59	60–64	65–69	70–74	75–79	Total
Male .	–	–	1	1	3	–	4	1	3	–	1	–	2	1	3	–	1	21
Died .	–	–	–	1	–	1	–	–	1	1	–	–	1	–	–	–	–	5
Female	1	–	–	–	2	1	–	–	–	–	2	–	1	–	–	–	–	7
Died .	–	–	–	–	–	–	–	–	–	–	–	–	3	–	–	–	–	3
Total .	1	–	1	2	5	2	4	1	4	1	3	–	7	1	3	–	1	36

The eight deaths give a case fatality-rate of 22·2 per cent.

No deduction can be drawn from the age and sex distribution of the patients.

Nearly every age group is represented and the preponderance of adolescent and adult males in the series is accounted for by the fact that the bulk of the patients were infected in a male ward of the Royal Infirmary, in which, as a rule, the majority of patients are grown-up. The case fatality of 22·2 per cent., when compared with outbreaks of variola major in the past, cannot be regarded as unduly unfavourable. In the following tables, and in the Summary of Cases (Appendix II.), the data concerning the three patients who did not come into the City Hospital are included for the sake of completeness. We are indebted to the Medical Officers of Health of Aberdeen, East Lothian, and Lanarkshire for the relevant information concerning these cases.

Initial Diagnosis :

The outstanding clinical features of all the cases are summarised in Appendix II. As, however, our experience was the common one, viz., that so much depended on the diagnosis of the first case, the main features are described, as are those of the second, since, taken together, they established the diagnosis.

The first patient had been admitted to a surgical ward of the Royal Infirmary on 5th October and, apart from movements detailed elsewhere in this report, remained continuously in that institution until his transfer to the City Hospital on 28th October. On 23rd October his temperature rose to 100° F., and from then onwards till his admission to the City Hospital ran between 100° F. and 101° F. With the pyrexia, the patient stated that he had been out of sorts, had lost his appetite, but had no nausea, vomiting, headache, backache, or chills. A generalised rash appeared on the night of 27th October and when admitted to the City Hospital on the 28th it was well marked on the brow, but scanty otherwise on face and scalp ; well marked on the trunk both back and front with little difference in density between the upper and lower parts in these situations ; well marked on upper extremities, but tapering off in density below the elbows, although lesions were noted on wrists and palms, the axillae being well filled so that the density there was comparable to that on the body ; on the thighs, the lesions were less profuse and none were noted below the knees except a few ill-defined maculo-papules on the soles. The buccal and faucial mucosae were studded with small sloughs. The rash on the body was mainly vesicular and on the face and extremities maculo-papular or papular. On the front of the right wrist, however, one vesicle was characteristically variolous and in a more advanced stage than the others, and it was this more than anything else which influenced us to put the patient under observation for smallpox. The great preponderance of the vesicles on the trunk were superficially set in the skin, oval in shape and irregular in outline, although some were circular. A history of vaccination in infancy and during the 1914–18 war was elicited, two typical well foveated scars being present on the right deltoid region, while one depressed scar on the right shoulder was very doubtful.

From this account it will be appreciated that, however obvious it may have become in retrospect, the diagnosis at this stage, when the rash was about 24 hours old, was by no means easy. Certain characteristics of the lesions were suggestive but by no means typical. In its subsequent development till the 1st November, the rash became more profuse and additional lesions appeared right up till, and on, that date, particularly on the face and hands, the final result being that the weight of the rash was chiefly on the face, next on the

trunk and upper part of the arms, where the densities were about equal. Lesions on the palms by this time had appeared. The original differential distribution between thighs and legs was maintained, viz., the lesions were twice as thick above the knees as below and only a few lesions appeared on the soles. Although scabbing had appeared in lesions on the back as early as 28th October (the third day of the rash), and a number had aborted, the majority remained remarkably monophasic, so that by 1st November the rash was mainly pustular. Many of the lesions, particularly on the face and arms, had become definitely spherical, the earlier elements on the trunk retaining their oval superficial character till the end, the scabs being shed very early. The anomalies appearing during the development of the eruption were probably due to the modifying influence of previous vaccination.

The second case in the outbreak, which was also classified as smallpox on 1st November, could hardly have been diagnosed as such on admission. He was a male (unvaccinated), aged 12 years, and was sent in from the Out-Patient Department of the Royal Infirmary about midnight of 31st October-1st November for observation as cerebro-spinal fever. On admission he showed definite signs of meningeal irritation, a few discrete papules on the chest and upper part of back, a temperature of 103° F., and a pulse rate of 138. Immediate lumbar puncture yielded 30 c.cm. of cerebro-spinal fluid under moderate pressure, and this was reported on in the course of the following day as showing no abnormality. During the first twelve hours after admission, however, the rash developed further, and at 2.30 p.m. on 1st November it was noted that, in addition to the original lesions, fine unobtrusive maculo-papules had appeared on brow, cheeks, neck, and arms.

Dr Archibald, of Belvedere Hospital, Glasgow, who had been called in consultation on the first case, and who had confirmed our decision to treat it administratively as smallpox, was still in hospital when the possibility of the second case being one of smallpox was first entertained. On learning that the second patient had received surgical treatment in the same ward as the first, and after examination of the former, Dr Archibald, from his wide experience, was strongly of the opinion that it was smallpox, and that, taking both together, there could be no possible doubt as to the diagnosis. This was amply confirmed by subsequent events, the second patient dying on the thirteenth day of confluent smallpox. This second case well verified the prediction of Ricketts that " the mention of the last disease [meningitis] suggests that were epidemic meningitis commoner the eruptions which are a feature of it might sometimes cause it to be mistaken." Now that cerebro-spinal fever has become very much commoner, particularly in the war years, the second patient as he was seen on admission was clinically more characteristic of that disease than quite a number of the six hundred cases seen at the City Hospital during the past four years.

Clinical Types : On the basis of the usual method of classification, six cases were classified as hæmorrhagic, five as confluent, three as semi-confluent, eleven as discrete, and eleven as discrete sparse. In the two last-mentioned groups, rashes varied from copious in Cases 6, 13, and 21, to very sparse in Cases 9, 10, 16, 17, 20, 22, and 36. But for the epidemiological background it is more than doubtful if Cases 17 and 20 would have been recognisable as smallpox in the first days during which they came to our notice, although as time went on close clinical observation established the diagnosis.

Fatal Cases : All the six hæmorrhagic and two of the confluent cases were fatal, death occurring in the hæmorrhagic group on the 4th, 5th, 7th, 9th, and 10th days (2 cases), and in the confluent cases on the 11th and 13th days of illness.

Onset of Illness : All patients who were under observation from the onset of their attack presented a well-marked initial illness and nearly all who came late into the City Hospital described one. This was sudden in onset and most patients could give the day with certainty. The symptoms varied widely, by far the commonest being headache, which was complained of by twenty-one patients. Next in order of frequency of complaint were shivering or chills (11), nausea or vomiting (8), backache (8), malaise (7), sore throat (3), perspiration (3), and feverishness (2). Tiredness, photophobia, thirst, delirium, loss of appetite, giddiness, general pains, drowsiness, abdominal pain, coryza, and insomnia were additional symptoms mentioned by individual patients.

Pyrexia was usually well marked in the initial stage, and of sixteen patients in which we have temperature records at the relevant period, the highest temperature was between 104°–105° F. in one, between 103°–104° F. in six, between 102°–103° in three, between 101°–102° F. in three, between 100°–101° F. in one, between 99°–100° F. in one, and between 98·4°–99° F. in one. The highest initial temperatures were not necessarily encountered at the commencement of the worst cases, and in some mild modified cases (*e.g.*, Case 36), a well marked elevation in the initial stage was the only deviation from normal. In all cases there was a reduction of temperature and a subsidence of symptoms with the appearance of the true rash.

Prodromal Rashes : The association of these with the hæmorrhagic cases is described later. In only one case (Case 10) under our observation from the beginning could a somewhat faded uniform erythema in the bathing-drawers' area be discerned in a patient with a greatly modified eruption. In another patient (Case 13), who had been treated with sulphadiazine, a morbilliform eruption appearing on the legs on the fourth day of the true rash was classified as a drug rash.

The True Rash : This appeared on the fourth day of illness in twelve patients, on the third day in ten, on the second day in six, on the fifth day in six, and on the sixth day in two. When observed from its earliest stages, it usually appeared on the face, but this was not always so, the sides and root of the neck being the areas of first appearance in certain patients (Cases 22 and 33), and in one hæmorrhagic case (Case 34) it appeared on the trunk, upper arms and thighs, and did not invade the face till the next day. Even when the face was the first to be involved, the brow was not necessarily the part on which it was seen, the neighbourhood of the naso-labial fold and the chin frequently being the sites of primary involvement. The full development of the rash from its first appearance was occasionally tardy, and though it had usually reached this stage by the third or fourth days, in some the eruption was not completely developed numerically till the fifth day (*e.g.*, Case 33).

In maturation the eruptions were in accordance with the text-book accounts in character and time of appearance of the successive stages, such a feature as " shottiness " in the papular stage being very obvious. The monophasic

character of the bulk of the eruption was also quite striking once the vesicular stage had been reached, in practically all but the much modified cases, the emphasis placed in this in diagnosis by most authorities not having been over-stressed. Evidence of modification was shown by the sparseness of the rash, coupled with a speeding up of maturation. Another feature of modification was morphological changes in the character of the lesions themselves. Some conformed to the usual description, but were very superficial or showed irregularity in size and shape. In others, at the papular stage, the lesions became prominent, conical, and fleshy with on their summit a short-lived vesicular stage merging into small yellow pustules which rapidly crusted. These crusts were by no means always superficial, indeed the patient (Case 21) who had the longest stay in hospital showed a rash of this kind, and it was many weeks before the pin-head scabs were finally shed from scalp, pectoral and scapular regions.

The great majority of cases followed Ricketts' law of distribution once full efflorescence had been attained, such anomalies as occurred being usually in discrete modified cases in which there never was much doubt about diagnosis. For example, in one patient with a scanty discrete eruption the majority of the lesions were in the scapular region. Again in another, the distribution of the rash was typical except that it avoided the face although present in quantity on the scalp. In still another with a sparse discrete eruption, there was no difference in profusion of the rash as between the body and limbs, and lesions were absent on hands, palms, feet, and soles. At the other end of the scale of severity, these anomalies were infrequent, as might be expected, although one patient suffering from a confluent attack died on the thirteenth day of disease with lower forearms and hands, lower legs and feet almost free from rash (Case 2).

Modification of the distribution of the rash produced by pressure or other irritant was not seen except in one patient, a young female, who showed a well-marked garter pattern formed by confluent lesions on the left thigh (Case 27). In one patient whose arm had been encased in plaster for some weeks prior to the outcrop of the rash, there was no difference in the appearance of the protected limb compared with the other when the splint was removed in the pustular stage (Case 15).

Course of the Disease, Convalescence, and Complications :

Whilst practically all patients showed a well marked primary fever followed by a remission in temperature, only fourteen experienced a definite pyrexial reaction characteristic of the secondary fever, and this showed all gradations in extent from a minor elevation of one or two degrees lasting for a few days to a prolonged remittent temperature curve occasionally reaching 103° F. and prolonged for three weeks. As might be expected, the latter was noted particularly in the confluent cases. The older physicians have observed that the natural history of this stage of smallpox is the natural history of the rash, and this was well exemplified in our confluent cases.

During the early part of the secondary fever they suffered considerable pain from the lesions in the pustular stage, and swelling of the face, extremities, and genitalia produced discomfort and stiffness. They were irritable, restless, and slept indifferently. The mouth and tongue were often dirty and invariably there was a well marked catarrh of the upper respiratory tract which resulted

in some cases in laryngitis and pain on swallowing. With the appearance of crusting, the general condition improved greatly, and separation of the crusts proceeded so rapidly at first that it was no uncommon experience to find the patient lying in a pool of scabs.

Convalescence was uneventful except for the occurrence of boils, the only other complications being simple conjunctivitis in two patients and prostatic abscess in another. Except in the modified cases pigmentation was very obvious for weeks and scarring was quite definite, but even in the best marked examples of this there did not appear to be destruction of the true skin to any great depth, and while in the recovered confluent cases the scarring on the face was extensive, it remained fairly superficial.

Confluent Cases : From the text-book point of view the group of five confluent cases was typical as regards distribution, morphology, and evolution of the eruption. One or two of the rashes were suspected to be confluent even in the papular stage, a fact that was appreciated more by palpation than inspection of the skin of the face; but this feature did not become evident to the eye until the rash had been in the fully developed pustular stage for one or two days, viz., on the 8th or 9th days of eruption, although patches of confluent lesions might appear round the nose on the seventh day. At this point the appearance of the patient was dreadful, the face covered with a continuous layer of pustules, greatly swollen, with lips protuberant, oedematous, and sharing in the pustulation. Examination of the mouth was difficult, but faucial, buccal and palatal mucosae were inflamed and covered with sloughs, and the tongue swollen, dirty, and fissured.

Patients invariably complained of a cold with a harsh, painful cough. Laryngitis resulted in hoarseness and great pain on swallowing. The appearance of the face and brow was repeated on the upper part of the back, the extremities, and to some extent on the chest and genitalia, where, however, the lesions were less dense. The patient's general condition was at its worst, the secondary fever, as well marked as the primary in all confluent cases, was at its height, and temperatures of 102° F. to 104·6° F. were recorded at this stage. The pulse was rapid and compressible, and respirations were accelerated to 24 or 26 per minute. Though he lay in a low moaning semi-stupor, he could be roused, his main complaint being of pain from the eruption on the face and hands.

A fact noted by the medical and nursing staffs in attendance was the facility with which patients, even in this critical stage, could be persuaded to take nourishment, and probably the worst case to recover had a three-course dinner throughout the febrile part of his illness. Despite this, rapid wasting characterised the period. With the advent of crusting on the face, the condition became more bearable, but by this time an intense inflammatory reaction had appeared in the normal skin between the pocks, *e.g.*, on the chest, or large blebs about the size of half a dozen lesions formed in the arms or back, and both these processes resulted in extensive exfoliation of the cuticle which was thrown off in sheets, leaving a raw, dirty, granulating surface. In one patient a cast of the palm of the hand including all local seeds came away, removing seeds as well as intervening skin.

As crusting proceeded over the face and body, the general condition improved, and this was reflected in the expression of the eyes, which, clear and alert in the spectacle-shaped normal skin of the orbit, looked somewhat incongruous

in the almost continuous scab which encased the rest of the features. Pocks on the distal parts of the extremities did not always undergo the process of rupture and scabbing, in a number the contents seemed to diminish, the pock became flabby, and eventually dried up to form a seed in the substance of the skin rather than an excrescence in the shape of a crust. This process was most noticeable on the lower legs and dorsum of the feet. With the casting off of the scabs the general condition rapidly improved, sleep, appetite, and weight were regained, and in spite of extensive skin destruction the granulations cleared up remarkably and healing was rapid.

Boils and abscesses occurred in all the confluent cases during convalescence, but only in one could they be regarded as interrupting this stage seriously. As might be expected, scarring was extensive in all confluent cases. This was particularly obvious on the face, but though widespread, being uniform and relatively shallow, it was hardly perceptible at a few yards. Such deep pitting as there was occurred on the tip of the nose, and deformities, *e.g.*, ectropion, were absent. Considerable quantities of hair were lost, but this was not permanent, as the worst confluent case to recover, although he left hospital completely bald with his scalp apparently replaced by scar tissue, returned to visit us some months later with a profuse growth of silky black hair.

It has already been noted that two fatalities occurred in this group. One patient suffered from a profound toxaemia in the initial stages, which resulted in a well-marked myocarditis, and although the evolution of the rash proceeded in its usual remorseless fashion, evidence of circulatory enfeeblement was forthcoming in his failure to react with a secondary fever and in the comparative paucity of lesions on the distal parts of the limbs. For twenty-four hours before death, he was cyanosed, and his hands and feet were blue and cold.

It is debatable whether the other fatal case was really confluent according to the commonly accepted definition. The rash was copious on the face, but certainly was not confluent. All over the lateral surface of the left thigh and leg, however, the eruption was definitely confluent, and the patient was a poor subject, as, when overtaken by smallpox, he had recently had performed a suprapubic cystotomy for the relief of urethral stricture and bilateral hydronephrosis. In the twenty-four hours preceding death he bled profusely *per rectum*, but there was no other sign such as skin hæmorrhages which would justify his classification as a hæmorrhagic case.

Hæmorrhagic Cases : These formed a high proportion of the Edinburgh group of infections, six out of thirty-six, and of these, three were females aged 58 (two cases) and 59 years respectively, and three males aged 55, 42, and 22 years respectively. Of the four cases of this type admitted to the City Hospital, one female patient was admitted very toxic with an intense dusky erythema involving the face, trunk, arms, and proximal part of the legs. In character it was a good imitation of a scarlet fever rash, although the individual puncta were larger, while scattered irregularly over the body were deep violet hæmorrhages the size of lentils. Other lesions in this patient are best described as small hæmorrhagic blebs deeply set in the skin substance. Although she was admitted in the fourth day of illness, there was no sign of a focal rash, and this was still absent on the fifth day when she died. During the period of observation in hospital, the hæmorrhages increased in number, and shortly before death a copious uterine bleeding occurred. Another exceptional feature

of this case was the intensity of lumbar pain, which caused her to cry out continuously almost until the end.

A male patient, on admission, showed a confluent rash of classical variolous distribution set on a background of a generalised dusky erythema on face, trunk, and upper limbs. The focal rash was in the papulo-vesicular stage, and where vesicles had formed they were flat and poorly filled. There were many hæmorrhages into the vesicles, and scattered about in the intervening skin were indigo-violet spots from 2 or 3 mm. to 7 or 8 mm. diameter. A confluent vesicular eruption covered the palate and the buccal mucosa. In such a case the initial toxaemia must have been profound, but that did not prevent the patient struggling out to consult his doctor on the fifth day of illness, and only a little more than twenty-four hours before he died. Consciousness was retained almost till the end, which was preceded by hæmorrhage *per rectum* and a change in the erythema which assumed a leaden or smoky appearance.

The two remaining cases, females, were both similar in character. They showed confluent rashes of typical distribution which developed gradually until the vesicular stage, at which point the preponderance of vesicles became hæmorrhagic in character. In one patient pin-head hæmorrhages, bright red in colour, appeared in the skin contrasting with the dark blood-stained blebs formed by the fusion of groups of lesions. Later these ruptured, especially on the back, leaving extensive raw surfaces. In this patient, also, death occurred from heart failure. In the other the hæmorrhages were confined to the vesicles, but there was a continuous intractable epistaxis for forty-eight hours preceding death, which was due to pulmonary oedema.

State of Vaccination of the Patients :

Among nine unvaccinated patients, two died ; among eighteen vaccinated there were six deaths ; and among nine who had been vaccinated and revaccinated there were no deaths. From the following table (Table V.) the state of vaccination according to age will be seen :—

TABLE V.

Age Grouping of Patients according to State of Vaccination.

Age		Unvaccinated	Vaccinated	Revaccinated
0–9		2	Nil	Nil
10–19		5 (1)*	2	Nil
20–29		2 (1)*	2	2
30–39		Nil	4 (1)*	1
40–49		Nil	3 (1)*	1
50+		Nil	7 (4)*	5
	Total	9 (2)*	18 (6)*	9

* Figures in brackets represent the fatalities.

The fatalities occurring in five instances among the vaccinated were after the lapse of twenty-four (2 scars), forty-two (1 scar), fifty-five (2 scars), and in two cases, fifty-eight (1 scar in each) years since vaccination. The first mentioned, Case 8, prior to his attack, suffered from a serious condition, bilateral hydronephrosis, for which he had recently undergone operation. The remaining fatal case among the vaccinated had been vaccinated fifty-nine years (1 scar) before his attack, and successful revaccination seven days before

the eruption of the focal rash failed to avert the hæmorrhagic form of the disease. It may be of interest to note that all fatal cases in this group, except Case 8, were of the hæmorrhagic type.

The protective value of revaccination is brought out by the fact that no deaths occurred among nine revaccinated patients, half of them in the most advanced age group. When the patients were classified according to type of attack and state of vaccination, the protective value of revaccination is again suggested. No revaccinated patient suffered from an attack more severe than semi-confluent, whilst in the majority the disease took a mild form. Table VI. shows the type of attack according to state of vaccination.

TABLE VI.

Type of Attack according to State of Vaccination.

	Discrete Sparse	Discrete	Semi-confluent	Confluent	Hæmorrhagic	Total
Unvaccinated . .	Nil	3	1	4 (1)*	1 (1)*	9
Vaccinated . . .	6	5	1	1 (1)*	5 (5)*	18
Revaccinated . .	5	3	1	Nil	Nil	9

* Fatal cases in brackets.

For the purpose of classification in the above two tables (Tables V. and VI.), cases developing the focal rash of smallpox less than nine days after successful primary vaccination are regarded as unprotected, and those with an apparent take after revaccination less than nine days before the focal rash are regarded as possessing the protection conferred by primary vaccination only. Only those cases with definite vaccination marks on admission to hospital were accepted as having a previous history of successful vaccination.

Successful Vaccination during Incubation and Initial Period of the Disease :

This was noted in fifteen patients, the results being open to the usual reservation that some of the takes in the days shortly before emergence of the rash may have been due to a determination of the variolous rash to the site of irritation caused by vaccination. The results are tabulated in the following Table VII.

TABLE VII.

Duration in Days of Interval between Successful Vaccination or Revaccination and the Outcrop of the Rash in Fifteen Patients.

	Days													
Type of Rash	14	13	12	11	10	9	8	7	6	5	4	3	2	1
Discrete sparse . . .	–	–	–	1	1	–	–	–	2	1	–	–	–	–
Discrete	–	–	2	–	–	2	2	–	–	–	1	1	–	–
Confluent	–	–	–	–	–	–	–	–	–	–	–	1	–	–
Hæmorrhagic . . .	–	–	–	–	–	–	–	1	–	–	–	–	–	–
Total . .	–	–	2	1	1	2	2	1	2	1	1	2	–	–

In all cases the type of attack was discrete or discrete sparse with two exceptions. One patient revaccinated seven days before the true rash emerged, contracted a hæmorrhagic attack, whilst another primarily vaccinated three days before the rash suffered from a confluent attack. The cases successfully vaccinated or revaccinated twelve days before the rash aroused some comment as it had been assumed that vaccination performed during the first three days of exposure would confer complete immunity. This assumption is not in conformity with Ricketts' view, which was that while it was accurate, in the main, to say that successful vaccination done in the first seven days of exposure would wholly prevent attack, there were occasional exceptions to this rule, and a patient may be vaccinated as early as the fourteenth or fifteenth day before the outcrop and yet not escape the disease.

In the above series there were eight cases successfully vaccinated or revaccinated in the first seven days of exposure, and these give point to his statement that " protection against smallpox can never be promised confidently if its acquisition be postponed till after exposure." The fact that a fatal hæmorrhagic case occurred although the revaccination was apparently successfully performed seven days before the focal rash appeared, indicates that revaccination at this period cannot be relied upon to secure modification of the disease.

Diagnosis :

Although there were several patients in whom the rash was very scanty, it was possible eventually to come to a definite conclusion in all. The word " eventually " should be stressed, since on account of the relative tardiness in development of the rash, immediate clinical diagnosis cannot always be possible, particularly in the first case in an outbreak at the present time when smallpox is to be numbered among the clinical rarities. This is unfortunate, as if the one prophylactic measure upon which all others depend, viz., vaccination, is to be effective or relatively effective, it must be performed immediately.

No laboratory test has so far appeared which is of assistance at the critical early period, and unless the first case happens to be of the toxic variety with widespread purpura or shows the characteristic " bathing-drawers " prodromal rash, it is difficult to see how this difficulty will be circumvented. On the other hand, the presence of smallpox in an area leads to the suspicion that every patient bearing an unusual rash, or even a rash of any sort, is a case of that disease. A good many such cases were brought to our notice, but again observation for a few days, if it could not always establish a final diagnosis, usually sufficed to exclude smallpox.

It is not intended to enlarge upon the clinical features by which a diagnosis of smallpox may be made; adequate descriptions of the disease are given by many authors, both ancient and modern, and in this connection particularly would we recommend the monumental work of Ricketts and Byle. We have in this volume a classic which is unlikely to be surpassed, and doubtless those with a much wider experience of the disease than ours would agree there is nothing about the clinical diagnosis of smallpox which was unknown to or unrecorded by these authors.

Among the large number of cases for which expert opinion was sought, the following twenty-three cases were sent in to the City Hospital by members

of the medical staffs of the city and adjacent authorities, for observation. Of these, three were diagnosed as vaccinia (auto- or hetero-inoculated), one accidental vaccinia rash, two vaccination reactions (pyrexia in successfully vaccinated contacts), one scarlet fever, two enteric fever (*B. typhosus*), one chickenpox, one measles, one scabies, one concurrent scabies and pediculosis, one influenza, one generalised tuberculosis, seven dermatitis, and one urticaria.

Making the distinction from smallpox in all cases resolved itself into careful observation of the distribution and evolution of the rash. In this connection, observation of the cases of vaccinia was interesting as, of course, the morphology of the eruption was exactly that of smallpox, and in two of the cases there was considerable constitutional disturbance because in both the vaccinial material had been implanted in raw weeping surfaces, the result of old-standing eczema, and the resulting clinical picture was that of a confluent smallpox eruption localised to the eczematous sites. In both, however, the distribution was conclusive against smallpox. The accidental vaccinia rash was maculo-papular in character, limited entirely to the trunk, and accompanied by joint pains and a well-marked pyrexial reaction. The two patients showing a post-vaccinial pyrexia were sent in as a precautionary measure on the first sign of a temperature as they had been contacts of known cases.

Treatment :

Apart from symptomatic treatment, including hypnotics which the majority of patients required in the early stages, sulphonamide drugs were employed in fourteen cases, sulphanilamide in six in a total dosage of 24–30 grammes, sulphapyridine in six in a total dosage of 20–35 grammes, and sulphadiazine in two cases, one receiving 54 and the other 21 grammes. One hæmorrhagic, all the confluent and semi-confluent and the sharper discrete cases were treated. In considering the results it is difficult to find any direct evidence of the value of the administration of these drugs. The small number of cases of each clinical variety, particularly the confluent, in which the sulphonamides might be expected to yield the greatest success, and the absence of controls, did not permit the application of the most critical test of all, namely, their effect on fatality rates.

In the confluent cases the secondary fever did not appear to be much affected, and all recovered cases suffered from boils to a greater or lesser extent, but in only one could they be regarded as having interrupted convalescence, and no patient suffered from cellulitis. One feature of convalescence has been remarked on already, namely, the remarkably rapid healing of septic raw surfaces after exfoliation of cuticle, and this may well have resulted from the use of jelonet and sulphanilamide powder. Eye complications were pleasingly infrequent, only two examples of mild simple conjunctivitis being observed.

One example of prostatic abscess has been mentioned, and this occurred in a patient who had been receiving sulphadiazine. The scarring in recovered cases after severe attacks has already been described, and no demonstrable effect on the evolution or drying up of lesions was evident. In any case, the state of vaccination is so intimately bound up with this feature of the rash that it would be impossible to make any useful observations on this point in view of the small number of patients treated.

One of our Scottish colleagues, Glen (1943), has drawn attention to the

lack of odour in the Glasgow smallpox wards where sulphonamides were in use, but this could not be said of the conditions obtaining in Edinburgh. In spite of the lavish use of deodorants and very free ventilation, the all-pervading smell of corruption was ever present. In summing up, therefore, and as far as our limited experience goes, an open verdict must be returned. No spectacular results were obtained at any stage of the disease, although the relative absence of eye and lung complications and the rapid clearing up of raw surfaces may be suggestive.

All patients were retained in hospital until every crust had been shed, and in some cases this involved a prolonged and tedious convalescence, some of the small deeply-embedded scabs left after abortive eruptions presenting the most difficult problems. The appearance in many cases of subungual discs late in convalescence, about which reference in the text-books is scanty or non-existent, presented a difficulty also. They often appeared when exfoliation had nearly been completed and, although some have expressed doubt as to their power of infecting others, we thought it unwise to take risks. It seemed rather drastic, however, to remove the nail, whilst on the other hand to mark time till the discs grew out with the nail seemed unjustifiable. Eventually the problem was solved by filing the nail down to the level of the disc, whereupon it was easy to dispose of the fine dry powder of which it consisted.

VACCINATION AND ITS SEQUELAE

Technique of Vaccination :

During the early days of the smallpox outbreak in Glasgow, certain recommendations were made by the Department of Health in an effort to obtain uniformity of vaccination technique. Accordingly, private practitioners and all medical officers of institutions, etc., were circularised and advised as to the method to adopt.

In June it was recommended that the method of vaccination should be three linear insertions, each ⅝ inch in length and ¼ inch apart. Since this method gave rise to some rather severe reactions it was decided to modify the technique. In consequence, the vaccination was restricted to one linear insertion ⅝ inch long in place of the three insertions previously recommended.

From the first it was advised that all primary vaccinations should be restricted to one linear insertion ⅝ inch in length. Consent was required in writing from those who were being vaccinated primarily or from the parents of those persons under 21 years.

With the appearance of smallpox in Edinburgh, further modification of the vaccination technique was deemed necessary by us. We recommended that each vaccination should consist of three parallel strokes, each ¾ inch in length and ¼ inch apart. Primary vaccinations again consisted of one insertion.

Cross-hatching was deprecated from the very commencement of the Scottish outbreak, and very few instances of this technique came to our notice.

Cleansing of the skin varied with each operator, some using soap and water, followed by a thorough drying of the skin before application of the lymph. Methylated ether was the favourite cleansing agent of many, but methylated spirit or surgical spirit were seldom used and never recommended.

After the operation of vaccination had been completed, the lymph was allowed to dry, giving at least ten minutes to permit of this occurring. As a general rule, dressings were applied to the vaccination areas after the lymph had dried. If no dressing was applied, the patient was advised to cover the area whenever a vesicle appeared. There was no evidence that the presence or absence of a dressing increased or diminished the number or occurrence of " bad arms."

Other recommendations made were that lymph batch numbers should be kept in all instances for every person vaccinated to facilitate inquiry should complaints be made as to the efficacy or strength of the lymph used in particular cases ; that all lymph was to be used as conservatively as possible, and wastage reduced to the minimum.

Sterilisation of instruments between each vaccination was, of course, insisted upon, as were other details intimately connected with the actual procedure of vaccination.

Analysis of Vaccination Figures :

During 1942 it was estimated that some 274,411 vaccinations and re-vaccinations were performed in Edinburgh, of which 20,000 were carried out during the Glasgow outbreak of June-July, the remaining 254,411 being done during the Edinburgh outbreak.

Since accurate figures could not be kept of every individual vaccinated, the figures are estimated ones, based upon the lymph issued from the Public Health Department. It has been assumed that the lymph used by private practitioners and medical officers to various institutions was in strict accordance with the dosage set out on the tube labels. While wastage certainly occurred, against this must be set the fact that the smooth flow of lymph from the multi-dose tubes gave, in many instances, a dosage greater than the label indicated, so that these two factors probably cancelled each other to a large extent, allowing us to adopt the estimated numbers as being near the true figure. Any error will have been to overestimate the number of persons vaccinated.

Table VIII. shows the number vaccinated and the place of vaccination for each group.

TABLE VIII.

Place of Vaccination	Number Vaccinated
First-Aid Posts (8th November to 24th December) . . .	174,335
Private Practitioners (estimated)	39,000
Institutions, Public Works, etc. (estimated)	39,000
Schools	2,076
Estimated Total	254,411

Since clerical assistance was available at most of the First-Aid Posts, records of age, sex, and previous vaccinial state of those attending were recorded, and out of the total of 174,335 persons vaccinated at the Posts, we have complete records of 171,181 of them. Table IX. (opposite) shows the analysis of these complete records.

TABLE IX.

Showing the previous vaccinial states in age and sex groups of persons vaccinated at First-Aid Posts for whom complete records are available.

	Previously Successfully Vaccinated							Not Previously Successfully Vaccinated							Grand Total
	Male			Female			Total	Male			Female			Total	
Age Group .	–5	5–15	+15	–5	5–15	+15		–5	5–15	+15	–5	5–15	+15		
Reported after vaccination.	1,450	10,561	17,688	2,042	11,393	40,031	83,165	1,728	5,515	2,540	1,770	5,680	5,453	22,686	105,851
Did not report	940	4,317	12,669	902	4,046	27,000	49,874	1,345	3,149	2,726	1,160	2,604	4,472	15,456	65,330
Totals . .	2,390	14,878	30,357	2,944	15,439	67,031	133,039	3,073	8,664	5,266	2,930	8,284	9,925	38,142	171,181

TABLE X.

Showing the results of vaccination in respect of age, sex, and previous vaccinial state of persons vaccinated at First-Aid Posts who reported back for examination.

	Previously Successfully Vaccinated							Not Previously Successfully Vaccinated							Grand Total
	Male			Female			Total	Male			Female			Total	
Age Group .	–5	5–15	+15	–5	5–15	+15		–5	5–15	+15	–5	5–15	+15		
Successfully vaccinated Nov.–Dec. 1942	1,277	9,919	16,146	1,840	10,680	37,084	76,946	1,672	5,305	2,382	1,710	5,522	5,179	21,770	98,716
Unsuccessful .	173	642	1,542	202	713	2,947	6,219	56	210	158	60	158	274	916	7,135
Totals . .	1,450	10,561	17,688	2,042	11,393	40,031	83,165	1,728	5,515	2,540	1,770	5,680	5,453	22,686	105,851

It will be observed that about 78 per cent. of those concerned had been successfully vaccinated on a previous occasion, the remaining 22 per cent. having either been unsuccessfully vaccinated on a previous occasion or not at all.

The following statement shows the percentage distribution of the two classes :—

Previously Successfully Vaccinated 77·7%

	Male			Female		
Age Group .	–5	5–15	+15	–5	5–15	+15
Percentage .	1·4%	8·7%	17·7%	1·7%	9%	39·2%

Not Previously Successfully Vaccinated 22·3%

	Male			Female		
Age Group .	–5	5–15	+15	–5	5–15	+15
Percentage .	1·8%	5·1%	3·1%	1·7%	4·8%	5·8%

It will be observed that approximately 66 per cent. of the vaccinations analysed related to persons over 15 years of age. Those attending First-Aid Posts were requested to report back after a period of one week so that the result of the vaccination might be observed and recorded. Of the 171,181 persons of whom we have records, 105,851 or 61·8 per cent. returned as requested.

Table X. (on previous page) shows the results of vaccination in respect of age, sex, and the previous vaccinial state of the 105,851 persons who reported back for examination.

Taking the sexes together, the percentages of successful vaccinations distributed in age groups show a uniformly high figure, as may be seen from the following table :—

TABLE Xa.

	Under 5 Years	5–15 Years	Over 15 Years	All Ages
Percentage of successful vaccinations among those previously successfully vaccinated	89·3%	93·9%	92·3%	91·8%
Percentage of successful vaccinations among those *not* previously successfully vaccinated	96·7%	96·7%	94·8%	96·1%
Percentage of successful vaccinations among all cases	93%	95·3%	93·5%	93·9%

Since 65,329 persons who had been vaccinated at First-Aid Posts failed to return for examination after the lapse of a week, it was decided that a sample should be made and visits were paid to 1247 persons in this group, with the results shown in Table XI. (opposite).

TABLE XI.

	Males			Females		
	-5	5–15	+15	-5	5–15	+15
Successful . . .	74	153	269	49	120	461
Unsuccessful. . .	3	8	34	3	8	65
Totals . . .	77	161	303	52	128	526

It was not found possible at the time of the home visitations to make special note of their previous vaccinial states, and this factor has been omitted from the table.

It is shown from Table XI. that 121 or 9·7 per cent. of the 1247 persons were unsuccessfully vaccinated. This compares with the 7·0 per cent. unsuccessfully vaccinated in the complete analysis of 105,851 persons as shown in Table X. Weighting the dice against ourselves, and taking 10 per cent. as a reasonable incidence of unsuccessful vaccination over the whole campaign during November–December 1942, the following table (Table XII.) shows the estimated incidence of successful and unsuccessful vaccination in the total estimated vaccinations, viz., 254,411 :—

TABLE XII.

	Total	Successful	Unsuccessful
Accurate records available. . .	105,851	98,716	7,135
Calculated figure for remainder from 10% factor	148,560	133,704	14,856
Total estimated vaccinations . .	254,411	232,420	21,991,

Vaccinial Reactions :

Severe Local Reactions to Vaccination : Complete and accurate figures of the number of vaccinations giving rise to severe local reactions are not available. A variety of reasons precluded the collection of trustworthy figures. Firstly, the assessment of what was a "severe" reaction varied with different observers. As an example of this factor, it may be remarked that one institution vaccinated over five hundred of its staff, and at the same time and dealing with the same age groups and type of personnel, another institution vaccinated two hundred of its staff. Yet the first institution reported 2 per cent. of its vaccinations as giving rise to severe reactions, while the second institution reported 6 per cent. of its vaccinations as giving rise to severe reactions.

Secondly, it is known that not all of those vaccinated at the First-Aid Posts reported back for dressings if severe reactions developed—many of them no doubt consulted their private practitioners. The percentage of those vaccinated at First-Aid Posts who did return for dressings was of the order of 1·5 per cent. during the first week.

Thirdly, the proportion of severe local reactions occurring in vaccinations performed by private practitioners in their private practices is unknown.

The Eruptions following Vaccination : Fifty-seven cases of eruptions on parts of the body distant from the vaccination area were seen by members of the Public Health Department. Deducting six cases residing outwith Edinburgh, the incidence rate for all post-vaccinial rashes in the city was about 1 : 5000 vaccinations. No doubt many cases in which the eruption was slight or fleeting were not brought to the Department's notice.

Although such skin eruptions have been described by various writers, no uniformity in classification exists. Especially is this variation noticeable in the use of the term " generalised vaccinia."

In the following description of the Edinburgh cases we have classified our cases into

(*a*) Non-Specific Rashes ;

(*b*) Those with Vaccinial Elements.

Non-Specific Eruptions : These are the " toxaemic " or " accidental " rashes of some writers (*e.g.*, Ricketts, 1908). As to their causation two different opinions exist. The first is that they are the manifestations of an allergic phenomenon due to traces of ox lymph in the vaccine (van Rooyen and Rhodes, 1940) ; the second that they are caused by the absorption of toxic products of the vaccine pustule (Ricketts, 1908). It may be that both factors play parts in their causation.

Incidence : Shaw and Stevenson (1939) state the incidence of non-specific post-vaccinial rashes to be 1 : 6000 to 1 : 9000 vaccinations. Calculating from the tables given by Bloch (1942), the incidence following the Glasgow vaccination campaign was about 1 : 5000 vaccinations. Our experience in Edinburgh where thirty-four cases of non-specific rashes were seen gave an incidence rate of about 1 : 8000 vaccinations for non-specific eruptions. It is probable that these incidence rates quoted above are under-estimates, as it is our belief that some eruptions are so mild as not to come to notice.

Types of Eruption : These are shown in following table (Table XIII.).

TABLE XIII.

Erythema Multiforme	Erythema Morbilliforme	Erythema Scarlatiniforme	Lichen Urticatus	Urticaria	Papular Erythema	Unclassified
10	8	2	7	2	1	4

Thus most of the non-specific rashes were classed as erythema multiforme, though almost as many cases were classified as erythema morbilliforme. This preponderance seems to be usual experience. Shaw and Stevenson say that " the morbilliforme eruption is perhaps the most common," and Bloch's figures tend to the same conclusion. " Erythema multiforme " is a somewhat elastic term and a wide range of eruptions may be placed under this particular class by different observers. Lichen urticatus (or papular urticaria) is the " vaccinial lichen " of some writers (*e.g.*, Ricketts). Its occurrence as a post-vaccinial eruption is well known for Ricketts, Chalke (1931), and Bloch all

describe its occurrence. Since " lichen urticatus " is usually attributed by dermatologists to allergens there seems good reason to believe that Ricketts was correct in his opinion that " these lesions are toxaemic and are not evidence of a generalisation of the specific virus."

Age and Sex Distribution are shown in Table XIV. :—

TABLE XIV.

Age	Under 5	5–15	Over 15	Total
Males . . .	3	4	4	11
Females . . .	8	7	8	23
	11	11	12	34

It will be seen that twice as many females were affected as males and that this proportion was, broadly speaking, constant throughout the age groups.

It may be pointed out that of the 171,181 vaccinations availáble for analysis into sex and age distributions, 29,005 males and 29,597 females were under fifteen years, and 35,623 males and 76,956 females were above fifteen years. In consequence, the preponderance in the table of females below fifteen years may have some significance, though this significance is lost in the age groups above fifteen years.

The table shows twenty-two out of the thirty-four cases to be under fifteen years of age. Out of the 171,181 vaccinations available for analysis only thirty-four per cent. were under fifteen years of age so that the preponderance of cases of non-specific eruptions in the age groups below fifteen years may have some significance.

Days after Vaccination when Rash Appeared : This is shown in Table XV.

TABLE XV.

Days after Vaccination . .	4	5	6	7	8	9	10	11	12	13	14	Total
Primary Vaccination . . .	–	2	2	6	3	1	–	–	–	–	2	16
Secondary Vaccination . .	2	1	–	4	4	1	1	1	1	–	3	18
Totals	2	3	2	10	7	2	1	1	1	–	5	34

It will be seen that half the cases occurred on the 7th or 8th day after vaccination. Of the eighteen eruptions occurring in the secondarily vaccinated, eleven occurred on or before the 8th day after vaccination.

A word may be said on the distribution and course of these eruptions. They had no marked predilection for any area of the body, though the face was usually spared. About half the cases showed or gave a history of constitutional upset, but in all these the local vaccination reaction was well marked and the upset may have been due to it. The duration of the non-specific rashes varied from twenty-four hours (in most cases) to seven days (in three cases). No case showed chronicity or left sequelae.

Differential Diagnosis : The erythema scarlatiniforme and the erythema morbilliforme types were distinguished from scarlet fever and measles by the lack of faucial congestion and the absence of coryza respectively. Besides, the rash rarely invaded the face—a point of service in distinguishing it from the true rashes of measles and smallpox. The time interval between vaccination and the appearance of the rash was of some moment in deciding in favour of a non-specific eruption—half the rashes appeared on the 7th or 8th day after vaccination. The absence of pre-eruptive illness was of value in some of the cases.

Eruptions showing Vaccinial Elements —Generalised Vaccinia : By generalised vaccinia is here meant the occurrence of vaccinial lesions on parts of the body of a recently vaccinated person and where auto-inoculation of the virus could reasonably be excluded. Moreover, the suggestion made by Shaw and Stevenson that some vesicular rash must be present to define the condition has been followed. It is, of course, never a certainty that auto-inoculation has not produced those lesions. Some writers (notably Chalke) insist that generalised vaccinia is a great rarity and maintain that in cases of apparent generalised vaccinia the situation of the lesions, the lack of personal cleanliness of the patient, and the presence of an old skin eruption will be noted and will afford " a clue to the real cause of the condition," *i.e.*, auto-inoculation. Nevertheless, in three of our cases the likelihood of auto-inoculation was very remote.

Other observers (Shaw and Stevenson, Jubb (1943)) maintain that generalised vaccinia may occur where no evidence or probability of auto-inoculation can be adduced. Two of our cases, a male and a female, were adults and one was a female child of ten years. The male alone had been vaccinated before and in his case the lesions appeared on the 6th day after vaccination, in the adult female they appeared on the 8th day after vaccination, and in the child on the 9th day. In the two female cases the lesions were scanty (four in each case), and consisted of superficial thin-walled vesiculo-pustules. In the child they lay on the legs, in the adult they lay one on the chin, one on the right deltoid, one between scapulae, and one on the sole of the left foot. The adult male had a more profuse eruption, the lesions being chiefly on the front and back of the trunk and on the upper arms. Several existed on the scalp and neck. The lower limbs were spared. At first the lesions were papular, but in a day's time vesiculation was seen and the rash resolved at this stage.

Autogenous Vaccinia (Auto-inoculation) : By autogenous vaccinia is here meant the occurrence of vaccinial lesions on some part of the body distant from the vaccination area—the virus having been transported from the vaccination area by some mechanical means (*e.g.*, the patient's fingers or some article such as a towel) to the secondary area and there implanted by scratching or rubbing.

Fourteen cases of autogenous vaccinia were seen by the staff of the Public Health Department. Davidson and Davis (1943) reported " Four cases of Generalised Vaccinial Reactions in Allergic Subjects," all of which cases had been vaccinated in Edinburgh during the 1942 outbreak. Of their cases one (Case 1 of their series) was seen by members of the Public Health Department.

The table (Table XVI.) below sets out the distribution in age groups of the cases of autogenous vaccinia seen by us.

TABLE XVI.

Age Groups . . .	Under 5	5–15	Over 15	Total
Males	1	2	4	7
Females	–	1	6	7
Totals	1	3	10	14

Table XVII. shows the days after vaccination on which the rash appeared.

TABLE XVII.

Days after Vaccination	6	7	8	9	10	12	17	Total
Primary Vaccination	–	2	1	2	1	–	–	6
Secondary Vaccination	–	1	–	2	2	1	2	8
Totals	–	3	1	4	3	1	2	14

Most of the rashes appeared seven to ten days after vaccination.

Incidence : Considering the cases of autogenous and generalised vaccinia together, and including the cases recorded by Davidson and Davis, the incidence rate of these forms of vaccina was 1 : 13,390 vaccinations.

The Clinical Course of the Eruption : The local vaccination reaction was in all cases well developed—in five cases it was more than usually intense.

Constitutional upset varied—five of the cases suffered from general malaise with rise of temperature (99°–101° F.).

The eruption was not constant in its quantity or appearance. Three of the cases showed a profuse eruption, the others showed sparse and scattered lesions.

An account of the three cases with profuse lesions may be of interest :—

Case 1.—A woman of 20 years was primarily vaccinated on 17th November 1942. On the 24th she felt ill, and her vaccination appeared as a good " take." On the 26th November a rash appeared on her right cheek, and by the 29th the whole face was involved in a profuse eruption of vesicles, many of which had ruptured and were oozing serum. Marked oedema of the lips and eyelids was present, and the patient was sharply ill—pulse 120, temperature 101° F. A feature of the case was the marked swelling of the upper neck. The scalp was spared. " Cropping " occurred, and convalescence was protracted, but recovery was eventually complete.

Case 2.—A woman of 43 years, who had been vaccinated in infancy, was revaccinated on the 20th November 1942. Eight days after vaccination a good reaction appeared on her arm, and by 30th November this reaction was severe and led to the characteristic deep ulceration. About seventeen days after vaccination a crop of vesicles appeared

on the vulva and spread rapidly into both groins. The eruption was attended by much itching and irritation, and went through a shortened evolution. It occasioned the patient no general upset. A suggestion that the eruption was herpetic in nature had been made, but this was ruled out by a dermatologist who saw the patient.

Case 3.—This patient was described by Davidson and Davis, and is Case 1 of their series.

A married woman of 28 years, vaccinated in infancy, was revaccinated on 16th November 1942. She had for some years previously suffered from urticarial rashes which were extremely pruritic, but at the time of revaccination her skin was unaffected by these. Ten days after revaccination a papulo-vesicular rash appeared, confluent on the left shoulder and well marked on both upper limbs, neck, front of chest, abdomen, groins, and popliteal fossae. Marked oedema and induration were present round the vaccination area. The face, head, hands, and feet were spared. The lesions were hard and "shotty," and many appeared to be set deep in the skin. Next day many of the vesicles had ruptured and were oozing serum. Within the next four days the elements passed rapidly through vesicular and pustular stages, and then to scab formation. The general condition was good throughout all her illness—the temperature never rising above 100° F. She was discharged after nineteen days in hospital, and since that time has remained in excellent health—no sequelae, beyond some very faint scars, remain.

In the cases showing sparse eruptions, the nature, number, and situation of the lesions varied. Six of the eleven cases showed either vesicles alone or a mixture of vesicles and pustules. Three of the cases showed a mixture of papules and pustules. In the remaining two cases the lesions had reached the crusting stage by the time they were seen.

In seven of the cases the number of lesions was of the order of ten to twelve elements. In two of the cases seven lesions were counted, and in the remaining two cases only four lesions existed. No specific distribution was shown by the eruptions and it is impossible to draw generalisations from such a small number of cases. It is of interest that only three cases showed lesions on the face and only three showed lesions on the trunk.

The lesions showed no great variation in size—they were usually set superficially in the skin. The course of the eruption lasted over a few days, and no case showed sequelae.

Differential Diagnosis : Since both generalised vaccinia and autogenous vaccinia show a rash whose elements may closely resemble modified smallpox, their distinction from that disease is a matter of importance. The main points which guided us in the differential diagnosis of vaccinia from modified smallpox were :—

(1) The fact that the patient had been recently and successfully vaccinated was a point against the rash being due to smallpox. Emphasis could not, however, be laid on this since variola can exist with a concurrent good vaccination.

(2) The absence of pre-eruptive illness. This was a feature of six of our cases.

(3) The small number of Edinburgh cases of generalised and autogenous vaccinias gave us no impression of specific distribution. The immunity shown by the face (in all but three cases) was of significance.

(4) The commonly superficial nature of the vaccinial lesions.

It may be said that no case diagnosed either as generalised or autogenous vaccinia came, in retrospect, to be suspected on epidemiological grounds of having been variola.

Heterogenous Vaccinia : By heterogenous vaccinia is here meant the occurrence of vaccinial lesions on some part of the skin of a person who has not recently been vaccinated, and where the virus has been conveyed and implanted by the hand of another person, or, more rarely, by the patient's own hand. The source of the virus is a vaccination vesicle either on the vector's arm or on the arm of some other person.

Six cases of heterogenous vaccinia were observed during the outbreak. Three of the cases occurred in adult women, all of whom had been vaccinated in infancy, and none of whom had been revaccinated during the outbreak. The remaining three cases occurred in children under the age of five years, none of whom had ever been vaccinated. Although seen by the staff of the Public Health Department, four of the six cases resided outside Edinburgh.

Two of the adult cases presented some common features. Both women had dressed the vaccination reactions of children living in the house, and in both the face was the site of the heterogenous lesions.

In the first, the patient's grandchild had been vaccinated on 10th November 1942, and the vaccination area had been dressed daily by the patient from 18th November onwards. On 23rd November the patient developed a papular lesion on the left lower lip, and by the 25th November a few vesicles had appeared on the chin accompanied by considerable oedema. The lesions ran through a shortened evolution, but considerable general upset was experienced for five or six days.

In the second case, the patient's niece was vaccinated on 11th November 1942, and had her vaccination area dressed by the patient from 14th November onwards. On the 22nd November a papule appeared on the patient's left cheek, and by the 24th a cluster of pustules had appeared on the cheek accompanied by brawny induration of the left side of the face. The general upset was sufficient to warrant her admission to hospital. In a few days' time an ulcer formed on the cheek, but it healed rapidly, and convalescence was established within a week.

The third adult was a married woman residing outwith Edinburgh, who had been vaccinated in childhood. On 20th November 1942 her children were vaccinated, with good " takes." The patient herself had had a skin disorder of her forearms for some years, and had not been revaccinated. On 2nd December she felt indisposed, and on 3rd a red macular rash appeared on both forearms. By 5th December this eruption had become vesicular and the constitutional upset was marked. On 6th December she was sent into the City Hospital as a suspected case of variola. She was suffering from sharp

constitutional upset; pulse 108, temperature 100·4° F. A widespread eruption existed. The face was the site of a maculo-papular rash, most marked on the chin. The arms showed a very profuse rash with density increasing towards the wrists and most marked on the flexor aspects. The elements of the rash on the arms were vesicular, some showing umbilication and having a typically variolous appearance. The hands showed a few vesicles on the palmar surfaces. The trunk, both back and front, showed a maculo-papular rash—some vesicles were seen below the umbilicus. The rash on the back was more profuse than that on the front of the trunk. Papules were seen on both thighs and the legs were covered with a less dense maculo-papular rash. The sole of the right foot showed two macules—the left foot showed no lesions. Maculo-papules were present on the palate.

By the next day, *i.e.*, 7th December, the rash on the body, apart from the vesicular elements, had become morbilliform—no itching or irritation was present. The vesicular rash on the forearms had become pustular. By the evening of that day the eruption on the chest and legs was beginning to fade. On the following morning the whole rash was fading. The lesions on the forearms were resolving and the local oedema was lessening. By 12th December large areas of crusts on the forearms had separated, leaving raw surfaces. The condition took another fortnight to heal, but by 29th December the patient was ready for discharge. No sequelae remained.

This was the only case of heterogenous vaccinia in which doubt as to diagnosis was sufficiently strong to warrant admission to the City Hospital for observation. The localisation of the eruption which the other cases of heterogenous vaccinia showed was absent in this widespread eruption. Not only was there a history of pre-eruptive illness, but the appearance and distribution of the rash raised suspicion. It is true that on admission the rash was polyphasic, and that, apart from the forearms and hands, it tended to be centripetal. The subsequent course of the rash confirmed the diagnosis of vaccinia made on admission to hospital, for the vesicles which appeared on 5th December had, by the morning of 7th December, become pustular, and by the evening of that day the rash on the chest and legs was fading.

The heterogenous vaccinias occurring in the three children were of peculiar interest. The cases had certain features in common—(*a*) the children were all under five years of age, (*b*) none of them had ever been vaccinated, (*c*) all were having ointments inuncted for concurrent skin disorders, and (*d*) other members of the household were having vaccinial reactions dressed.

The first child had been sick with bronchitis when his brothers were vaccinated on 18th November 1942. The vaccinations in these led to reactions which required daily dressing of their arms from 24th November onwards. On 6th December the patient was observed by his parents to be scratching his pubic region, and in the belief that he was suffering from scabies, his father, who had been dressing the vaccinial reactions, rubbed in sulphur ointment. By 8th December a large number of vaccinial vesicles had appeared over the scrotum and inner aspects of the thighs. The lesions were confluent, and considerable oedema was present. The child made a complete recovery.

The second case was a child who had attended the Skin Department of the Royal Infirmary a year previously for treatment of Besnier's prurigo. Ichthyol paste had been ordered to be rubbed into the affected areas, and the child was undergoing this treatment at home when his brothers were being vaccinated on

14th November 1942. From 20th November onwards the patient's mother daily dressed the vaccination areas of the recently vaccinated children and then, immediately afterwards, inuncted the patient's skin with ichthyol paste. On 26th November papules appeared on patient's skin confined to the regions inuncted. By 28th November, the day on which he was seen, the eruption was well marked. It consisted of pustules, but still was entirely confined to the areas of the dermatitis, *i.e.*, the anterior aspects of the thighs and the flexor aspects of both forearms. The glands in the axillae and groins were enlarged and general upset was present. Recovery was complete in this case.

The third case was that of a female child, aged two years, who had been under treatment for a skin disorder for several months. Her mother had been vaccinated on 20th November 1942, and that vaccination had been dressed daily from 26th November onwards by the child's grandmother, who also daily inuncted the areas of dermatitis on the child's skin. On 2nd December numerous vesicles appeared on, and entirely confined to, the areas of the skin whereon the ointment had been rubbed. The vesicles were definitely vaccinial in appearance, many being umbilicated. The child's general condition was extremely poor. Although she did not appear toxic, she was very anaemic and ill-nourished, and it was decided to admit her to hospital. Her condition deteriorated there—the eruption became pustular—and she died of a terminal broncho-pneumonia ten days after admission.

The six cases cited above are examples of two different methods by which heterogenous vaccinia may be caused ; in the adults it seems most likely that the patient's own hands had conveyed and implanted the virus from the outside source (*i.e.*, the lesions on another's arm), while in the case of the three children, the virus had been conveyed and implanted by the hands of a vector.

Post-Vaccinal Encephalomyelitis :

As vaccination was being carried out on such a large scale, it was to be presumed that cases of nervous complications might occur (Ledingham, 1934). Since sequelae of vaccination are not notifiable, their occurrence had to be sought by direct inquiry made to the various hospitals in the City. From these and other sources, twenty-three cases of post-vaccinal nervous disease were brought to our notice during the period June 1942–January 1943 inclusive. Of these cases, one occurred in July 1942, when vaccination was carried out on a comparatively small scale, the remaining twenty-two occurring either immediately prior to or during the Edinburgh outbreak. This one case in July, and twelve of the group of twenty-two cases, occurred among Edinburgh citizens, the other ten which were treated in Edinburgh hospitals came from areas outwith the City boundaries where vaccination campaigns were being conducted.

Case Incidence : During June-July 1942 some 20,000 vaccinations and revaccinations were performed in the City, giving, therefore, a proportion of nervous complications of 1 : 20,000 total vaccinations.

During November-December 1942 it was estimated that 254,411 total vaccinations were carried out in the City. These were associated with the occurrence of twelve cases of nervous complications, a proportion of 1 : 21,200.

Nine of these twelve cases were in primarily vaccinated persons, a proportion

of about 1 : 6,300 primary vaccinations ; the other three were in revaccinated persons, a proportion of about 1 : 65,900 revaccinations.

Of the ten cases from districts outwith the Edinburgh boundaries, seven came from two counties, about which information regarding the number of persons vaccinated during November-December has been kindly provided by the Medical Officers of Health of these areas. Four cases came from one county, in which 42,000 total vaccinations were performed, a proportion of 1 : 10,500 ; the other three from a county in which 45,000 total vaccinations were carried out, a proportion therefore of 1 : 15,000 total vaccinations.

Age and Sex Distributions : These are set out in Table XVIII.

TABLE XVIII.

Years	Male	Female
2–3	0	1 (1)
3–4	0	1
4–5	0	2
5–6	2	2 (1)
6–7	0	3 (1)
8–9	0	1
9–10	1 (1)	0
10–11	2	1
13–14	0	1
15–16	2 (2)	0
25–30	0	2
30–35	0	1 (1)
50–55	0	1 (1)
	7 (3)	16 (5)

* Figures in brackets denote deaths.

The three cases following secondary vaccination were aged 26, 30, and 51 years respectively.

There were eight deaths out of the total of twenty-three cases, three occurring among Edinburgh cases and five among the county cases. Investigations were carried out to determine if there was any correspondence with lymph batch numbers, but they were of a negative character.

Technique of Vaccination : The techniques of vaccination varied somewhat in the twenty-three cases, and Table XIX. shows the number of insertions in each of these cases :—

TABLE XIX.

Insertions	Cases
1	4 (Cases 7, 19, 20, 21)
2	2 (Cases 1, 3)
3	9 (Cases 4, 5, 9, 10, 12, 16, 17, 22, 23)
Unknown	8 (Cases 2, 6, 8, 11, 13, 14, 15, 18)
	23 cases

Incubation Period : In calculating the so-called incubation period of the disease, the time between vaccination and the onset of the first definite nervous symptom or sign was taken, since several of the patients had constitutional symptoms consequent upon generalised vaccinial reactions. It will be seen

from the following table (Table XX.) that eighteen of the twenty-three cases had an incubation period of between seven and fourteen days inclusive. There was one case each on the third, fifth, and twenty-second days, and two on the twentieth day after vaccination.

TABLE XX.

										22												
										21												
										16												
										13												
										10	14	19										
						18				9	11	15	20						8			
		23		12		7			1	3	4	6	5						2		17	
1	2	3	4	5	6	7	8	9	10	11	12	13	14	15	16	17	18	19	20	21	22	Days

The table also shows the case numbers of the patients concerned.

Analysis of Symptoms : The disease ran an acute course in all the cases except Case 12, which ran a protracted course. Recovery took place in fifteen patients, and appeared to be complete in them all. Death took place in eight cases, in seven of which it occurred between the fifth and fifteenth day from the onset of symptoms. The remaining case (Case 12) ran a very prolonged course till death from intercurrent infection and exhaustion closed the scene on the one hundred and eighth day of disease.

The onset of the nervous phenomena was, in all cases, fairly well marked, being usually sudden, but even when not dramatic in its occurrence, a definite day could be given on which the nervous symptoms made their appearance. Eight cases had symptoms referable to the vaccination, being either of a local or general character, prior to the occurrence of the nervous symptoms. Local vaccinial reactions were present in all, but were particularly severe in three cases.

Drowsiness : This symptom was present in twenty of the twenty-three cases under review. It was not only the most constant of the early symptoms, but also the most prominent one. It varied in intensity; in some patients the drowsiness was first manifested by a tendency on the part of the patient to drop off to sleep at meals or when sitting in a chair. Others wanted to remain in bed and to continue what was observed to be a very deep sleep from which the patients could only be aroused with unusual difficulty.

The drowsiness in eighteen cases passed into a stupor from which the patient could only be roused by strong stimulation, or into a frank coma when little or no response to stimulation was forthcoming. A period of post-convulsive unconsciousness of some depth but of comparatively short duration occurred in Case 2. Previous to the convulsions the child had shown no signs of drowsiness. Two cases showed deep coma of considerable duration, one remaining comatose for three days, the other for four days. Both recovered completely.

Pyrexia : The presence or absence of pyrexia was obtained in all instance from the hospital records, save in one case. This fact of clinical significance applied therefore only to the condition of the patients when in hospital. Only a few cases gave a history of "feverishness" or definite pyrexia before

admission. In Case 2 the attending physician specifically mentioned the fact that the patient's temperature prior to admission was subnormal at the time of his examination. Normal temperature limits had been attained by the time the child was admitted to hospital some hours later.

Definite pyrexia was present in fifteen cases in the earlier stages of the disease. The range of fever noted in these cases was 99° F.–102° F. on admission. The temperature fell to normal levels a few days after admission to hospital in most of the cases which recovered. In the eight fatal cases the temperature tended to show a progressive rise prior to death, but Case 12, which had a normal temperature on admission to hospital, maintained such normal levels until the supervention of secondary infection, when it began to fluctuate, and continued so till death.

Headache : Headache was present in fifteen cases and was one of the early symptoms of the illness in these cases. In at least nine of the cases, the headache was frontal in situation, was severe in character, and caused considerable distress in some. Case 4 had headache in the temporal regions as well as in frontal area. Headache was present over the vertex in one case, and described as " general " in the remaining five cases.

Vomiting : This symptom was an early one in nine cases. It was sudden in onset, severe and intractable in Case 1, continuing for four days, and ceasing only with the increasing drowsiness of the patient. Vomiting was also severe in Cases 13, 14, and 22, where the hospital records note that Case 13 " vomited excessively " ; Case 14 " vomited almost continuously for two days " ; Case 22 had " been unable to retain anything in the stomach," also for two days.

The vomiting was projectile in one case only (Case 18). Cases 9, 11, 20, and 21 gave a history of vomiting once or twice at the commencement of the illness.

Pain : Pain, apart from headache and the local vaccinial reaction, was complained of in nine cases. The pain was situated in the following regions : back of neck, two cases ; abdomen, four cases ; small of back, two cases ; in legs, three cases ; in ear, one case. This last case never at any time showed evidence of aural infection, though this was constantly looked for. In one of the cases with pain in the neck, there was also pain in both arms. Girdle pains were also noted in Case 12, which presented the signs of complete section of the spinal cord at the level of the sixth thoracic segment.

Convulsions : The sudden occurrence of convulsions ushered in the disease in spectacular fashion in Case 2. This patient had had some local vaccinial reaction, but had not been greatly upset by it. The convulsions were described by the attending physician as being akin to status epilepticus, and required chloroform to control them. Convulsions supervened on other symptoms in a further four cases, occurring in the course of increasing drowsiness in them all. In all these five cases with convulsions, the latter were generalised in character, and were followed by periods of unconsciousness of varying depth and duration. All the patients who had convulsions were children, aged from 4½ to 10 years, and all recovered completely.

Mental Irritability : This symptom occurred in eleven cases. In two it was an early symptom, and in another case it followed convulsions.

Delirium : This symptom was present in nine cases, being early in eight instances, following on recovery from the stupor after convulsions in the remaining case.

Visual Disturbances : Case 22 said that she " couldn't see the end of the bed." This symptom occurred on the second day of disease, continued for forty-eight hours, and was followed by a convulsion.

Trismus : The mother of Case 14 complained that the child could only open its mouth with difficulty, and this was verified by the family physician. The trismus was also noted on admission to hospital. Case 16 also had difficulty in opening the mouth.

Hiccough : Incessant hiccough for about ten to twelve hours was a prominent symptom in the early stages of illness in Case 16.

Paraesthesiae : " Pins and needles " were complained of in Case 13, while Case 12 complained of tingling and numbness of feet and legs.

Weakness of Legs : Three cases complained of weakness of the lower limbs. Weakness, with an increasing dragging of the legs, was a prominent feature in Case 12, finally rendering the patient bedridden.

Ataxic Symptoms : This was the symptom compelling the child to go to bed in Case 10. The mother described the gait as " reeling." Case 12 also had some ataxia.

Of the general symptoms, anorexia was constant though it varied in degree, being seldom complete. Constipation was present in eleven cases, being of an obstinate character in two cases. Diarrhoea seemed to be an early symptom in one case (Case 19). It was of short duration, commencing abruptly, lasting acutely for forty-eight hours, and ceasing only gradually after recovery from a convulsion.

Neurological Data : The cases all resolved themselves into three fairly well-defined groups, viz., a spastic, a flaccid, and an indeterminate group. These groups may conveniently be shown in tabular form.

TABLE XXI.

Spastic Group

Case No.	1	2	3	4	5	11	14
Coma	+	+*	+	—	—	+	—
Neck rigidity	+	+	+	—	+	+	—
Kernig's sign	+	++	+	+	+	+	—
Abdominal reflexes	+	+	+	—	—	—	—
Knee jerks	++	+	++	++	++	++	+
Ankle jerks	++	+	++	++	++	++	+
Ankle clonus	—	—	—	—	—	—	—
Babinski's sign	—	?+	+	+	+	+	+
Upper limb reflexes	+	+	+	+	+	++	++
Clonic movements	—	—	—	—	+	—	—
Urinary incontinence	+	—	+	—	—	+	+
Urinary retention	—	—	—	—	—	—	—
Rectal incontinence	+	—	+	—	—	—	—
Rectal retention	—	—	—	—	—	—	—
	D	R	D	R	R	R	R

* Was post-convulsive in onset. D=died. R=recovered.

The muscular rigidity in Case 2 was so great that a degree of opisthotonus was present. In Case 1 the cervical muscles were very stiff, especially on the right side, producing a well-defined scoliosis of the neck which simulated closely a torticollis. Clonic movements of the legs ensued for a short time after examination of the reflexes of the lower limbs in Case 5, while more generalised movements, tonic in character, followed the slightest stimulus to the body in the early stages of the disease in Case 11.

TABLE XXII.

Flaccid Group

Case No.	7	10	12	18	19	21	22
Coma	+	+	—	++	++	+	+
Neck rigidity	++	—	—	++	—	+	—
Kernig's sign	+	—	—	+	?+	+	—
Abdominal reflexes	—	—	—	—	—	—	—
Knee jerks	—	—	—	—	—	—	—
Ankle jerks	—	—	—	—	—	—	—
Babinski's sign	—	—	—	—	+	+	+
Upper limb reflexes	+	—	+	+	+	—	—
Sensation	+	+	—*	+	+	+	+
Urinary retention	—	—	+	+	—	—	—
Urinary incontinence	+	+	—	—	+	+	+
Rectal retention	—	—	+	—	—	—	—
Rectal incontinence	+	+	—	+	+	—	+
Bedsores	—	—	+	—	—	—	—
	D	D	D	D	R	R	R

* Loss of sensation below level of Thoracic 6.

D = died. R = recovered.

Cases 10, 21, and 22 were deeply in coma on admission to hospital, Case 10 succumbing, while Cases 21 and 22 made complete recoveries. Case 7 recovered partially, but relapsed and died soon afterwards.

TABLE XXIII.

Indeterminate Group

Case No.	6	8	9	13	15	16	17	20	23
Coma	+	—	+	+	+	+	+	+	+
Neck rigidity	+	+	+	+	—	++	+	—	—
Kernig's sign	—	+	+	+	+	+	+	—	—
Abdominal reflexes	—	+	+	—	+	—	?	+	+
Knee jerks	—	+	+	+	+	+	+	+	+
Ankle jerks	—	+	+	+	+	+	+	+	+
Ankle clonus	—	—	—	—	—	—	—	—	—
Babinski's sign	—	—	—	+	—	+	—	—	—
Upper limb reflexes	++	+	+	+	+	+	+	+	+
Urinary retention	+	+	—	—	—	—	+	—	—
Urinary incontinence	—	—	+	—	—	+	—	+	—
Rectal retention	—	—	—	—	—	—	—	—	—
Rectal incontinence	—	—	—	—	—	+	—	—	—
	D	R	R	R	R	R	R	R	D

D = died. R = recovered.

Other Clinical Findings : Pulse : This was slow and of high tension in two cases (Cases 3 and 19), although the temperature was raised in both

instances. All other cases had pulse rates proportionate to the temperature levels reached.

Abdomen : The abdomen was scaphoid in the early stages of the disease in Case 3, slightly sunken in Case 11, and distended from an enlarged and full bladder in Case 12.

Ocular Signs : Mention has already been made that Case 22 complained she could not see the end of the bed. Apart from this one case, no complaint was made regarding visual disturbances by any of the patients.

Squint was conspicuous by its absence in all the cases, contrary to the findings of most writers (*e.g.*, Report, 1928).

The state of the pupils varied in each case, being contracted and equal in a few, dilated and equal in others, but normal and equal in most. Cases 19 and 22 had dilated pupils which were insensitive to light, but which soon recovered this reflex and that of accommodation as the disease process regressed.

The fundus was examined in three cases, viz., Cases 12, 17, and 18. It was normal in Cases 12 and 18, but showed early papilloedema in Case 17. This was ultimately completely recovered from, and Case 22, with visual disturbances, was unfortunately not examined ophthalmoscopically.

Cerebro-spinal Fluid : Lumbar puncture was carried out in nineteen cases and cisternal puncture in one. The findings on examination of the fluid are recorded in the following table (Table XXIV.) :—

TABLE XXIV.

Case	Pressure	Colour	Cell Count	Globulin	Sodium Chloride mgm.%	Sugar mgm. %	Protein mgm. %	Culture
1	normal	contam. blood	–	–	*	62	–	–
2	+	clear	–	–	–	–	–	–
3	?	clear	–	–	–	–	–	–
4	+	clear	–	–	703	60	20	neg.
5	++	clear	25 (L)	–	703	52	25	neg.
6	+	clear	2 (L)	–	720	82	25	neg.
7	normal	clear	–	–	676	53	35	B. coli.
8	+	clear	1 (L)	–	676	46	35	neg.
10	normal	clear	–	neg.	730	62	40	neg.
11	normal	clear	–	neg.	725	74	70	–
12*	65–70	clear	70 (L)	–	726	66	175	neg.
13	+	clear	–	Tr.	732	77	45	neg.
14	normal	clear	–	neg.	730	71	20	–
16	+	clear	2 (L)	neg.	719	63	30	–
17*	300	clear	120 (L)	–	–	–	90	–
18*	(*a*) 144	clear	100 (40% P)	–	700	63	70	neg.
	(*b*) 225	clear	137 (P)	–	766	87	120	neg.
19	+	clear	–	–	760	104	60	neg.
20	+	clear	–	–	721	106	40	neg.
21	+	clear	–	neg.	750	102	60	neg.
22	normal	clear	–	neg.	741	106	50	Staph. albus

* Measured pressure in mm. water. L = Lymphocytes. P = Polymorphs.

The cerebro-spinal fluid pressure was accurately determined in only three cases (Nos. 12, 17, and 18) and it was significantly raised in the last two cases.

The remaining cases were determined by the very rough clinical test of rate of flow of the fluid from the lumbar puncture needle.

The colour of the fluid was uniformly clear, but the fluid in Case 10 showed a fine web-like clot resembling that occurring in tuberculous meningitis.

Cell counts were carried out in seven cases. All showed that lymphocytes were the predominating cells, though Case 18 on two occasions showed a large percentage of polymorphonuclear cells on each occasion. The other cells in this particular case were composed largely of lymphocytes.

The sodium chloride was appreciably lowered in five of the cases, in Cases 7 and 8 being particularly low. Both these cases clinically had meningeal involvement.

The protein was increased in eight cases, greatly so in Case 12, which clinically was a transverse myelitis, and in Case 18.

The sugar varied within the widest limits of normality.

Colloidal gold tests were performed in eight cases. This test revealed nothing significant, save in Case 12 where a result suggestive of a paretic curve was evident. Subsequent examination of the Wassermann reactions of both blood and cerebro-spinal fluid were completely negative.

Morbid Anatomy and Histology :

Autopsies were performed in five of the eight cases. Detailed examinations were made of the brain and spinal cord in three of the cases, and of the brain only in two cases. Macroscopically, the meninges were congested in two instances, in the others appearing healthy. The convolutions of the cerebral hemispheres were flattened in three cases, and in these instances the surface vessels were congested. One brain externally appeared healthy, while the remaining one showed one or two small subarachnoid hæmorrhages over the occipital lobe. Cut sections of the brains showed congestion of the brain substance in all cases. The spinal cord appeared healthy in four cases, the fifth showing small capillary hæmorrhages on section of the cord.

The characteristic feature of post-vaccinal encephalomyelitis—perivascular demyelination—was present in all cases to a greater or lesser degree. This characteristic lesion was widespread, affecting both the white and grey matters, in the brains of Cases 1, 7, 10, and 18, while in Case 12 the lesion was present in the occipital white matter and midbrain only, and there the pathological changes were mild and trifling. The spinal cords of Cases 7, 12, and 18 were severely affected, the thoracic portion in Cases 7 and 12, and the lumbar portion in Case 18 ; the cord was not examined in the remaining two cases.

Associated with this typical lesion of demyelination, there was also present in all the cases perivascular, especially perivenular and pericapillary rather than periarteriolar, collections of lymphocytes and monocytes with infiltration of the adjacent damaged parenchyma by mononuclear cells. These mononuclear cells were usually of the large type. In addition, in these damaged areas there were to be found reacting microglial cells. The vascular endothelium cells were swollen in Case 1, but this was not noted in any of the other cases. The nerve cells in the affected areas were, speaking generally, only slightly affected.

A detailed description of the neuropathological findings in each case will be found in Appendix III.

There were few other pathological features of note found in the ordinary autopsy examinations. No significant changes were found in the glands of the

side corresponding to the site of vaccination. None of the cases showed any abnormality at the sites of vaccination.

A large bedsore was present over the whole sacral area in Case 12. Case 1 showed a large hæmorrhagic area in the left lower lobe and similar smaller ones in the right upper lobe of the lungs. Case 10 showed a patchy pneumonic consolidation, and Case 18 showed bilateral pulmonary congestion, with an old adherent pleurisy on the right side. Hydrothorax present on both sides was found in Case 12, which also had oedema of the lungs. Bilateral early pyelonephritis was present in this case also.

Treatment :

Treatment was mainly symptomatic for all cases. Lumbar puncture was not carried out as a therapeutic measure in any case but only diagnostically.

Specific treatment with serum or blood from recently vaccinated persons was given in six cases, but the results were extremely disappointing. Three cases received whole blood from a recently vaccinated individual, given by intramuscular injection when they were admitted to hospital. The results from this form of therapy were unconvincing, no improvement, even temporary, being noted in any of the three cases, two of which ultimately succumbed.

Post-vaccinial serum was administered to three cases. In Case 17, 20 c.cm. were given intramuscularly, but the patient was already showing signs of recovery before administration of the serum. Case 12 had 20 c.cm. intravenously on admission with no benefit, while Case 18 had two injections intravenously of serum, also without benefit. Both Cases 12 and 18 had, in addition, courses of sulphonamides. Case 12 had two courses, the first of sulphathiazole and the second of sulphapyridine, both given to control an obstinate urinary infection. Case 18 had a course of sulphapyridine to control a pneumonic infection. In neither case did the sulphonamide appear to have any beneficial effect on the nervous disease. Case 8 had one gramme of sulphapyridine four-hourly for three days prior to admission to hospital, but the result of this therapy was doubtful, as the child was still critically ill on admission, though he recovered in a few days' time.

After Histories :

All the patients who recovered from the acute nervous manifestations following vaccination appeared to make complete recoveries. Residua were not encountered in any of the cases so far as can be ascertained up to the present.

Commentary :

It is not proposed to deal exhaustively with the literature which has appeared on post-vaccinal nervous disease, for the Reports of the Vaccination Committees (1928 and 1930) deal very fully with the problems presented by this disease. Reference to more recent work will, however, be made.

Ledingham (1934) has emphasised mass vaccination as being associated in time and place with an incidence of post-vaccinal nervous disease, and both Reports (1928 and 1930) comment on the frequency with which the disease occurs in school children, adolescents, and young adults, who are vaccinated for the first time at these age periods. The conditions existing in Edinburgh demonstrated the truth of these assertions. There existed in the City a severe form of smallpox and previously unvaccinated persons belonging to the age periods *supra* figured large among those seeking protective inoculation at the

public clinics. Primary vaccinations (all ages) accounted for about 22·3 per cent. of the total number vaccinated between November – December 1942. The Registrar-General has demonstrated in his Reports over a considerable period a progressive increase in the percentage of infants remaining unvaccinated on attaining the statutory age of six months. In his latest pre-war Report (1938) he records that only some 44·5 per cent. of the infants born in 1937 had been vaccinated on reaching the age of six months, although in 1941 he shows that the figure had risen to 55·59 per cent.

Twenty of the twenty-three cases under review had not been previously vaccinated, though one case is said to have been unsuccessfully vaccinated in infancy. This case is included among the twenty primarily vaccinated cases who developed the disease. Eighteen of these twenty cases were scholars, aged between 5 years and 16 years, while the remaining two cases were aged 2¼ years and 28 years respectively. This major incidence among school children is in accordance with the usual incidence of the disease. No cases of the disease were encountered in infants under one year, though the disease is not unknown at this age (Report, 1928 ; Scott, 1930 ; C.M.O. Reports, 1936, 1937, and 1938 ; Banerjea, 1937 ; Stiefler, 1938 ; Nicol, 1938 ; Sakoschansky and Trenchard, 1939 ; Burton and Weir, 1941).

The remaining three cases had each been successfully vaccinated on a previous occasion—infancy in each case. The ages of these patients were 26, 30, and 51 years respectively, the last of these cases being Case 12. The occurrence of the disease after previous successful vaccination is rare. Very few cases have been recorded (Report, 1928 ; 1930 ; Hekman, 1929 ; Mader, 1929 ; C.M.O. Report, 1938 ; Querido, 1930 ; Bergman, 1933 ; Giraud *et al*, 1938). Kaiser and Zappert (1938) encountered only one case following revaccination among 240 genuine cases of post-vaccinal disease of the nervous system occurring in Austria.

In the present series of cases, sixteen were females and seven were males.

Only one example of a familial incidence occurred among the twenty-three cases. A brother and sister (Cases 5 and 7) were affected, the brother succumbing to a severe encephalitic process, while the sister recovered from a sharp, severe illness.

The incubation periods of the disease as demonstrated by our cases showed that among the twenty primarily vaccinated persons seventeen had an incubation period of between seven and fourteen days, seven cases showing symptoms of the disease on the eleventh day after vaccination. This range of seven to fourteen days is the most usual period of the so-called incubation period (Reports, 1928 and 1930). The remaining three cases of this group of twenty individuals had longer incubation periods, viz., twenty, twenty, and twenty-two days respectively. Prolonged periods of incubation have been recorded by Banerjea (1935 and 1937).

The three previously vaccinated persons had incubation periods of three, five, and twelve days respectively. The very short incubation periods of the disease in two of these cases is of some interest, although such a short period from vaccination to the onset of nervous phenomena has been reported by others (*e.g.*, Hekman, 1929 ; Mader, 1929 ; Querido, 1930). A short incubation period is not, however, confined to post-vaccinal nervous disease occurring in previously successfully vaccinated persons, but may occur in primarily successfully vaccinated persons (*e.g.*, Miller, 1931).

Clinically, the symptoms presented by these twenty-three cases in this series—drowsiness, headache, pyrexia, vomiting, mental irritability, and pains in various parts of the body, such as the back, neck, and abdomen, were fairly uniform. One case gave a clinical picture of a pure myelitis, but post-mortem examination showed slight changes also in the occipital lobes and midbrain. Cases of transverse myelitis following vaccination have been recorded from time to time, *e.g.*, Peake (1929) ; Brockbank (1931) ; Clouston and Quinn (1937) ; Magé (1937) ; and Anderson and Mackenzie (1942). Doubtful cases were recorded by Perritt and Carrell (1930), and Olsen and Abbott (1937).

Our cases, therefore, fell into two groups on a clinical classification, viz., an encephalitic type, often associated with signs of meningeal irritation, and a transverse myelitic type. Other forms which the disease may take have been described by Glanzmann (1927) and Zappert (1930).

Although the symptoms presented by our patients were fairly uniform, there were one or two which were rather unusual and deserve some comment.

Five patients had convulsions, in one case the convulsions took the form akin to a status epilepticus. All these five patients made complete recoveries.

Although a varying degree of coma was present in most of the cases, two of them showed prolonged periods of coma ; in one case the coma lasted three days, and in the other it lasted four days. This serves to demonstrate how difficult it is to prognosticate on cases which appear gravely ill (Anderson and Mackenzie, 1942) since both these cases recovered.

Four cases complained of abdominal pain, and no evident reason to account for the pain was made out on clinical examination. Similar instances of abdominal pain are cited in Report (1928).

Photophobia was present in two cases, and dimness of vision in another. In a fourth case there was some slight papilloedema on admission to hospital, but this was completely recovered from. Amaurosis with optic neuritis has been described by Scheyhing (1939), von Herrenschwand (1939), while Breiger (1925) described occlusion of the central retinal artery following vaccination in a twenty-months' old infant, but the relationship of the vaccination to the occlusion was doubtful. A most interesting feature of our cases was the entire absence of squint.

Trismus was present in two cases. This symptom has been noted and emphasised by others, *e.g.*, Report (1928) ; Wiersma (1929) ; Wilson and Ford (1927). When present, it may give rise to a suspicion of tetanus.

Persistent hiccough, lasting some twelve hours, was a notable symptom in one case, and scant reference is to be found in the literature to any previous cases, though Lapage (1933) described a case with a curious semi-suppressed " hiccough."

Generalised tetanic spasms of the whole body occurred in two of our cases, in one the spasms being occasioned merely by ordinary clinical examination, while the other case had the spasms precipitated by lumbar puncture.

A slight degree of torticollis was present in one case, a feature noted by Sakoschansky and Trenchard (1939) in their case.

Pathological Anatomy :

The pathological anatomy of post-vaccinal nervous disease is of considerable interest in that the pathological picture, presented by cases dying from the disease, is very closely allied to other diseases in which demyelination is

the outstanding feature. These other diseases include smallpox, measles, anti-rabies treatment, chickenpox, and probably influenza. Further, the histological appearances of the central nervous system from cases of post-vaccinal disease closely resemble those present in disseminated sclerosis, Schilder's disease and disseminated encephalomyelitis. Marsden and Hurst (1932) have suggested the name "Acute Perivascular Myelinoclasis" for the changes present in the nervous system associated with the exanthemata such as vaccination, smallpox, and measles. The histological details shown by our patients conformed in all essential details with those described in Report (1928). Putnam (1941) drew attention to the importance of intravascular clotting of the blood producing thrombosis in some of the vessels of the nervous system. In this connection, our cases did not demonstrate the constant presence of thrombosed vessels. In fact, thrombosis was not a constant or prominent feature of the cases under review.

Aetiology :

The aetiology of the disease still remains obscure. It is not the purpose of this report to discuss the theories of causation of the disease in any detail. Nevertheless, brief mention may be made of the prevailing views of the aetiology. The Report (1928) considered three possible causes of the disease. Summarised, these views were : (1) that the disease is due to cerebral vaccinia, (2) that its occurrence after vaccination was merely fortuitous, (3) that it was due to a combined action of vaccine virus and some other neurotropic virus—an activation of the neurotropic virus by the vaccinia virus. The Report (1928) decided strongly in favour of this third possibility, as did Ledingham (1934) and others.

As a matter of interest, the notifications of verified cases of acute anterior poliomyelitis and encephalitis lethargica occurring in each month for the past two years in Edinburgh are shown below. They do not demonstrate any increase of incidence of these diseases during the period either of smallpox or of intensive vaccination in Edinburgh.

TABLE XXV.

Acute Anterior Poliomyelitis

Year	Jan.	Feb.	Mar.	Apr.	May	June	July	Aug.	Sep.	Oct.	Nov.	Dec.	Total
1940	–	–	1	–	–	–	2	–	4	2	1	4	14
1941	5	1	1	2	1	2	2	1	3	9	1	–	28
1942	–	–	–	1	1	1	3	1	1	1	1	2	12

Encephalitis Lethargica

Year	Jan.	Feb.	Mar.	Apr.	May	June	July	Aug.	Sep.	Oct.	Nov.	Dec.	Total
1940	–	–	1	–	–	–	–	–	1	1	4	–	7
1941	3	1	2	–	–	–	–	1	–	–	2	2	11
1942	–	–	–	–	–	–	–	1	1	1	–	3	6

In a minority report contained in the Report (1928), M'Intosh maintained that the disease was due solely to the vaccinia virus itself, a view which is supported by Hekman (1930), Brouwer (1939), and others, and now the theory generally held to be the most probable one. Support to this theory is lent by Report (1930), which records successful treatment of the disease by anti-

vaccinial serum. Though published results on the isolation of the vaccinia virus from the nervous system in cases of post-vaccinal nervous disease are scanty, M'Intosh and Blaxall (Report, 1928) recorded the presence of vaccinia virus in the brains of three cases, though this occurs in cases not dying from post-vaccinal encephalitis. A recent case of interest on this question has been recorded by Shaffer, Rake, and Hodes (1942) who successfully isolated the virus of measles from the brain of a patient who died from post-measles encephalitis—a condition closely allied to the post-vaccinal variety of encephalomyelitis. Hassin and Geiger (1930) suggest that the disease is due to a toxin supplied by the vaccine virus itself.

Turnbull (1928) and Coyle and Hurst (1929) examined the brachial plexuses of patients dying from post-vaccinal nervous disease, in an attempt to determine whether there was any evidence that these nerves acted as paths of infection of the virus or toxin from the vaccination sites. These examinations were all negative, save in one of Turnbull's cases, where he found an inflammatory reaction extending from the site of vaccination to the distal end of the left brachial plexus. Coyle and Hurst, in their case, examined the intervertebral ganglia in addition to the brachial plexus, but they could find no evidence of any lesion in them. These workers also found that pathological changes in the spinal cord were almost completely limited to the right half of the cord while their patient had been vaccinated on the left arm.

Hutter (1930) stressed the constitutional aspect of the disease, and Finley (1938) presented evidence in an attempt to prove that the encephalitis following vaccination, smallpox, and measles, etc., was allergic in nature. M'Clure (1930) recorded a case of urticarial eruption following vaccination in a patient who subsequently developed post-vaccinal encephalitis. Putnam (1941) strongly supported the allergic theory.

Sequelae :

None of our cases, up to the present, have shown any evidence of sequelae, though these are, of course, not unknown. The first case of post-vaccinal nervous disease recorded (Comby, quoted by Report, 1928) showed various sequelae such as clumsy and rigid limbs, exaggerated reflexes, a squint, and changes mentally, such as fits of anger. Other sequelae have been reported, *e.g.*, mental dullness, poor memory (Report, 1928) ; hemiparesis (Report, 1928 ; Weischel, 1931) ; spastic hemiplegia (Duken, 1930) ; epileptic convulsions (Linde, 1936) ; diabetes insipidus (Roehm, 1932).

A case of extreme importance has recently been described by Herkenrath (1935). This is probably the only case in the literature of a pathological study of post-vaccinal encephalomyelitis in which recovery from that disease took place, the neuropathological examination taking place some time later after the patient had died from another condition. The recovery from the encephalomyelitis had been almost complete, save for an inability to speak as clearly as the patient—a girl of three years—had done before. About thirty-four months after discharge from hospital after her encephalomyelitis, the child died from an infection of another nature. A post-mortem was conducted and the following is a brief summary of the pathological findings : Grossly, the brain was healthy. Microscopically, some fat-laden "scavenger" cells were found in the perivascular spaces of the cerebellum, pons, medulla, and spinal cord. These fat-laden cells were small, round, and were most

noticeably present in the peri-venular sheaths. There was no evidence of perivascular, sub-ependymal, or cortical gliosis, the myelin sheaths were normal, and the ganglion cells were healthy for the most part, though Herkenrath noted an atrophy of the cells of the right lateral nucleus of the thalamus and in the temporal lobe. In these areas, there was a slight increase of small glial cells. Herkenrath did not consider these changes in the thalamus or temporal lobes as due to the original encephalitic process and regarded the presence of the perivascular scavenger cells as the only pathological evidence of this disease. It would appear from this report, at least, that recovery, when it does occur, is likely to be complete, though, of course, this is not always so.

Follow-up examinations have been carried out by Kudelka (1932) and by Kaiser and Zappert (1937). Kudelka re-examined sixteen girls and eleven boys in whom post-vaccinal nervous disease developed during the period 1928-31. His findings were entirely favourable, only one case showing gross abnormality—a unilateral paralysis of the sixth cranial nerve. Kaiser and Zappert in their important contribution published the results of their follow-up of one hundred and ten children who had suffered from post-vaccinal encephalomyelitis. The only serious sequela in these one hundred and ten children was a spastic gait in one patient. A few others had changes in their reflexes, urinary disturbances of a minor character, and mild facial paralysis. In none of them, however, were the sequelae serious, nor did they interfere with the normal activities of the patients.

Treatment :

The treatment of post-vaccinal encephalomyelitis is mainly symptomatic, though as a result of the published reports of Horder (1929), Hekman (1929), Roper (1933), and others, the use of serum from persons recently successfully vaccinated may give beneficial results and should always be tried in the more serious cases. Our own experiences with serum therapy were, however, not encouraging. More recently, Bürton and Weir (1941) have reported success in one case of post-vaccinal encephalomyelitis with the combined use of blood transfusions from recently vaccinated individuals and sulphapyridine, and they suggest further trials of this therapy. Sulphonamides were used in three of our cases in this series, but the results were far from conclusive, two of the patients dying from intercurrent infections which the sulphonamides failed to control. Where convulsions are present, Davidson and Thomas (1942) have reported success with combined intravenous convalescent serum and pentothal sodium. There seem, however, to be no very clear opinions on the values of the various forms of therapy. In general, it may be said that therapy is mainly symptomatic, though the use of serum should be tried in all cases, whether apparently mild or severe.

DISINFECTION OF PROPERTY AND FURNISHINGS DURING OUTBREAK

Formaldehyde was the disinfectant used throughout the epidemic. The efficacy of formaldehyde as a general disinfectant has now been established for many years (Kolle and Wassermann, 1931 ; Fust, 1941). The question

of toxicity was important, as it was impracticable to clear the whole property of the various families during the disinfection when only one house was involved. It was known that formalin could be used with reasonable safety to the operators, provided that full precautions were taken, and it was known from long experience that formalin would do the minimum of damage to the wide range of materials which it would be necessary to treat.

Procedure of Disinfection : When the patient had been removed to hospital from a private dwelling-house, the family contacts were taken to the reception house for observation. The dwelling-house was then locked, the keys being kept at the Public Health Department, and all passages, stairs, and, in particular, the sanitary accommodation used in common with the other tenants, were disinfected immediately. In most instances the infected house was also disinfected on the day of the occupant's removal or early on the following day, relatives from the reception house being available to indicate valuables likely to be damaged by the disinfecting process or to indicate materials which could be destroyed. In the few instances, however, where the patient was the sole occupier the detailed disinfection was left until the patient's discharge from hospital. In these latter instances, the patient was accommodated temporarily in the reception house during the disinfection.

All material the owners consented to have destroyed was packed in sacks ready for destruction. Thereafter the material was taken to the disinfecting station and burned as soon as permission had been obtained. Ration books and paper money handled by patients were destroyed after arrangements had been made for their replacement. All bedding, mattresses, curtains, carpets, rugs, and all clothing and cloth were packed in large sacks made of closely-woven material. The outsides of the sacks were sprayed with formalin before removal from the infected place, and they were then carried to the waiting van for removal to the central station for disinfection by means of steam.

Coins, trinkets, and jewellery were placed in boiling formalin before returning them to the owners, but watches and bedside clocks were sprayed in a special chamber, and were then opened and exposed to maximum concentration of formaldehyde gas for twenty-four hours, the owner being warned to have them cleaned as soon as possible to avoid damage by damp. This chamber was also used for treatment of valuable books, but in this case the spraying was confined to the walls of the chamber, followed by exposure to maximum concentration of formaldehyde gas as above.

Before starting the fumigation process, all drawers, presses and cupboards were searched for powerful oxidising agents, such as potassium permanganate and bleaching powder, as experiments showed that these substances react with the vapour and spray in the room and under experimental conditions had caused an outbreak of fire.

The spraying was carried out in one room at a time, and in the interests of safety it was arranged that there were always two operators present in the room. As a high concentration of formaldehyde gas was achieved, and as the thorough spraying of a room was found to take considerable time, it was necessary to use breathing apparatus obtaining its air from the outside; the limit of endurance under these conditions, using the breathing apparatus and doing the work of moving the furniture and operating the pumps, was found to be about three hours.

In the dwelling-houses where the areas to be covered were small, the spraying was done with half-gallon hand sprayers, but in hospital wards stirrup pumps were used, and high ceilings were reached by tying the spraying nozzles to broom handles. In addition to Remote Breathing Apparatus, the operators were protected by heavy rubber gloves, boots, and rubber aprons, as a certain amount of splashing was inevitable in the handling of pumps and long lines of hose. The room remote from the main door was chosen for a starting-point, the walls, floors, and roof being sprayed in turn. A one in eight dilution of formalin in hot water was used at this stage, the purpose being to soak all surfaces with a liquid of a lethal strength and to raise the moisture content of the air to as near saturation point as possible.

During this spraying a considerable quantity of formaldehyde gas was liberated from the liquid, this liberation being rapid because a hot solution of the gas was being sprayed. All furniture which because of its size could not be removed from the scene of operations, was moved back from the wall, which, together with the floor and the backs of the furniture, were sprayed before the latter was replaced. Sufficient solution was used to soak all these surfaces, and the quantity of liquid required was found to vary greatly with the type of surface.

In the average house it was unsafe to leave free liquid lying on the floors as it soaked through and appeared in the premises below, so when walls or furniture were of such a nature that a large quantity of liquid was used during the treatment, the excess liquid was mopped up when the spraying stage was completed. The vapours in the room were then brought to maximum strength by generation of formaldehyde gas by the following method : A four-gallon pail was placed inside a similar pail containing water. One pound weight of potassium permanganate was placed in the first pail, and when all was ready one pint of formalin (40 per cent. formaldehyde) was tipped into the permanganate.

These proportions were used as they give the maximum yield of gas (M'Culloch, 1936). Formaldehyde gas and steam evolved, and as these vapours may be explosive the mixture was sprayed with water when the reaction became violent ; this also prevents boiling over. When the generation of gas had ceased the pail was again sprayed with water to prevent violent local reaction, which can take place on the sides and rim of the pail after the main reaction has subsided and which may be accompanied by flame.

The amount of gas liberated was varied to suit the type of building, as the construction of some rooms made adequate sealing impossible, but the strength aimed at was that given by the liberation of the formaldehyde in one pint of formalin for every 1000 cubic feet. The room was then closed and sealed as far as practicable and left locked for six hours. Full precautions were observed during the opening up of the house, and it was left for twenty-four hours with all the windows open. All free liquid was then mopped up and the roof, walls, and floors were swept. Even at this stage experience showed that a period of some days might be necessary before the house was fit for comfortable habitation.

Bacteriological Control of Disinfection : As no record could be found of the efficacy of formaldehyde as a proved disinfectant of smallpox infected rooms when used under actual working conditions in the concentration,

temperature, and humidity that could be maintained in an ordinary building, it was decided to attempt to control the process by testing its bactericidal action on a series of known organisms including the vaccinia virus, exposed in various positions during the actual disinfection process. In this connection it may be stated that control of the fumigation process by estimation of the actual concentration of formaldehyde vapour in the air of the room is not at present practicable.

The effect of temperature on the bactericidal action of formaldehyde is known to be of great importance. For example, M'Culloch states that nearly ten times as great a concentration is required to kill *E. typhi* at 2° C. as at 40° C. On this account the following observations were taken during the bacteriological investigations to show that the process was efficient at temperatures encountered during winter in this City. The relative humidity was calculated from observations of the wet and dry bulb thermometer, and although the thermometers were swung to ensure the necessary air movement, it was not always possible to do this for sufficient time to permit the temperature to reach equilibrium, as the readings were taken during actual disinfection. The calculated relative humidities are therefore approximate and mainly of comparative value. Table XXVI. shows the temperature and humidity readings taken during disinfection processes.

TABLE XXVI.

In all the cases shown below *B. subtilis* was killed.

Description of Premises		Outside Temp. F°	Inside Temp. F°	R. H. %
Unheated room in which a hot solution was sprayed by hand appliances.	Before spraying . . .	36	41	77
	After generation of gas .	–	42	85
Unheated room in which a hot solution was sprayed by hand appliances.	Before spraying . . .	44	45·5	87
	After generation of gas .	–	46	86
Heated ward in which a hot solution was sprayed by means of a stirrup pump.	Before spraying . . .	45	55	81
	After generation of gas .	–	59	94
Heated ward in which a hot solution was sprayed by stirrup pump along with spraying by means of a steam jet.	Before spraying . . .	38·5	47	79
	After generation of gas .	–	53	93

The following bacteriological investigations were carried out in the Bacteriology Department of the University of Edinburgh by the staff associated with the Bacteriological Services of the City of Edinburgh :—

BACTERIOLOGICAL OBSERVATIONS ON THE EFFICACY OF THE FORMALIN FUMIGATION PROCESS

Many workers have reported upon the disinfecting action of formaldehyde on bacteria (for references, see bibliography for section on Disinfection),

but not under conditions exactly comparable with those prevailing during fumigation of smallpox infected premises. It was decided to investigate the possibility of obtaining some control and idea of the efficacy of the fumigation process by studying its action on different micro-organisms.

Bacteriological Methods : The organisms used were *Staphylococcus aureus*, *Corynebacterium diphtheriae*, *Streptococcus pyogenes*, *Bacillus subtilis*, *Mycobacterium tuberculosis*, all freshly isolated strains except *B. subtilis*, and ground-up crusts from vaccinated rabbits. The bacteria were cultured for twenty-four hours (except *M. tuberculosis*, which was cultured for four weeks), and a loopful of a saline suspension of each organism was spread over two small sterile pieces of glass fixed in a Petri dish by means of a drop of melted agar. The vaccinia crusts were obtained by scraping the lesions from vaccinated rabbits the day before the experiment was carried out; the crusts so obtained were desiccated *in vacuo* over calcium chloride for eighteen hours, and, after grinding in a mortar, spread thinly in a Petri dish. These crusts were used in preference to crusts from humans in order to have material as potent as possible so that the subsequent animal experiments might yield results as definite as possible. In one experiment, suspensions of the above bacteria (except *M. tuberculosis*) were also inoculated on to the inside pages of paper-covered books, which were wrapped in brown paper and then exposed as described below.

These preparations were exposed in the rooms under investigation at varying times after the start of the generation of formaldehyde *vapour* and for varying lengths of time. Precautions were taken to prevent droplets from the atmosphere of the fumigated rooms falling directly on to the exposed plates or material. This precaution was considered necessary because there was no certainty that all the organisms in the room would come in contact with the droplets that deposited during the fumigation process. After exposure the pieces of glass were removed and the infected surfaces were smeared over suitable culture media and then left resting on the surface of the medium during the subsequent incubation. One of the glasses infected with the tubercle bacillus was cultured on Löwenstein-Jensen medium and the other was shaken up in broth which was then injected into each of two guinea-pigs. The vaccinia crusts were ground up in a small quantity of broth, and the suspension heavily inoculated on to the scarified skin of a rabbit.

The cultures were examined at intervals during forty-eight hours' incubation (*i.e.*, prolonged incubation was not carried out), except in the case of cultures of *M. tuberculosis* which were examined during six weeks' incubation. The injected guinea-pigs were killed and examined after six weeks, and the vaccinated rabbits were examined daily.

Discussion of Results : In a preliminary experiment the test organisms (after exposure to the fumigation process for the full six hours) gave negative results on cultivation or on animal inoculation. Further experiments were then carried out with the plates protected, as described above, and using varying degrees of exposure to the disinfecting vapour. On the occasions when it was necessary to enter or leave the room in order to place or withdraw the organisms for exposure, the operator was suitably protected (Remote Breathing Apparatus, etc.) and the operations were carried out with the minimum

of disturbance. The test agents detailed above were included in each experiment, with the exception of the vaccinia crusts and the tubercle bacillus which were included in two and in one of the experiments respectively. The main results are summarised in the following table (Table XXVII.) :—

TABLE XXVII.

Showing the effect on the viability of organisms exposed for varying periods of time in the room which was being fumigated.

(The exposures were made at different times from the start of the generation of formaldehyde vapour : see column headed " When Exposure was made.")

Organisms	When Exposure was made	Duration of Exposure	Results of Incubation
Staph. aureus *Str. pyogenes* *C. diphtheriae*	50 mins.	10 mins. (2)*	No growth
	60 mins.	5 mins.	No growth
	1½–2 hrs.	10 mins. (2)	No growth
	2¾ hrs.	5 mins.	No growth
	4½ hrs.	5 mins.	No growth
	6 hrs.	10 mins.	No growth
	6 hrs.	15 mins.	No growth
B. subtilis	50 mins.	10 mins. (2)	No growth
	60 mins.	5 mins.	No growth
	1½–2 hrs.	10 mins. (2)	Growth
	2¾ hrs.	5 mins.	Growth
	4½ hrs.	5 mins.	Growth
	6 hrs.	10 mins.	Growth
	6 hrs.	15 mins.	Growth
M. tuberculosis	0–5 mins.	30 mins.	No growth†
	2¾ hrs.	10 mins.	No growth†
	4½ hrs.	10 mins.	Growth‡
			Result of Animal Vaccination
Vaccinia	50 mins.	10 mins.	No reaction.
	60 mins.	5 mins.	Doubtful or weak reaction.
	1½ hrs.	2 hrs.	No reaction.
	2¾ hrs.	5 mins.	Positive reaction, *i.e.*, virus not inactivated.
	3 hrs. 20 mins.	3 hrs.	No reaction.
	4½ hrs.	5 mins.	Positive reaction, *i.e.*, virus not inactivated.
	6 hrs.	10 mins.	Weak positive reaction.

* Figures in brackets refer to the number of exposures of each organism ; *e.g.*, the figure (2)* means that each of the organisms, *Staph. aureus*, *Str. pyogenes* and *C. diphtheriae* were exposed for 10 minutes in each of two experiments.

† Results supported by animal inoculation which was negative.

‡ Each of the two guinea-pigs inoculated developed tuberculous lesions.

In each experiment control tests were carried out with each organism, the control organisms being treated in the same way as the exposed organisms, but not subjected to the fumigation process. The results of the different experiments were consistent, and show that the organisms *Str. pyogenes*, *Staph. aureus*, and *C. diphtheriae* were rapidly killed (as judged by an incubation

period of forty-eight hours) under the conditions of the experiment. The sporing aerobe *B. subtilis* and the virus in the vaccinia crusts were relatively resistant; however, the bacillus was killed and the virus inactivated by an exposure of only ten minutes made fifty minutes after the start of the generation of the formaldehyde vapour. It is of interest to note that the resistant organism *M. tuberculosis* was killed, and the time required was only ten minutes when an exposure was made $2\frac{3}{4}$ hours after generation of the formaldehyde vapour. In the experiment in which the paper-covered books were used, the exposure was of six hours' duration, and subsequent tests showed that the non-sporing organisms were killed; *B. subtilis*, however, showed growth after forty-eight hours, but not after twenty-four hours' incubation.

Application of tests similar to those described above would appear to be of some value as a check upon the efficiency of fumigation of smallpox infected rooms with formaldehyde. For example, the conditions required to kill *B. subtilis* were somewhat similar to those required to inactivate the vaccinia crusts, and a *partial* check of the efficiency of the fumigation process might, therefore, be obtained by exposing *B. subtilis* in the rooms which are being fumigated. For example, these results show that *B. subtilis* was killed during a five-minute exposure made one hour after generation of the vapour. If this is found to be the case, it may be regarded as *partial* evidence of the effectiveness of a fumigation process.

It should be pointed out that only one strain of each of these organisms has been used, and, in any case, it is not suggested that destruction of *B. subtilis* under the conditions described above would provide proof of inactivation of the vaccinia crusts. However, it should be borne in mind that the organisms present in the room would be exposed to the action of the disinfection process *not merely for a few minutes but for six hours*, i.e., *there is a wide margin of safety.*

In these experiments bacteria in saline suspension have been exposed to the fumigation process. It is realised that different results would probably have been obtained if the bacteria had been incorporated in a suspension of sputum or otherwise associated with organic matter. This aspect does not lie within the scope of the present work, the object of which was to investigate the disinfecting action of formaldehyde (as used in the routine disinfection of smallpox-infected premises) on certain organisms including the vaccinia virus, and thereby to provide some indication of the efficiency of such routine disinfection processes. It was hoped that the results might suggest some simple bacteriological test of the efficiency of such a fumigation process with regard to inactivation of the variola virus itself (*vide infra*).

The variola virus itself has not been used in these experiments owing to the difficulties and dangers associated with the necessary animal experiments. Furthermore, for reasons already stated, the source of the vaccinia virus used was rabbit (*i.e.*, not human) crusts. In consideration, however, of the close relationship and similarity of properties of viruses obtainable from variola and vaccinia respectively, it is reasonable to infer that the resistance of each would be of the same order.

The results show that this formalin fumigation process may be of considerable practical value, not only against smallpox virus, but also against other organisms, *e.g.*, for disinfection of wards or other premises infected with *Str. pyogenes*, *C. diphtheriae*, *M. tuberculosis*, etc.

BIBLIOGRAPHY

General :

Ricketts, T. F., and Byles, J. B., 1908, " Diagnosis of Smallpox," Cassells, London.

Epidemiological Aspects :

Francis, T., Magill, T. P., Rickard, E. R., and Beck, M. D., 1937, *Journ. Am. Med. Ass.*, 109, 566.

Fyfe, G. M., 1943, *Health Bulletin* (Dept. of Health for Scot.), 2, 30.

Macgregor, A. S. M., and Peters, R. J., 1942, *Brit. Med. Journ.*, 2, 627.

Pettit, H., Mudd, S., and Pepper, D. S., 1936, *Am. Journ. Pub. Hlth.*, 27, 1141.

Piszczek, E. A., Shaughnessy, H. J., Zichis, J., and Levinson, S. O., 1941, *Journ. Am. Med. Ass.*, 117, 1962.

Reports, Annual, 1892-1942, Medical Officer of Health, Edinburgh.

Top, F. H., and Vaughan, H. F., 1941, *Am. Journ. Pub. Hlth.*, 31, 777.

Clinical Account of Outbreak :

Glen, J., 1943, *Proc. R. Soc. Med.*, 36, 234.

Vaccination and Its Sequelae : Post-Vaccinial Eruptions :

Bloch, E., 1942, *Lancet*, 2, 504.

Chalke, H. D., 1931, *Ibid.*, 1, 578.

Davidson, L. S. P., and Davis, L. J., 1943, *Ibid.*, 2, 103.

Jubb, A. A., 1943, *Brit. Med. Journ.*, 1, 91.

Shaw, W. V., and Stevenson, W. D. H., 1939, " British Encyclopaedia of Medical Practice," 12, Article " Vaccination."

van Rooyen, C. E., and Rhodes, A. J., 1940, " Virus Diseases of Man," Oxford University Press.

Post-Vaccinal Encephalitis :

Anderson, T., and M'Kenzie, P., 1942, *Lancet*, 2, 667.

Banerjea, J. C., 1935, *Indian Journ. Pediat.*, 2, 177 ; *ibid.*, 1937, 4, 91.

Bergman, G., 1932, *Hygeia*, p. 673, quoted *Brit. Med. Journ.*, 1933, 1, No. 73, in Epitome.

Breiger, E., 1925, *Ztschr. f. Augenh.*, 58, 407.

Brockbank, T. W., 1931, *Journ. Amer. Med. Ass.*, 97, 227.

Brouwer, B., 1939, *Maandschr. v. Kindergeneesk*, 8, 379.

Burton, A. H. G., and Weir, J. H., 1941, *Lancet*, 2, 561.

Clouston, T. M., and Quin, B. H., 1937, *Med. Journ. Aust.*, 2, 568.

Coyle, C. D., and Hurst, E. W., 1929, *Lancet*, 2, 1246.

Davidson, C. L., and Thomas, J. T., 1942, *Arch. Dis. Childh.*, 17, 162.
Duken, J., 1930, *Ztschr. f. Kinderh.*, 50, 292.
Finley, K. H., 1938, *Arch. Neurol. and Psychiat.*, 39, 1047.
Glanzmann, E., 1927, *Schweiz. med. Wschr.*, 57, 145.
Giraud, P., Bernard, R., and Provensal, J., 1938, *Marseille-Med.*, 75, 585.
Hassin, G. B., and Geiger, J. C., 1930, *Arch. Neurol. and Psychiat.*, 23, 481.
Hekman, J., 1929, *Nederl. tijdschr.* v. *geneesk*, 2, 4774, 5227.
Hekman, J., 1930, quoted *Report of Vaccination Committee*, 1930.
Herkenrath, B., 1935, *Ztschr. f.d. ges. Neurol. u. Psychiat.*, 152, 293.
Herrenschwand, F. von, 1939, *Klin. Monatsbl. f. Augenh.*, 102, 815.
Horder, T., 1929, *Lancet*, 1, 1301.
Hutter, A., 1930, *Nederl. tijdschr.* v. *geneesk*, 1, 893.
Kaiser, M., and Zappert, J., 1937, *München med. Wchnschr.*, 84, 801.
Kaiser, M., and Zappert, J., 1938, *Die "Post-vaccinale Encephalitis" nach amtlichen osterreichischen Daten*, Springer, Wien.
Kudelka, O., 1932, *München med. Wchnschr.*, 79, 379.
Lapage, C. P., 1933, *Brit. Med. Journ.*, 1, 811.
Ledingham, J. C. G., 1934, *Proc. R. Soc. Med.* (Sect. Epidem. and State Med.), 27, 881.
Linde, E., 1936, *Svenska läk-tidning*, 33, 193.
Mader, A., 1929, *Jahrb. f. Kinderh.*, 123, 11.
Marsden, J. P., and Hurst, E. W., 1932, *Brain*, 55, 181.
Magé, J., 1937, *J. belge Neurol.*, 37, 703.
Miller, M. K., 1931, *Journ. Amer. Med. Ass.*, 97, 161.
Ministry of Health, Annual Reports of Chief Medical Officer, 1936, 1937, 1938.
M'Clure, W. B., 1930, *Chin. Med. Journ.*, 44, 526.
Nicol, C. M., 1938, *Bull. de l'Office Internat. d'Hyg., publ.* 30, 1708.
Olsen, C. W., and Abbott, K. H., 1937, *Bull. Los Angeles Neurol. Soc.*, 2, 34.
Peake, H. G., 1929, *Journ. Roy. Army Med. Corps*, 53, 443.
Perritt, R. A., and Carrell, R. C., 1930, *Journ. Amer. Med. Ass.*, 94, 793.
Putnam, T. J., 1941, *Bull. New York Acad. Med.*, 2nd series, 17, 337.
Querido, A., 1930, *Nederl. tijdschr.* v. *geneesk*, 1, 1047.
Registrar-General for Scotland, Annual Report, 1938.
Reports of the Committee on Vaccination, 1928, 1930.
Roehm, H. R., 1932, *Amer. Journ. Dis. Child.*, 44, 1293.
Roper, F. A., 1933, *Brit. Med. Journ.*, 2, 103.
Sakoschansky, E., and Trenchard, H. J., 1939, *Brit. Med. Journ.*, 1, 1229.
Scheyhing, H., 1939, *Klin. Monatsbl. f. Augenh.*, 102, 223.
Scott, J. F. M., 1930, *Brit. Journ. Child. Dis.*, 247.
Stiefler, G., 1938, *Wien. Klin. Woch.*, 51, 425.
Shaffer, M. F., Rake, G., and Hodes, H. L., 1942, *Amer. Journ. Dis. Child.*, 64, 815.

Turnbull, H. M., 1928, quoted in Vaccination Committee Report.
Weischel, M., 1931, *Monatsbl. f. Kinderh.*, 49, 28.
Wiersma, D., 1929, *Acta Psychiat. et Neurol.*, 4, 75.
Wilson, R. E., and Ford, F. R., 1927, *Bull. Johns Hopkins Hosp.*, 40, 337.
Zappert, J., 1930, quoted *Medical Annual*, 1932, p. 569.

Disinfection and Bacteriological Control of :

Fust, B., 1941, *Schweiz. med. Wochnschr.*, 71, 1425.
Kolle, W., and Wassermann, A. V., 1931, *Handbuch der Pathogenen Microorganismen*, 3rd ed., vol. 3, part 2.
M'Culloch, E. C., 1936, " Disinfection and Sterilization," Kimpton, London.

APPENDIX I.:

No.	Sex	Age	Occupation	Exposure				Date of Sickening
				City	Institution			
					Name	Date of Admission	Date of Discharge	
1	M.	46	Miner	–	R.I.E. (Wd. 13)	5/10/42	28/10/42	23/10/42
2	M.	12	Scholar	–	R.I.E. (Wd. 13)	15/10/42	29/10/42	29/10/42
3	M.	25	Polish Medical Student	–	R.I.E.	–	–	29/10/42
4	M.	34	Medical Student	–	R.I.E.	–	–	30/10/42
5	M.	16	Miner	–	Conv. Home	20/10/42	5/11/42	3/11/42
6	M.	29	Polish Medical Student	–	R.I.E.	–	–	2/11/42
7	M.	18	Grocer	–	R.I.E. (Wd. 7)	19/10/42	23/10/42	4/11/42
8	M.	39	Unemployed	–	Conv. Home	1/8/42	28/10/42	5/11/42
9	M.	37	Miner	–	Conv. Home	9/10/42	7/11/42	4/11/42
10	M.	26	Bank Messenger	–	Conv. Home	23/10/42	7/11/42	4/11/42
11	M.	42	Despatch Clerk	–	Conv. Home	14/10/42	29/10/42	4/11/42
12	M.	17	Joiner	–	Conv. Home	16/10/42	7/11/42	5/11/42
13	M.	9	Scholar	–	Conv. Home	20/10/42	7/11/42	5/11/42
14	M.	22	Professional Footballer	–	Conv. Home	13/10/42	4/11/42	5/11/42
15	M.	14	Scholar	–	Conv. Home	2/10/42	31/10/42	5/11/42
16	M.	38	Bolt Shifter	–	Conv. Home	13/10/42	4/11/42	4/11/42
17	M.	65	No Occupation	–	Conv. Home	16/10/42	2/11/42	5/11/42
18	M.	61	Miner	–	Conv. Home	20/10/42	31/10/42	7/11/42
19	M.	67	Oil Works Labourer	–	Conv. Home	13/10/42	29/10/42	7/11/42
20	M.	57	Iron Driller	–	Conv. Home	27/10/42	11/11/42	9/11/42
21	M.	68	Engineer (Retd.)	–	R.I.E. (Wd. 13)	13/10/42	12/11/42	8/11/42
22	F.	47	Kitchenmaid	–	R.I.E. (Wd. 13)	–	–	10/11/42
23	M.	79	Blacksmith (Retd.)	–	R.I.E. (Wd. 7)	27/10/42	4/11/42	10/11/42
24	F.	17	Clerkess	City	–	–	–	16/11/42
25	F.	24	Machinist	City	–	–	–	19/11/42
26	M.	28	Telephone Engineer	City	–	–	–	19/11/42
27	F.	18	Bakery Worker	City	–	–	–	20/11/42
28	F.	45	Clerkess	City	–	–	–	20/11/42
29	M.	35	Chemical Process Worker	City	–	–	–	22/11/42
30	F.	58	Housekeeper	City	–	–	–	26/11/42
31	F.	58	Housewife	City	–	–	–	6/12/42
32	M.	55	Brewery Worker	City	–	–	–	5/12/42
33	F.	9/12	–	City	–	–	–	6/12/42
34	F.	59	Chemical Worker	City	–	–	–	9/12/42
35	M.	55	Carter	City	–	–	–	13/12/42
36	F.	55	Housewife	City	–	–	–	28/12/42

* Calculated 14 days from appearance of rash.

TABLE OF CASES

Date of Rash	Admission to Smallpox Hospital	Probable Infection*	Discharge from Smallpox Hospital	Nature of Rash	Remarks
27/10/42	28/10/42	13/10/42	22/12/42	Semi-confluent	
31/10/42	1/11/42	17/10/42	Died 10/11/42	Confluent	
3/11/42	6/11/42	20/10/42	30/11/42	Discrete sparse	
3/11/42	6/11/42	20/10/42	30/11/42	Discrete sparse	
4/11/42	5/11/42	21/10/42	30/1/43	Confluent	
5/11/42	5/11/42	22/10/42	22/12/42	Discrete	
5/11/42	5/11/42	22/10/42	5/2/43	Confluent	
6/11/42	6/11/42	23/10/42	Died 16/11/42	Confluent	
7/11/42	7/11/42	24/10/42	27/11/42	Discrete sparse	
7/11/42	7/11/42	24/10/42	2/12/42	Discrete sparse	
7/11/42	–	24/10/42	Died 8/11/42	Hæmorrhagic	Died at home before admission to hospital
7/11/42	7/11/42	24/10/42	9/2/43	Confluent	
7/11/42	7/11/42	24/10/42	30/1/43	Discrete	
7/11/42	8/11/42†	24/10/42	Died 14/11/42	Hæmorrhagic	
7/11/42	10/11/42	24/10/42	5/2/43	Semi-confluent	
8/11/42	10/11/42	25/10/42	27/11/42	Discrete sparse	
9/11/42	11/11/42	26/10/42	5/12/42	Discrete sparse	
10/11/42	10/11/42	27/10/42	19/12/42	Discrete	
10/11/42	12/11/42	27/10/42	12/12/42	Discrete	
11/11/42	11/11/42	28/10/42	5/12/42	Discrete sparse	
11/11/42	12/11/42	28/10/42	27/3/43	Discrete	Contact Case 1
11/11/42	10/11/42	28/10/42	3/12/42	Discrete sparse	Wife and contact Case 1
12/11/42	12/11/42	29/10/42	1/3/43	Discrete sparse	
18/11/42	19/11/42	4/11/42	9/2/43	Discrete	
22/11/42	9/12/42	8/11/42	30/12/42	Discrete	
23/11/42	21/11/42†	9/11/42	15/12/42	Discrete	
23/11/42	26/11/42	9/11/42	30/1/43	Discrete	
23/11/42	25/11/42	9/11/42	3/2/43	Semi-confluent	
26/11/42	30/11/42	12/11/42	16/1/43	Discrete	
29/11/42	30/11/42	15/11/42	Died 5/12/42	Hæmorrhagic	
7/12/42	8/12/42	23/11/42	Died 9/12/42	Hæmorrhagic	Contact Case 25
8/12/42	9/12/42	24/11/42	30/12/42	Discrete sparse	Contact Case 25
8/12/42	8/12/42	24/11/42	16/1/43	Discrete	Contact Case 29
11/12/42	11/12/42	28/11/42	Died 18/12/42	Hæmorrhagic	Contact Case 29
15/12/42	18/12/42	1/12/42	Died 19/12/42	Hæmorrhagic	
29/12/42	30/12/42	15/12/42	23/1/43	Discrete sparse	Contact Case 35

† Admitted to hospitals outwith Edinburgh.

APPENDIX II.—Clinical Summary of Patients Treated in City Hospital

Case No.	Initials	Age	Sex	Date of onset of Initial Illness	Date of Rash	Date of Admission to Hospital	Type of Rash	Result	History and State of Vaccination	Remarks
1	J. W.	46	M.	23/10/42	27/10/42	28/10/42	Semi-confluent	Recovered	Infancy—2 scars. 1914–18—? scar	Illness of moderate severity. Efflorescence very gradual and distribution anomalous until 4th day of eruption. Monophasic development characteristic. Received sulphanilamide, 24 grams. Complications: a few small boils.
2	A. S.	12	M.	29/10/42	31/10/42	1/11/42	Confluent	Died 13th day of disease	Un-vaccinated	Admitted with signs of meningeal irritation and a few maculo-papules on chest and upper part of back. Distribution atypical in that patient died with lower forearms, hands, lower legs and feet almost free from rash though confluent on face, upper arms and chest, and pubis. Circulation had been failing for previous three days and there was no secondary fever. Received 33 grams sulphapyridine.
3	J. C.	25	M.	29/10/42	3/11/42	6/11/42	Discrete sparse	Recovered	Infancy and 1927—2 scars. Re-vaccinated 8/11/42—no take	Lesions in pustular stage on admission, *i.e.*, on 4th day of rash. According to history had quite sharp initial illness but no constitutional reaction after rash came out. Rash obviously modified numerically and in maturation.
4	D. T.	34	M.	30/10/42	3/11/42	6/11/42	Discrete sparse	Recovered	Infancy—1 scar. Re-vaccinated 7/11/42 and 11/11/42—no take	Lesions small in size, very sparse, and aborted at vesiculo-pustular stage in 6th day of rash. Initial illness well marked but constitutional disturbance disappeared when rash came out. One seed on right palm and three on soles. Nodule appeared in L. epididymis on 13th day of illness and disappeared in four days.

Case No.	Initials	Age	Sex	Date of onset of Initial Illness	Date of Rash	Date of Admission to Hospital	Type of Rash	Result	History and State of Vaccination	Remarks
5	T. D.	16	M.	3/11/42	4/11/42	5/11/42	Con-fluent	Recovered	Un-vaccinated	Patient was extremely ill but took his food well even at the worst of his illness. Distribution and maturation of eruption classical. Much exfoliation of cuticle leaving raw surfaces of great extent especially on arms. Recovery uncomplicated with only a few boils. Lost practically all hair and scalp a mass of scar tissue. Received 50 grams sulphadiazine.
6	S. D.	29	M.	2/11/42	5/11/42	5/11/42	Discrete	Recovered	Infancy, School days, and 1940—4 good scars. Re-vaccinated 5/11/42—no take	Although discrete rash very profuse and modification shown by virtual collapse of maturation at commencement of pustular stage, when lesions were represented by prominent fleshy papules with pin-head pustules. Constitutional disturbance disappeared also at this stage. In spite of small size of crusts they were deeply set and were long in being shed. Distribution classical. Received 50 grams sulphanilamide.
7	W. S.	18	M.	4/11/42	5/11/42	5/11/42	Con-fluent	Recovered	5/11/42 primary vaccination—take	A severe case with rash classical in distribution and maturation. Full density not developed till 4th day of rash which by that time was vesicular on part of face. Showed marked respiratory catarrh. Convalescence complicated by boils and abscesses chiefly on buttocks and thighs. Received 60 grams sulphapyridine. Took food well all through.

Case No.	Initials	Age	Sex	Date of onset of Initial Illness	Date of Rash	Date of Admission to Hospital	Type of Rash	Result	History and State of Vaccination	Remarks
8	A. S.	39	M.	5/11/42	6/11/42	6/11/42	Con-fluent	Died 11th day of disease	In 1914–18 2 good scars	A severe case classified as confluent because of this feature of rash on external aspect of left leg from middle of thigh to ankle. Eruption was semi-confluent on face. Distribution fairly typical. Had seven hæmorrhages *per rectum* in last 24 hours before death. Was a poor subject and had a urethral stricture, bilateral hydro-nephrosis and a suprapubic cystostomy. Received 40 grams sulphadiazine.
9	J. P.	37	M.	4/11/42	7/11/42	7/11/42	Discrete sparse	Recovered	Infancy —1 scar. Re-vaccinated 1/11/42 —take. Re-vaccinated 11/11/42 —no take	Rash very sparse and probably consisted of 24 lesions. Distribution fairly typical. A few lesions were typical in size and maturation, but the majority aborted at papular or vesicular stage. Systemic disturbance minimal.
10	R. G.	26	M.	4/11/42	7/11/42	7/11/42	Discrete sparse	Recovered	Infancy —4 good scars. Re-vaccinated 1/11/42 —take	Eruption modified numerically and showed not more than 40 lesions; also modified in maturation as many lesions aborted in vesicular stage. Weight of rash on scapular region but typical seeds appeared on palms and soles. On admission showed remains of erythematous prodromal rash in bathing-drawers area.
11	T. M.	42	M.	4/11/42	7/11/42	Did not come into City Hospital	Hæmorr-hagic	Died 8/11/42 5th day of illness	Infancy —1 scar	—

Case No.	Initials	Age	Sex	Date of onset of Initial Illness	Date of Rash	Date of Admission to Hospital	Type of Rash	Result	History and State of Vaccination	Remarks
12	W. M'K.	17	M.	5/11/42	7/11/42	7/11/42	Confluent	Recovered	Primary Vaccination 4/11/42 —take	A typical case in distribution and maturation. Full density of eruption not developed till 4th day of rash. On arms and thighs there appeared bullae formation involving about half a dozen pocks. These later ruptured with much exfoliation of cuticle, in fact cuticle came away in sheets leaving large areas of raw surface which afterwards healed over very satisfactorily. On face clean skin in orbital fossae gave spectacle appearance in scabbing stage. Patient showed remarkable clarity of mind throughout and appetite remained good — took three-course dinner throughout an exhausting and prolonged secondary fever. Received 30 grams sulphanilamide.
13	P. D.	9	M.	5/11/42	7/11/42	7/11/42	Discrete	Recovered	Primary Vaccination 4/11/42 —take	Illness ran a typical course with a well-marked rash just short of semi-confluent. Initial illness sharp and maturation typical, although rash was slow in appearing on face and in first two days was almost imperceptible there, while well marked on back and upper extremities. Received 40 grams sulphapyridine.
14	W. S.	22	M.	5/11/42	7/11/42	Did not come into City Hospital	Hæmorrhagic	Died 14/11/42, 9th day of illness	Unvaccinated	—

Case No.	Initials	Age	Sex	Date of onset of Initial Illness	Date of Rash	Date of Admission to Hospital	Type of Rash	Result	History and State of Vaccination	Remarks
15	D. D.	14	M.	5/11/42	7/11/42	10/11/42	Semi-confluent	Recovered	Un-vaccinated	A well-marked case with relatively heavy rash, typical in distribution and maturation. This patient had plaster cast on left arm and when this was removed in scabbing stage there was no modification of the rash underneath which was comparable with that on the right arm. The secondary fever was well marked and convalescence was complicated by boils.
16	R. S.	38	M.	4/11/42	8/11/42	10/11/42	Discrete sparse	Recovered	Infancy	Rash very scanty, consisting of about two dozen lesions, small in size and rapidly maturing.
17	J. K.	65	M.	5/11/42	9/11/42	11/11/42	Discrete sparse	Recovered	Infancy and in 1903—1 large scar. Re-vaccinated 12/11/42—no take, and 15/11/42—no take	Lesions very scanty and under 24 in number. Distribution fairly characteristic and majority of lesions small and rapidly maturing, but two attained a fair size. Definite seeds on palmar surface of left thumb and little finger.
18	J. S.	61	M.	7/11/42	10/11/42	10/11/42	Discrete	Recovered	Infancy—1 scar doubtful	Distribution typical but eruption relatively light, and matured rapidly, the face showing large fleshy papules with small crusts. Convalescence complicated by prostatic abscess which raised the question as to whether a lesion had occurred in the vicinity of the prostatic urethra. Received 21 grams sulphadiazine.

Case No.	Initials	Age	Sex	Date of onset of Initial Illness	Date of Rash	Date of Admission to Hospital	Type of Rash	Result	History and State of Vaccination	Remarks
19	A. F.	67	M.	7/11/42	10/11/42	12/11/42	Discrete	Recovered	Infancy—2 scars. Re-vaccinated 12/11/42—no take	Eruption very moderate in amount and modification shown on face by conical fleshy lesions with small yellow crusts on the top. Palmar eruption did not show till 3rd day of rash. Practically no secondary fever.
20	T. K.	57	M.	9/11/42	11/11/42	11/11/42	Discrete sparse	Recovered	Infancy and 1914-18 war—1 good and 2 small scars. Re-vaccinated 6/11/42—take	A very sparse eruption consisting of 9 lesions, 1 on face, 5 on back, and 3 on thigh. Facial lesion typical, of medium size, and matured quickly.
21	J. K.	68	M.	8/11/42	11/11/42	12/11/42	Discrete	Recovered	Infancy—No scars visible. Re-vaccinated 29/10/42—take	Eruption profuse but remained discrete on face. That it was modified was shown by the large conical fleshy bases surmounted by pin-head pustules on the face, which appeared on the 5th day of the rash. Distribution typical except that no lesions appeared on the feet or ankles. Successful revaccination characterised by dark hæmorrhagic blisters followed by deep necrosis. There was a great concentration of lesions round the vaccination area which afterwards exfoliated *en masse*. The final scarring of vaccination sites little different from normal takes. In spite of modification of lesions they were deeply set and scabs took long to come off, patient being last smallpox case to leave hospital. Convalescence much complicated by boils.

Case No.	Initials	Age	Sex	Date of onset of Initial Illness	Date of Rash	Date of Admission to Hospital	Type of Rash	Result	History and State of Vaccination	Remarks
22	M. W.	47	F.	10/11/42	11/11/42	10/11/42	Discrete sparse	Recovered	Primary 1/11/42—take	A greatly modified case with sparse rash and rapid maturation crusting taking place on 8th day of eruption. First appearance of rash at root of neck. Distribution typical. Morbilliform erythema of unknown etiology on legs on 4th day of true rash.
23	J. C. M'K.	79	M.	10/11/42	12/11/42	12/11/42	Discrete sparse	Recovered	Infancy —1 scar. Re-vaccinated 12/11/42 —no take	Rash very scanty avoiding face but present on scalp, hands, including palms and forearms, back and chest. Those on hands and forearms large and typical and matured early. Patient feeble and suffered from enlargement of prostate and arterio-sclerosis.
24	A. F.	17	F.	16/11/42	18/11/42	19/11/42	Discrete	Recovered	Infancy —2 scars. Re-vaccinated 14/11/42 —take	Rash very moderate in amount (16 lesions on face) and typical in distribution. Lesions morphologically characteristic but matured quickly and constitutional disturbance negligible. Crusts superficial and shed quickly. Simple conjunctivitis 16th day of disease.
25	A. C.	24	F.	19/11/42	22/11/42	9/12/42	Discrete	Recovered	Primary 10/11/42 —take	Rash sparse and distribution classical. Maturation typical, but lesions fairly superficial.

Case No.	Initials	Age	Sex	Date of onset of Initial Illness	Date of Rash	Date of Admission to Hospital	Type of Rash	Result	History and State of Vaccination	Remarks
26	H. C.	28	M.	19/11/42	23/11/42	Did not come into City Hospital	Discrete	Recovered	Primary 15/11/42 —take	—
27	E. S.	18	F.	20/11/42	23/11/42	26/11/42	Discrete	Recovered	Primary 14/11/42 —take	Moderate eruption with classical distribution, and secondary fever well marked until crusting supervened. Received 29 grams sulphapyridine. Well marked garter pattern on L. thigh.
28	C. H.	45	F.	20/11/42	23/11/42	25/11/42	Semi-confluent	Recovered	Infancy —2 scars	Patient had been ill for some weeks prior to onset of smallpox and history unreliable. Rash profuse and classical in distribution, semi-confluent on brow and chin, but matured rapidly, crusting on face having taken place on the 7th day of eruption. Crusts pin-head in type and took a long time to come out, those on scapular region being last to separate. Patient showed typical sub-ungual lesions appearing late. Developed simple conjunctivitis. Received 29 grams sulphanilamide.
29	W. G.	35	M.	22/11/42	26/11/42	30/11/42	Discrete	Recovered	Infancy —2 scars. Re-vaccinated 17/11/42 —take	Eruption moderate in density and maturation accelerated or aborted in papular stage. Systemic reaction never marked and patient's chief trouble was a well-marked bronchial catarrh. Received 30 grams sulphapyridine.

Case No.	Initials	Age	Sex	Date of onset of Initial Illness	Date of Rash	Date of Admission to Hospital	Type of Rash	Result	History and State of Vaccination	Remarks
30	J. S.	58	F.	26/11/42	29/11/42	30/11/42	Hæmorrhagic	Died on 10th day of disease	Infancy —1 scar	Rash classical in distribution and character. Became confluent in papular stage and intramural hæmorrhages appeared at commencement of vesicular stage. In late vesicular stage extramural hæmorrhages appeared as well and in the terminal stages large hæmorrhagic blebs appeared scattered on body; on the back especially the cuticle exfoliated leaving large raw surfaces. Cardiac failure was present from the start and patient in coma for the last 48 hours. T. curve atypical. Received 12 grams M. & B. 693.
31	M. C.	58	F.	6/12/42	7/12/42	8/12/42	Hæmorrhagic	Died on 4th day of disease	Infancy —1 good scar	Patient continuously crying out with lumbar pain, obviously very toxic and covered with intense dusky uniform erythema over whole part of body except distal part of legs. Closely resembled scarlet fever except that punctations were rather larger. Later developed irregularly scattered hæmorrhages under skin, deep violet in colour, and size of lentil. Subconjunctival and palatal hæmorrhages. Face leathery but no other sign of focal rash before death. Upper gums edentulous and dead white in colour. Patient, during the first 10 hours she was under observation in hospital remained acutely conscious, but fell into coma 7 hours before death. The hæmorrhages became more profuse and shortly before death a copious uterine hæmorrhage occurred.

Case No.	Initials	Age	Sex	Date of onset of Initial Illness	Date of Rash	Date of Admission to Hospital	Type of Rash	Result	History and State of Vaccination	Remarks
32	J. C.	55	M.	5/12/42	8/12/42	9/12/42	Discrete sparse	Recovered	Infancy and (?) during 1914-18 war—2 good scars and 2 doubtful	Rash very sparse and atypical in distribution in that though the main incidence was on the face there was no differential distribution as between body and limbs, and no lesions on hands or palms, feet or soles. Maturation accelerated and scabs rapidly cast off. Constitutional disturbance mild. Upper respiratory catarrh.
33	M. E. G.	9/12	F.	6/12/42	8/12/42	8/12/42	Discrete	Recovered	Primary on 30/11/42 and 1/12/42 —both takes	Eruption moderate in amount, typical in distribution and maturation. First macules appeared on neck and abdomen and rash not fully developed numerically till it was in its 5th day. Child showed great oedema of face and irritability in pustular stage. Secondary fever well marked. Received 10 grams sulphanilamide.
34	R. G.	59	F.	9/12/42	11/12/42	11/12/42	Hæmorrhagic	Died on 10th day of disease	Infancy —1 scar. Re-vaccinated 28/11/42—no take. 4/12/42 —take	Rash appeared first on trunk, upper arms and thighs, and did not invade face till its 2nd day. By its 4th day, distribution was classical and it was confluent. Intra-mural hæmorrhages appeared on 3rd day of rash, eccymoses on 4th day, lesions seemed to be losing their hæmorrhagic appearance on the 5th day, but when completely vesicular on 6th day the majority assumed a violaceous tinge and several outsize ones were the size of a pea. Epistaxis on 8th day of disease which was almost continuous till death. Apart from original eccymoses all further hæmorrhages were into lesions and at death practically all were hæmorrhagic. Complained greatly of the pain of the rash. Retained consciousness till the last. Laryngitis on 5th day of rash and probably died of pulmonary oedema.

Case No.	Initials	Age	Sex	Date of onset of Initial Illness	Date of Rash	Date of Admission to Hospital	Type of Rash	Result	History and State of Vaccination	Remarks
35	M. B.	55	M.	13/12/42	15/12/42	18/12/42	Hæmorr-hagic	Died on 7th day of disease	Infancy —2 good scars	Intense initial erythema still present on admission involving whole of face, back, and front of body, arms, and thighs. Less intense on lower legs. When first seen was dusky in character, and finally became leaden a few hours before death. Focal rash classical in distribution and confluent in papulo-vesicular stage. Many hæmorrhagic lesions were present varying in size from 2–3 m.m. to 7–8 m.m. on face, body, and limbs. They were deep indigo-violet in colour and many were in vesicles which were flat and poorly filled, while others occurred into the intervening skin. Had one hæmorrhage *per rectum*. Mind clear almost till the last and had no complaint of subjective symptoms. Death appeared to be due to circulatory failure. On the day before admission to hospital had gone out from his house to consult his doctor.
36	M. E. B.	55	F.	28/12/42	29/12/42	30/12/42	Discrete sparse	Recovered	Infancy —1 good scar. Re-vaccinated 18/12/42 —take	Well-marked initial pyrexia, but admitted little in the way of symptoms. Rash sparse and distribution typical. Lesions mainly abortive, some on face fleshy in character. Obviously a modified attack. Patient wife of Case 32, and assuming a 12 day incubation period not infected till the second day of the rash.

APPENDIX III.

POST-VACCINAL ENCEPHALOMYELITIS : CASE HISTORIES AND PATHOLOGICAL DATA

Case 1. P. J. Fem. 2½ years. Primary vaccination 16/7/42. Site of vaccination : inner aspect of left thigh, 2 insertions. 10 days after vaccination (26/7/42) had attack of severe vomiting, and this vomiting occurred intermittently till admission to hospital on 29/7/42. On admission, T. 97° F., P. 112, R. 24. Child critically ill, listless, apathetic, neck rigidity +, Kernig +, slight scoliosis of cervical spine giving appearance of right-sided wry-neck. Later this date, generalised muscular rigidity ensued, T. rose to 102° F., P. 136, and R. 28, Cheyne-Stokes breathing and incontinence of urine and faeces, and patient died early on 30/7/42—4 days from onset of illness.

P.M. Well nourished child. Site of vaccination noted (*supra*), area being about 2·5 cm. by 1·5 cm., well defined and crusted with no vascular or skin reaction around. General post-mortem findings revealed little of interest save a small area of hæmorrhage near right pulmonary apex, and a larger, similar area at posterior aspect of left lower lobe. Microscopically, these areas were proved to be intra-pulmonary hæmorrhages.

C.N.S. Meninges congested and C.S.F. as seen at upper end of cord was bloodstained.

Brain. Vessels congested over whole surface and convolutions flattened on both sides. No evidence of meningitis. Section of brain showed vascular congestion, but no macroscopic pathological lesions.

Special Histological Examination : Haematoxylin eosin, Pickworth, myelin and microglial preparations were made of supra-orbital frontal cortex, parietal and occipital cortex, frontal and parietal white matter, cornu ammonis, basal ganglia, midbrain, pons, medulla and dentate nucleus. Typical lesions found in all these areas, being probably particularly frequent in pons. Lesions showed perivascular and pericapillary demyelination. In and around demyelinated areas, microglial cells were active. Around vessels, whose endothelium was swollen, were relatively thin cuffs of lymphocytes and histiocytes. Outstanding and characteristic picture was the cellular increase for a long distance from vessels. No thrombosed vessels seen. Microglia had a more active appearance in cortex generally, and not only in perivascular aggregations. In the perivascular areas of microglial activity, the nerve cells were remarkably healthy.

Case 2. W. O. Male. 5$\frac{1}{12}$ years. Unsuccessful vaccination in infancy. Vaccination, 20/10/42. Site : left arm. Arm sore and painful on 1/11/42. On 20th day after vaccination (9/11/42) child was seized by a series of generalised convulsions akin to a status epilepticus and which required chloroform to control. 10/11/42 admitted to hospital, T. 97·8° F., P. 136, R. 20. Drowsy, irritable, neck rigidity +, bilateral Kernig ++, slight opiothotonus. All reflexes present, equal but sluggish. All four limbs spastic. No incontinence.

Child steadily improved and recovery was complete, being discharged on 24/11/42—15 days after onset of illness.

Case 3. A. A. Male. 15 years. Primary vaccination 15/11/42. Site: left arm, 2 insertions. Good take, 21/11/42. Arm sore, swollen, and axillary glands tender. 26/11/42, 11 days after vaccination, patient became confused, disorientated, and irritable, with urinary incontinence and increasing drowsiness, which passed into a deep coma. Admitted to hospital same day. On admission, T. 102° F., P. 72, comatose, though physical stimuli roused some response. Scaphoid abdomen. Neck rigidity +, Kernig +, reflexes all present, normal and equal. Plantar flexor responses. Pupils normal, brisk light reaction. 27/11/42: Unchanged, though plantar responses now extensor, and knee jerks ++. Incontinence of urine and faeces supervened later this date. 40 c.c. convalescent whole blood given I–M. 28/11/42: Completely comatose, general muscular flaccidity. Reflexes present, but very sluggish. Incontinent. 29/11/42: T. 100·8° F. Much weaker, loose cough with foul sputum only partially expectorated. I–V drip glucose-saline started. Pressure areas red, with blisters over left femoral trochanter and left ear, which is very swollen. 30/11/42: T. 101·4° F. Coma less deep, tried to speak, swallowed well. 1/12/42: Glucose-saline discontinued, comatose again, bilateral basal consolidation. 2/12/42: Sudden collapse, with death ensuing rapidly, occurring on 7th day of illness.

No P.M.

Case 4. M. H. Fem., 26 years. Successful vaccination in infancy. Vaccination 10/11/42, 3 insertions. 12 days later (22/11/42) shivered, severe frontal and temporal headache. 24/11/42: Headache and pain in neck. 25/11/42: Admitted to hospital. T. 101·8° F., P. 120, R. 24. Frontal headache very severe, no neck rigidity, Kernig +, knee and ankle jerks equal but ++, bilateral extensor plantar responses. Lower limbs spastic. Frequent generalised clonic spasms of body during examination. 28/11/42: T. 101·6° F., improved. 29/11/42: T. 100° F., few papules on chest. 30/11/42: T. 98·4° F., rash fading. No spasticity and reflexes all normal. 12/12/42: Discharged fit on 20th day from onset of illness.

Case 5. Fem., 13 years. Primary vaccination 8/11/42, 3 insertions. 14 days later (22/11/42) irritable, resentful of interference. Admitted to hospital. T. 100° F., P. 120, R. 24. Slight neck rigidity, Kernig +, knee jerks ++, flexor plantar responses. Abdominal reflexes absent; pupils, brisk light reaction. No incontinence. 23/11/42: 40 c.c. whole convalescent blood I–M. T. 99° F. Condition stationary. 24/11/42: Limbs spastic, knee and ankle jerks absent, bilateral Babinski sign; lies passive in bed. 26/11/42: Better, more interested, answers questions, limbs still spastic. 30/11/42: Lower limb reflexes now normal, flexor plantar responses, spasticity almost disappeared; mentally clear. 12/12/42: Discharged fit on 20th day from onset of illness.

Case 6. R. C. Fem., 5 years. Primary vaccination 21/11/42. 13 days later (4/12/42) frontal headache and "pain in stomach." No further history available till onset of coma on 7/12/42, and admission to hospital on 8/12/42.

T. 97·2° F., P. 128, R. 26. Very ill, semi-comatose, no neck rigidity, Kernig –, upper limbs spastic with exaggerated reflexes, lower limbs flaccid with absent reflexes, including plantars, abdominal reflexes absent. Pupils contracted, equal, react to light. 10/12/42 : Consciousness returning, slight neck rigidity, urinary retention, cisternal puncture, nasal feeds. 12/12/42 : T. 102° F., neck rigidity +. Right arm less spastic, left arm more so. Lower limbs still flaccid. Urinary retention persists. 13/12/42 : Head retraction prominent, upper limbs both notably spastic and clonus elicited. Swallowing well; incontinence of urine and faeces. 16/12/42 : Going downhill. T. 103·8° F. Upper limbs very spastic, pupils equal but light reaction very sluggish. 17/12/42 : Semi-comatose. R. rate rising, 48, signs of basal consolidation. 19/12/42 : Died, 15 days from onset of symptoms.

No P.M.

Case 7. A. E. Male, 9½ years. Brother of Case 5. Primary vaccination 14/11/42. Site of vaccination : left arm. ? I insertion. 19/11/42 : Slight frontal headache. 21/11/42 : 7 days after vaccination headache returned, increasing lethargy, would not speak. 22/11/42 : Admitted to hospital. T. 98·2° F., P. 92, R. 24. Completely comatose, neck rigidity ++, Kernig +, general muscular flaccidity, especially in lower limbs where all reflexes are absent. Upper limb reflexes present but sluggish. Pupils very sluggish, light reactions. 40 c.c. of convalescent whole blood I–M. Severe rigor after lumbar puncture. 23/11/42 : T. 99·8° F. Still comatose, but less deeply. Neck rigidity still ++. 24/11/42 : Coma less deep, eyes open, but patient not reacting to vocal stimuli. Pupils contracted. Can be spoon-fed. Swallows well. All reflexes now present, though limbs rather spastic. Flexor plantar responses. Kernig ++. 25/11/42 : Recognised mother, though unable to speak. Nocturnal rise of T. to 104° F. 26/11/42 : Condition very low, general muscular flaccidity. T. 103·6° F. Death on 12th day after vaccination.

P.M. Well developed, left arm showed one brown vaccination scab. No significant changes in general bodily organs, save some degree of acute congestion.

C.N.S. Brain : slightly swollen. Meninges and vessels healthy. Microscopically, brain showed congestion. Pathological lesions in frontal, parietal, occipital and insular cortices, and white matter in putamen, thalamus, substantia nigra, pons and thoracic cord. Changes were perivascular, chiefly perivenular, infiltration of parenchyma with mononuclear cells of histiocyte type, including microglia. Vessels often cuffed with lymphocytes and large monocytes, some of which lie in subarachnoid space. Lesser degree of perivascular demyelination. Nerve cells lying in infiltrated areas remarkably healthy. In cortex, widespread rod cell reaction on part of microglia. Astrocytic nuclei lie within infiltrations but with Mallory's stain there is no evidence of their attempting to effect repair. Chorioid plexus healthy.

No evidence of damage in form of free fat in radial nerve of vaccinated arm.

Case 8. J. T. Male 10 years. Primary vaccination 12/11/42. Site : left arm, ? insertions. 20 days after vaccination, pain in arm, frontal headache, earache. Very restless, noisy, slight head retraction on 4/12/42 when admitted

to local hospital for observation. Sulphapyridine, 1 gramme 4-hourly prescribed and administered for 3 days. 8/12/42 transferred to Edinburgh hospital. T. 98·8° F., P. ?, R. ?. Severe frontal headache, restless, slight neck rigidity, Kernig +, all reflexes present, equal, brisk. Flexor plantar responses. Pupils, medium sized, equal, brisk, light reaction. Urinary retention 9/12/42. T. 100° F., slightly drowsy, headache better, no neck rigidity, Kernig +. 10/12/42. Still drowsy. Urinary retention persists. Progress thereafter was very satisfactory, urinary retention passing off on 12/12/42 and patient was discharged home fit and well on 9/1/43.

Case 9. P.M. Fem. $3\frac{1}{1}\frac{0}{2}$ years. Primary vaccination 18/11/42. Site: left arm, 3 insertions. 11 days later (29/11/42) feverish, listless, apathetic, remained in bed, lay on left side and would not speak. 30/11/42. Admitted to hospital. T. 100° F. Very drowsy but rousable, slight neck rigidity, Kernig +, all reflexes normal and brisk. Pupils react briskly to light. 1/12/42: Incontinent, comatose. 3/12/42: Worse, coma deepening. 5/12/42: T. 97·6° F., general improvement, nods in reply to questions. Still incontinent. 8/12/42: Lies flat on back but replies "yes" and "no" to questions. No incontinence. Constipated. Taking feeds well. Progress continued satisfactorily and though walking was at first difficult, she was discharged home on 30/12/42—31 days from onset of disease.

Case 10. E. C. Fem. $6\frac{1}{2}$ years. Primary vaccination 17/11/42. Site: left arm, ? 3 insertions. 28/11/42: 11 days after vaccination, headache over vertex, abdominal pain, feverishness, obstinate constipation, anorexia, staggering gait forcing child to bed. 29/11/42: Very drowsy. 30/11/42: Admitted to hospital, T. 100·4° F., P. 122, R. 20. Moderate coma, no neck rigidity, Kernig —, general muscular flaccidity with absent reflexes but normal plantar responses. Urinary incontinence. 1/12/42: Still comatose, coughing a lot. Coma deepened, pulmonary embarrassment ensued and death occurred on 6/12/42—8 days after onset of disease.

P.M. Well grown but thin cadaver. Only general pathological findings were some indefinite patches of pneumonic consolidation in left lower lobe.

C.N.S. Brain: Meninges acutely congested with one or two small subarachnoid hæmorrhages over occipital lobe (left). Microscopically: Many blood vessels show infiltrations of inflammatory cells, mostly small mononuclears, in perivascular space, spreading into adjacent brain substance. Considerable vascular congestion but few hæmorrhages. Many nerve cells show degenerative changes. In parts, pia-arachnoid shows infiltration with small round cells, continuous with perivascular infiltrations surrounding blood vessels entering brain. Myelin stains reveal patches of perivascular demyelination. All changes best seen in pons and midbrain, seen in medulla and lentiform nucleus, but not found on section of cerebral cortex.

Case 11. B. F. Fem. $8\frac{1}{2}$ years. Primary vaccination 21/11/42. Site: left arm, ? insertions. 3/12/42: 12 days after vaccination, sudden pain in left arm, neck, and legs which were drawn up. Very excited, flinging arms about, staring wildly. Arm had been sore before this. Admitted to hospital, T. 100° F.

Semi-comatose, general muscular hypertonicity with exaggerated reflexes. Neck rigidity +, Kernig +, bilateral Babinski. Lay in bed with all four limbs in semi-flexion. Urinary incontinence. Choreiform movement of arms on stimulation. Pupils react briskly to light. Vaccination site shows some secondary infection. Abdomen sunken. 4/12/42 : T. 100° F., deeply comatose, critical condition. 6/12/42 : Still comatose though less deeply. 8/12/42 : Improved, T. 98·2° F. Eyes open and following movements around her. Swallowing. Incontinence passing off. 14/12/42 : Continued improvement, but does not speak though understands speech. Arm still septic. No incontinence. Condition steadily improved and patient discharged home fit on 31/12/42—28 days after onset of illness.

Case 12. M. M. Fem., 51 years. Vaccinated successfully in infancy. Vaccination 15/11/42. Site: left arm, 3 insertions. 5 days after vaccination (20/11/42) feverish, "ceaseless revolving of brain," giddiness and "top-heaviness," heaviness of feet which felt cold and dragged. 22/11/42 : Arm felt sore and axillary glands tender. From 23/11/42 to 28/11/42 further symptoms developed, viz., tingling and numbness of feet, diffuse stabbing and shooting pains in legs, which felt even heavier. 29/11/42 : Lethargic. 30/11/42 : Difficulty in walking greater. Urinary retention for 24 hours, but relieved spontaneously that evening. 1/12/42 : Both legs became absolutely powerless and numbness of lower limbs began and spread upwards to about umbilicus—could feel nothing. Urinary retention complete. Constipated. Catheterisation b.d. required. 3/12/42 : Admitted to hospital, T. 97° F., P. 80, R. 20. Complete loss of motor power below level of Th. 6. Sensory loss : pain, light touch, temperature, vibration. Sensory dulling : deep pressure, tendon sense, and joint movements. Pressure sores of each buttock present. Rectal and urinary retention.

Cranial nerves and functions up to Th. 6 level normal. Fundi normal. I-V anti-vaccinal serum given. Blood W.R. -ve. C.S.F. W.R. -ve. Gold Sol. 4432110000. Spinal X-ray negative. Fasting blood sugar 89 mgm. per 100 c.cm. N.P.N. 37%. Chloride 488%. Cholesterol 161%. Condition remained static till 15/12/42, when urinary infection supervened. Bed sores, however, increased and fused into one large necrotic deep ulcer. The urinary infection was controlled with sulphathiazole. 30/12/42 : Slight improvement in sensation as can feel light touch on thighs. 20/1/43 : Automatic bladder emptying occurs occasionally, but urinary infection very troublesome. No improvement neurologically. Bed sore larger and foul smelling in spite of surgical cleaning. Legs becoming oedematous. From 18/2/43 till death on 7/3/43, frequent rigors occurred and patient went steadily downhill, dying on the 108th day of disease.

P.M. Marked oedema of lower limbs, extensive and deep bedsore in sacral region. Lungs were both oedematous and pleural sacs partly filled with clear, yellow, serous fluid. Right pyelonephritis and left pyelitis, acute cystitis were present. Otherwise nothing of note was discovered in general pathology.

C.N.S. No significant abnormality of brain, but sections of lumbar and lower dorsal cord showed marked congestion of grey matter.

Microscopically: Brain : In midbrain a collection of perivascular compound

granular cells and in occipital white matter, areas of perivascular demyelination were only evidence of cerebral pathological changes.

Cord: Widespread degenerative changes, especially in thoracic portion. Large and small areas of myelin thinning or destruction, ill defined, present in white and grey matters and with perivascular pattern. Replacement gliosis was not significant. Fat-laden phagocytes were present in and adjacent to these damaged areas, especially in perivascular spaces of small vessels. In cord grey matter, many nerve cells appeared unhealthy, only a few appeared to be absent. Chromatolysis and nuclear paling present in unhealthy cells. No microglial activity around these cells. Cauda equina showed occasional globules of degenerate myelin in both anterior and posterior roots. In lumbar trunk, slight peripheral nerve degeneration. Many degenerated nerve fibres in common peroneal nerve. Leptomeninges of cord showed no cellular infiltration, but a notable fibrous thickening of the pia-arachnoid.

Case 13. J. L. Fem., 10½ years. Primary vaccination 10/11/42. 21/11/42: 11 days after vaccination, vomiting, listlessness, feverish, increasing drowsiness. 23/11/42: Admitted to hospital. T. 100° F. Very drowsy, photophobia, very irritable. Neck rigidity +, Kernig +, absent abdo. reflexes. Bilateral extensor plantar responses. All other reflexes present. Pupils active. 25/11/42: T. 98·8° F., still drowsy. 27/11/42: Able to sit up and read, but drowsy in evening. 1/12/42–22/12/42 in country convalescent home. Discharged very well, 31 days from onset of disease.

Case 14. M. F. Fem., 4½ years. Primary vaccination 11/10/42. 23/10/42, 12 days later, frontal headache. 24/10/42: Vomiting, lethargic, feverish. 26/10/42: Unable to speak, limbs held stiffly in bed, slight trismus, general muscular hypertonicity. Constipated. Condition remained unchanged and though able to speak on 29/10/42 could not get out of bed because of leg weakness. Incontinence of urine and increasing lethargy necessitated removal to hospital on 30/10/42. On admission, very drowsy, T. 98° F., P. 88, R. 20, limbs spastic. Biceps jerks ++, lower limb reflexes +, bilateral Babinski, absent abdominal reflexes. Active pupils, urinary incontinence. Trismus +. 1/11/42: Condition unchanged, still comatose. 2/11/42: Better, still incontinent. 6/11/42: Normal muscular tonus, no incontinence. Abdominal pain with mucoid diarrhœa. Isolated. 9/11/42: Transferred to Isolation Hospital with Sonné dysentery. Patient made excellent and complete recovery from both diseases.

Case 15. W. U. Male, 10½ years. Primary vaccination 13/11/42. 13 days later (26/11/42), frontal headache. 27/11/42: Very drowsy, generalised epileptiform convulsions (5) in space of 8 hours. Slept between fits. Admitted to hospital. T. 98·2° F., P. 92, R. 24. Very irritable, drowsy, when first seen, but recovered rapidly after admission to ward and answered questions clearly. Reflexes all normal. Pupils active. Progress was maintained and patient discharged very well on 6/12/42—10 days after onset of disease.

Case 16. J. S. Fem., 6 10/12 years. Primary vaccination 10/11/42. Site: arm (left), 3 insertions. 21/11/42: 11 days after vaccination, drowsy. 23/11/42:

Drowsiness increased, pain in abdomen and back. Neck stiff and held rigidly, whole body inclined to be held stiffly. Appeared deaf and could not speak. During night of 23–24/11/42 incessant hiccough. 24/11/42 : Comatose, urinary and faecal incontinence. Admitted to hospital. T. 99° F., P. 109, R. 30. General muscular hypertonus, Trismus, neck rigidity ++, Kernig +, absent abdominal reflexes, extensor plantar responses, but other reflexes present. 26/11/42 : Lethargic, sips water, still double incontinence. 27/11/42 : More awake, speaking. 28/11/42 : Incontinence relieved. Progress continued apace, but legs were rather stiff and walking difficult. Some urinary frequency. After spell in convalescent home, patient discharged fit on 19/1/43.

Case 17. M. G. Fem., 28 years. Primary vaccination 7/11/42. Site : left arm, 3 insertions. 29/11/42 : 22 days after vaccination, shivery, backache, and pain in calves of legs. 30/11/42 : Legs weak, could stand with support but not walk. 1/12/42 : As before, headache constant, very drowsy, urinary retention. In bed. 3/12/42 : Admitted to hospital in a coma from which she was rousable. Slight neck rigidity, Kernig +, head tender on percussion, irritable. Loss of power in lower limbs, but reflexes present, with flexor plantar responses. Urinary retention. Slight papilloedema bilaterally. 5/12/42 : Given convalescent serum I-M, but patient already recovering. Progress continued and though urinary retention was troublesome for a time, patient was discharged fit after a short stay in hospital.

Case 18. J. H. Male, 15½ years. Primary vaccination 21/12/42. Site : left arm, ? insertions. On 28/12/42, "out-of-sorts," anorexia, listless, severe headache. 3/1/43 : Increasing drowsiness going on to coma. Urinary retention. Admitted to hospital, T. 101° F. Comatose, irritable, neck rigidity +, Kernig +, all reflexes present, plantar flexor responses. Pupils active, fundus normal. Bladder to umbilicus. Herpes labialis. Left axillary gland enlargement associated with well-defined vaccinial reaction. 20 c.c. convalescent serum I-V. Patient's condition steadily deteriorated, lower limbs becoming flaccid and reflexes disappeared, followed by loss of abdominal reflexes. Respirations, as coma deepened, became diaphragmatic. T., P., and R. rose progressively, and though some improvement was evident on 4/1/43, when he was able to swallow, it was evanescent. 6/1/43 : Right pneumonic consolidation present. 20 c.c. convalescent serum I-V + sulphapyridine in glucose saline drip given without improvement. Died 7/1/43—10 days from onset of disease.

P.M. Poorly nourished. Black scab, size of shilling, on left arm. General pathological findings were : Adherent right pleura (fibrous adhesions) early diffuse consolidation of right lung, congestion of left lung ; distended urinary bladder, with some dilatation of ureters and kidney pelves, which also showed few petechiæ.

C.N.S. Brain : flattening of convolutions present, surface vessels congested. Section of brain in region of frontal lobes showed dilated vessels and small hæmorrhages in both white and grey matters. Spinal cord showed similar but less obvious changes.

Microscopically: Pickworth preparations made of—parietal and occipital regions, basal ganglia, cornu ammonis, upper medulla, cervical and lumbar

cord sections. All showed notable congestion. In parietal and occipital white matter, thalamus, putamen, claustrum, external capsule, medulla, cervical and especially lumbar cord, there were perivascular infiltrations of granular cells. Cellular infiltrations more peri-capillary and peri-venular than peri-arteriolar. Perivascular infiltrations associated with perivascular demyelination. No true hæmorrhages noted.

Chief cellular reaction was of collections of lymphocytes and monocytes in perivascular spaces and infiltration of adjacent damaged parenchyma with large mononuclears and reacting microglia. Nerve cells in damaged areas comparatively healthy. Though astrocytic nuclei were seen they did not appear to be attempting repair by laying down glial fibrils. Cortical involvement was seen in frontal and insular areas, where it was associated with fairly diffuse " rod cell " microglial reaction.

Case 19. J. S. Fem., $6\frac{10}{12}$ years. Primary vaccination 11/11/42. Site: left arm, ? 1 insertion. Severe local vaccinial reaction 5 days later. 24/11/42: Definite nervous involvement, drowsiness, bout of diarrhœa. 25/11/42: 5 epileptiform convulsions with stupor *inter* fits, vomiting, feverishness. Admitted to hospital. T. 99·2° F., P. 66, R. 24, deeply comatose, incontinence of urine and faeces. No neck rigidity, Kernig ?+, reflexes present in upper limbs, absent in lower limbs, bilateral extensor plantar responses. Pupils insensitive. 26/11/42: T. 100° F. Drowsy, still incontinent, swallowing well. 29/11/42: T. 97° F. Still incontinent of urine, but not of faeces. Knee and ankle jerks absent. Babinski present bilaterally. 1/12/42: T. 97° F. Continent of urine. All reflexes normal, alert and bright. 4/12/42: Discharged fit—10 days after onset of illness. Reported later as out-patient fit and well. No symptoms nor signs of disease.

Case 20. E. H. Fem., $4\frac{7}{12}$ years. Primary vaccination 11/11/42. Site: left arm, 1 insertion. 14 days after vaccination (25/11/42) vomiting, headache, increasing drowsiness. 29/11/42: Comatose. 3 generalised convulsions within a few hours time. Admitted to hospital. T. 97·8° F., P. 100, R. 22. Comatose, reflexes all present, flexor plantar responses, active pupils. Urinary incontinence. 30/11/42: Drowsy, still urinary incontinence, no further changes in C.N.S. 2/12/42: Incontinence cleared up, child well. 9/12/42: Discharged home well. Reported as out-patient on 21/12/42 when described as perfectly well.

Case 21. T. C. Male, 5 years. Primary vaccination 9/11/42. Site: left arm, 1 insertion. 11 days later (20/11/42) headache, abdominal pain, increasing drowsiness. 23/11/42: Drowsiness worse, urinary incontinence. 24/11/42: Semi-comatose. Admitted to hospital. T. 100·4° F., P. 108, R. 26. Irritable, neck rigidity +, Kernig +, all reflexes absent, bilateral Babinski. Urinary incontinence. Pupils active. 25/11/42: T. 97° F., drowsy but recovering, and swallowing well. Neck rigidity +, Kernig +, urinary incontinence. 26/11/42: T. 97° F. All reflexes normal, urinary incontinence persists. 29/11/42: Continued improvement, but still incontinent. 3/12/42: Continent. Very well. No physical signs or symptoms of disease. 8/12/42: Discharged well. Reported as out-patient on 22/12/42 with chickenpox.

Case 22. E. G. Fem., 5 years. Primary vaccination 24/11/42. Site: left arm, 3 insertions. From 27/11/42 to 3/12/42 arm sore and tender, some general upset. 5/12/42 : 11 days after vaccination, listless, increasing drowsiness. 6/12/42 : I.S.Q. 7/12/42 : Drowsy, vomiting, headache. 8/12/42 : Drowsiness, more marked, Anorexia. 9/12/42 : Delirious, 2 fits, coma, urinary incontinence. Admitted to hospital. T. 98·6° F., P. 140, R. 36. Comatose. All reflexes absent, bilateral extensor plantar responses. Pupils dilated and inactive. No neck rigidity, Kernig —, urinary and rectal incontinence. 10/12/42 : T. 98·6° F. Brighter, though drowsy. Reflexes all present, Babinski +, urinary and rectal incontinence persists. 11/12/42 : No incontinence now. Reflexes all normal. 15/12/42 : Discharged, 10 days after onset of symptoms. 28/12/42 : Reported well.

Case 23. E. R. Fem., 30 years. Secondary vaccination 18/11/42. Site : left arm, ? 3 insertions. 21/11/42 : Felt "queer," weakness in legs, headache, dizziness, arm not painful. 22/11/42 : No complaints. 23/11/42 : Nausea, but no vomiting. 24/11/42 : Very restless previous night, feverish. 25/11/42 : Very drowsy, "eyes staring," died suddenly.

No P.M.
